# Chronic lung inflammation primes humoral immunity and augments antipneumococcal resistance

**DOI:** 10.1038/s41598-017-05212-4

**Published:** 2017-07-10

**Authors:** Julia D. Boehme, Sabine Stegemann-Koniszewski, Andrea Autengruber, Nicole Peters, Josef Wissing, Lothar Jänsch, Andreas Jeron, Dunja Bruder

**Affiliations:** 1 Infection Immunology Group, Institute of Medical Microbiology and Hospital Hygiene, Otto-von-Guericke University, Magdeburg, Germany; 2 Immune Regulation Group, Helmholtz Centre for Infection Research, Braunschweig, Germany; 3 Cellular Proteomics Group, Helmholtz Centre for Infection Research, Braunschweig, Germany

## Abstract

Airway epithelial cells (AECs) display remarkable plasticity in response to infectious stimuli and their functional adaptations are critical for antimicrobial immunity. However, the roles of AECs and humoral mediators to host defense in non-communicable lung inflammation remain elusive. We dissected pulmonary defense against *Streptococcus pneumoniae* in hosts with pre-existing inflammatory conditions (SPC-HAxTCR-HA mice). Lung tissue transcriptomics and bronchoalveolar lavage fluid (BALF) proteomics revealed an induction of humoral defense mechanisms in inflamed lungs. Accordingly, besides antibacterial proteins and complement components being overrepresented in inflamed lungs, elevated polymeric immunoglobulin receptor (pIgR)-expression in AECs correlated with increased secretory immunoglobulin (SIg) transport. Consequently, opsonization assays revealed augmented pneumococcal coverage by SIgs present in the BALF of SPC-HAxTCR-HA mice, which was associated with enhanced antipneumococcal resistance. These findings emphasize the immunologic potential of AECs as well as their central role in providing antibacterial protection and put forward pIgR as potential target for therapeutic manipulation in infection-prone individuals.

## Introduction

Non-communicable chronic respiratory diseases (CRDs) are multifactorial disorders with different etiologies which manifest in pulmonary structural and/or functional changes. Community-acquired pneumonia caused by *Streptococcus pneumoniae* is a major comorbidity in CRDs^1^.

In healthy lungs, a plethora of physical, humoral and cellular mechanisms synergistically counteract pneumococcal adhesion, outgrowth and tissue invasion thus ensuring homeostasis and functional integrity.

The lung epithelium - as a key constituent of the lung mucosal surface – is critical for host defense. Besides providing a fairly impermeable physical barrier against bacterial pericellular migration, epithelial cells produce antimicrobial proteins (APs)^2^. Moreover, they secrete complement components^3^ and complement critically contributes to immunity towards respiratory bacterial infection^4^.

By directed cooperation with the humoral immune system respiratory epithelial cells provide broad, unspecific protection against a multitude of airborne pathogens. While submucosal plasma cells produce natural, mostly dimeric, IgA^5^ it is still a matter of debate which B cell subsets contribute to local and systemic natural IgM levels^6^. Both immunoglobulin subtypes share a common structure: the joining chain (J chain). Upon binding of the J chain by the polymeric immunoglobulin receptor (pIgR), expressed by respiratory epithelial cells, transcytosis of the pIgR-antibody complex through the epithelium is initiated. After proteolytic cleavage IgA and IgM are released into the airways, bound to a small pIgR-subunit, the secretory component. By binding to bacterial surfaces natural IgA inhibits pathogen adhesion and invasion of epithelial cells^5^, a process known as immune exclusion. Furthermore, early antibacterial activity is mediated by concerted actions of IgM and complement component C1q^4^. *In vivo* studies evidence crucial roles for pIgR and secretory immunoglobulins (SIgs) in host immunity towards mucosal pathogens^4, 7, 8^. Of note, more recent studies introduced the concept of stimulated pulmonary resistance, i.e. enhancement of antimicrobial efficacy following respiratory inflammatory priming by administration of TLR ligands or primary infection. These reports suggested an enhancement of leukocyte effector mechanisms as well as augmented airway epithelial microbicide production^9–12^. Still, details on the mechanisms underlying improved vs. blunted antimicrobial defense in lung inflammation remain elusive.

For a more comprehensive understanding regarding inflammation-related pulmonary adaptations during sterile inflammation, we utilized a very well-established mouse model for chronic lung inflammation (SPC-HAxTCR-HA mice^13–15^) to perform in-depth characterization of the lung microenvironment. SPC-HAxTCR-HA mice express the influenza A virus hemagglutinin (HA) as a neo-self-antigen under the control of the surfactant protein C promoter which is exclusively active in alveolar type II epithelial cells (SPC-HA mice). Alongside, these mice harbor HA-specific CD4^+^ T cells responding to the HA-antigen in the lung (TCR-HA mice) and thus causing a T cell-mediated autoimmune inflammation shortly after birth. Comprehensive transcriptional and proteomic analyses revealed that chronic lung inflammation locally induces a set of humoral antimicrobial mechanisms including mediators orchestrating secretory immunoglobulin-mediated immunity. In line with increased pIgR and SIg levels we found augmented opsonizing capacity of lung mucosal fluid in SPC-HAxTCR-HA mice that was associated with improved antipneumococcal resistance. Altogether, we propose inflammation-enhanced SIg-transcytosis as an epithelial mechanism that directly counteracts pneumococcal adhesion and invasion.

## Results

### Chronic lung inflammation induces a B cell-specific signature

**Figure 1:**
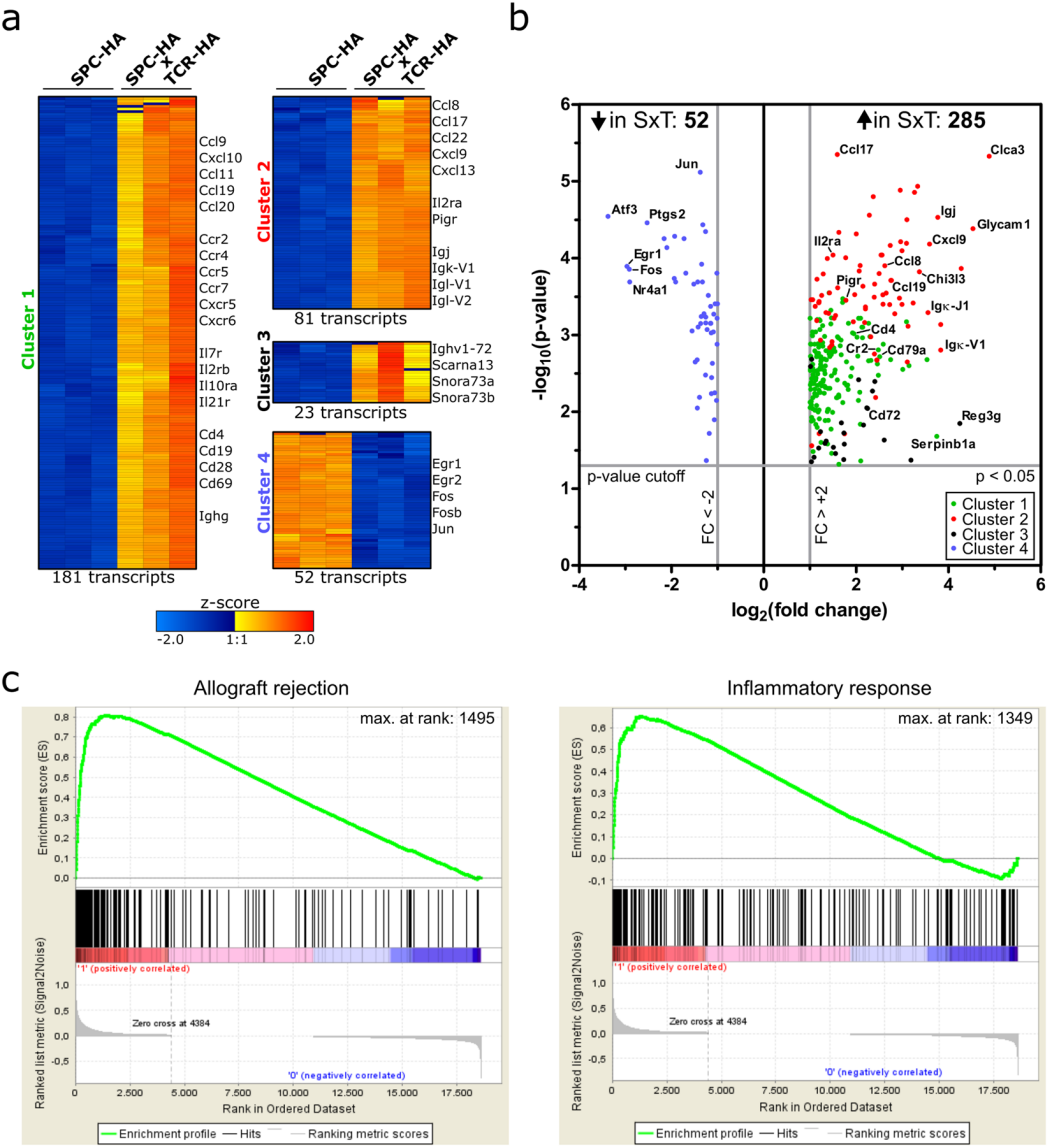
Transcriptional profile of whole lung tissue from SPC-HAxTCR-HA vs. SPC-HA mice. RNA from lung tissue of SPC-HA and SPC-HAxTCR-HA mice (n = 3/group) was isolated and samples were individually analyzed on whole transcriptome microarrays. Differential expression was analyzed by one-way ANOVA with p-value cutoff p < 0.05 and fold change cutoff FC > ±2 comparing SPC-HAxTCR-HA vs. SPC-HA. Fold changes were calculated based on group-average of signal intensities. (**a**) Normalized log_2_ signal intensities of significantly regulated transcripts from each individual microarray replicate were clustered according to k-means clustering (cluster 1–4). Color code represents z-score. Gene symbols of selected prominent transcripts within each cluster are stated. (**b**) Volcano **s**catter plot of significantly regulated transcripts with cluster assignment referring to (**a**). Gene symbols of selected prominent transcripts are stated. SxT = SPC-HAxTCR-HA; FC = fold change. (**c**) Gene Set Enrichment Analysis (GSEA) for canonical hallmark gene sets from the Molecular Signature Database (MSigDB) of microarray data comparing SPC-HAxTCR-HA vs. SPC-HA (FDR < 5%).

In previous studies we have extensively characterized the double transgenic SPC-HAxTCR-HA mouse model^13–15^, in which the recognition of an alveolar neo-self-antigen by simultaneously produced self-antigen specific CD4^+^ T cells results in chronic lung inflammation. Histologically, SPC-HAxTCR-HA transgenic mice share several features of chronic-progressive interstitial pneumonitis in people with immunopathological causes. The lesions are characterized by massive diffuse infiltration of interalveolar septa, predominantly surrounding airways and blood vessels, with lymphocytes and, to a lesser degree, plasma cells. Infiltrating cells were identified as self-reactive but also regulatory CD4^+^ T cells^13^. In more mature mice beyond a few months of age, the more diffuse infiltrates develop into lymphoid follicles surrounding airways and vessels. While we have shown that chronic lung inflammation induces distinct immunoregulatory mechanisms - with vital roles of CD4^+^Foxp3^+^ regulatory T cells and alveolar epithelial cells^14, 15^ - the impact of the local inflammatory microenvironment on host immunity towards airborne infection remains elusive. To identify molecular mechanisms with possible implications for antimicrobial resistance in the inflamed lung we performed unbiased genome-wide transcriptome analyses of lung tissue from healthy SPC-HA and diseased SPC-HAxTCR-HA mice. Inflammation in SPC-HAxTCR-HA vs. SPC-HA lungs was associated with up-regulation of 285 and down-regulation of 52 transcripts separating into 4 k-means gene clusters (Fig. 1a and b). Moreover and well in line with the established autoimmune-mediated lung inflammation in these mice, Gene Set Enrichment Analysis (GSEA) of microarray data demonstrate a significant enrichment (FDR < 5%) for genes involved in allograft rejection and inflammatory responses in SPC-HAxTCR-HA lungs (Fig. 1c, Supplementary Table S4, Supplementary Fig. S5). Remarkably, among the 20 most intensely up-regulated genes 15 were immunoglobulin-related, including *Igj*, which encodes for the J chain (Supplementary Table S1). Likewise, pathway ontology analyses revealed highly significant over-representation of genes associated with the terms *B cell receptor signaling pathway* and *Intestinal immune network for IgA production* (Supplementary Table S3). Interestingly, several genes with known antimicrobial functions were induced, including the polymeric immunoglobulin receptor (*Pigr*), peptidoglycan recognition protein 1 (*Pglyrp1*), lipocalin 2 (*Lcn2*), regenerating islet-derived 3 gamma (*Reg3g*) as well as complement components (Table 1). These mediators are typically expressed in leukocytes (*C1q*)^16^, epithelial cells (*Pigr*, *Reg3g*)^17, 18^ or both cell types (*Pglyrp1*, *Lcn2*)^19, 20^, respectively. Thus, chronic inflammatory imprinting encompasses substantial alterations in the pulmonary transcriptome suggesting a markedly changed cellularity and the induction of distinct pathways involved in humoral, antimicrobial immunity. Indeed, in line with our previous observations on the basis of histopathological examination^13^ analysis of BALF cellular content showed significantly elevated numbers of T and B cells in SPC-HAxTCR-HA compared to SPC-HA mice. However, the numbers of AMs, neutrophils and NK cells in the BALF were comparable in both genotypes (Supplementary Fig. S4).

**Table 1:** Comparative proteomic and transcriptomic analyses of antimicrobial proteins in SPC-HAxTCR-HA vs. SPC-HA lungs.

Gene symbol	Protein	Transcript Cluster ID	SwissProt Accession ID	Analyses	
				BALFome	Microarray (FC)
*Pigr*	Polymeric immunoglobulin receptor	10531126	O70570	x	x (3.4)
*Igj*	Immunoglobulin joining chain	10531126	P01592	x	x (13.6)
*Igh-VJ558*	Immunoglobulin alpha chain C region	10402864	P01878	x	x (3.9)
*Ltf*	Lactotransferrin		P08071	x	
*Camp*	Cathelin-related antimicrobial peptide		P51437	x	
*Sftpa1*	Pulmonary surfactant-associated protein A		P35242	x	
*Ighm*	Immunoglobulin mu chain C region		P01872	x	
*Pglyrp1*	Peptidoglycan recognition protein 1	10550509			x (4.4)
*C1qc*	Complement component 1, q subcomponent, C chain	10517513			x (3.1)
*C1qb*	Complement component 1, q subcomponent, beta polypeptide	10517508			x (2.8)
*Cfb*	Complement factor B	10450325			x (2.7)
*C1qa*	Complement component 1, q subcomponent, alpha polypeptide	10517517			x (2.7)
*Cfp*	Complement factor properdin	10603860			x (2.5)
*Lcn2*	Lipocalin 2	10481627			x (2.0)
*Reg3g*	Regenerating islet-derived 3 gamma	10545569			x (5.3)

Definition of abbreviations: FC = fold change, x = protein uniquely detected in SPC-HAxTCR-HA BALFome, also: transcript induced in SPC-HAxTCR-HA lungs.

### Increased abundance of humoral mediators in the inflamed airways

**Figure 2:**
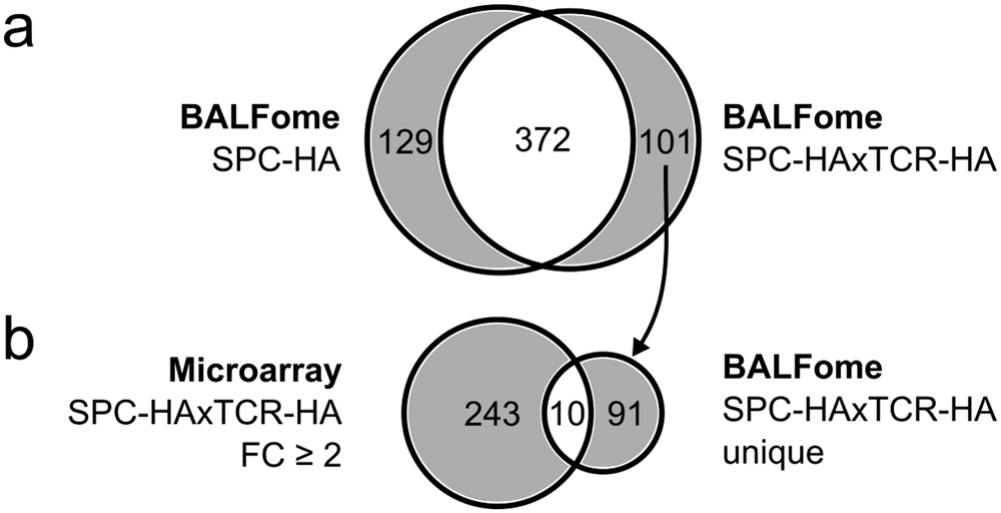
Chronic lung inflammation alters the BALF proteome. (**a**) Venn diagram comparing LC-MS/MS-identified proteins from bronchoalveolar lavage fluid (BALF) of SPC-HA vs. SPC-HAxTCR-HA mice. Samples were pooled from 5 mice/group. (**b**) Venn diagram comparing genes with ANOVA p-value p < 0.05 and a fold change FC > ±2 in lungs of SPC-HAxTCR-HA mice vs. genes (identified by their respective products) only detected in BALF from SPC-HAxTCR-HA mice.

To further corroborate that sterile lung inflammation is associated with a local overrepresentation of mediators related to antimicrobial and humoral immunity, we performed qualitative proteome analyses of bronchoalveolar lavage fluid (BALF). BALFome analyses identified 602 proteins of which ~62% (372 proteins) were detected in both groups, while 21% (129 proteins) and 17% (101 proteins) were uniquely assigned to SPC-HA BALF or SPC-HAxTCR-HA BALF, respectively (Fig. 2a, Supplementary Tables S5–S7). Interestingly, the antimicrobials cathelin-related antimicrobial peptide, pulmonary surfactant-associated protein A1 and lactotransferrin were exclusively detected in SPC-HAxTCR-HA BALF (Table 1).

The comparison of up-regulated, annotated transcripts from SPC-HAxTCR-HA lungs (253 gene symbols) with unique proteins in SPC-HAxTCR-HA BALF yielded an overlap of 10 proteins (Fig. 2b). Notably, and in line with the transcriptome data, out of these, we identified 3 proteins associated with secretory antibody-mediated immunity: immunoglobulin alpha chain C region, immunoglobulin J chain and pIgR (Table 1). Collectively, these proteome analyses supported the hypothesis of an enhancement of humoral immunity in chronically inflamed lungs.

### Elevated pIgR- and secretory antibody levels in inflamed lungs

**Figure 3:**
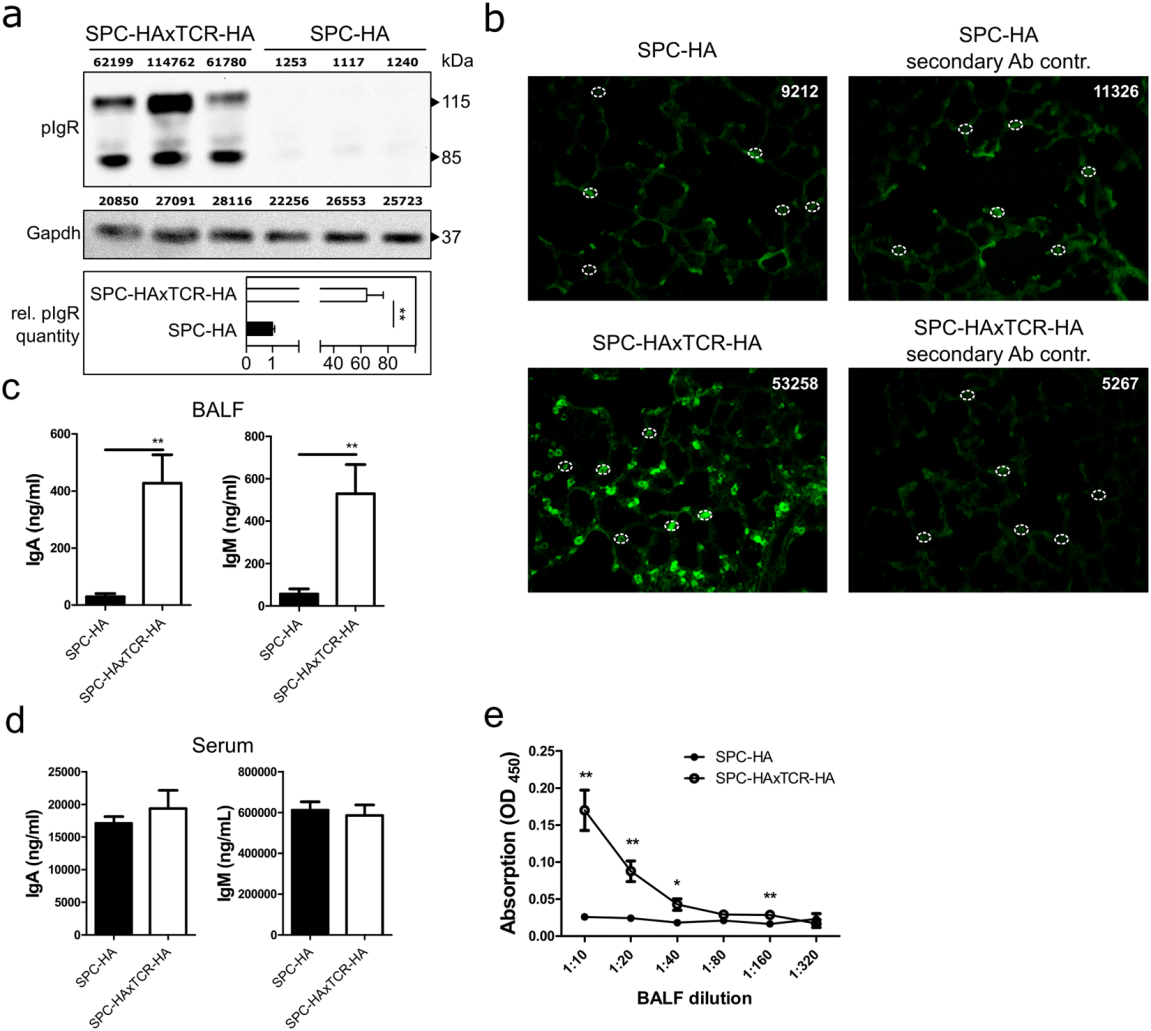
Increased mucosal transport of secretory antibodies in inflamed lungs. (**a**) anti-pIgR and anti-Gapdh immunoblot of 20 µg protein from whole lung homogenates of SPC-HA (n = 3) and SPC-HAxTCR-HA (n = 3) mice. Densitometric quantification of protein bands is stated in arbitrary units above each lane. Relative pIgR quantity was calculated normalizing densitometric pIgR value to the corresponding Gapdh value and subsequently comparing normalized pIgR values of the SPC-HAxTCR-HA group to the SPC-HA group. Data are representative for at least two individual experiments with similar results. (**b**) Lung tissue sections were stained with anti-pIgR (green), representative alveolar structures from n = 3/group are depicted. White circles illustrate representative densitometrically quantified tissue areas. Calculated total cell fluorescence (CTCF) was determined as: Integrated density of fluorescence-positive cell – (Area of fluorescence-positive cell × mean fluorescence intensity of background signal). Median CTCF of quantified areas in representative images are depicted as white numbers. IgA and IgM levels in (**c**) bronchoalveolar lavage fluid (BALF) and (**d**) serum of SPC-HA and SPC-HAxTCR-HA mice were determined by ELISA. (**e**) Relative secretory IgA concentrations in serial dilutions of BALF samples were determined by ELISA. Results are expressed as the mean optical density (OD) at 450 nm ± SEM, *p < 0.05, ** p < 0.01 (n = 6–7/group).

Prompted by the secretory antibody-signature identified by proteomic and transcriptomic profiling, we quantified the expression of pIgR, the exclusive transport molecule of secretory IgA and IgM, in the lungs and found 3-fold upregulated mRNA (Supplementary Fig. S1a) as well as 64-fold upregulated protein (Fig. 3a) levels in SPC-HAxTCR-HA mice. We localized increased pIgR expression in the alveoli of SPC-HAxTCR-HA mice, where it was restricted to single, cuboidal epithelial cells (Fig. 3b).

In line with epithelial pIgR up-regulation we found elevated IgA and IgM levels in BALF of SPC-HAxTCR-HA mice compared to SPC-HA mice (Fig. 3c). However, serum IgA and IgM levels were similar between both groups (Fig. 3d), signifying that elevated BALF IgA and IgM contents most likely arose from increased intrapulmonary transport in SPC-HAxTCR-HA mice. In light of the fact that increased albumin levels in SPC-HAxTCR-HA BALF (Supplementary Fig. S2) suggests enhanced passive diffusion of serum components, we experimentally addressed whether the increase in BALF IgA and IgM is indeed due to active transcytosis through the epithelial layer. To this end, we compared the abundance of SIgA in BALF by ELISA using an anti-IgA capture antibody and an antibody specifically binding to the secretory component of the pIgR molecule for detection. Indeed, significantly higher mean ODs were detected in BALF samples from SPC-HAxTCR-HA mice (Fig. 3e), confirming amplified active transepithelial transport of SIgA.

### BALF from chronically inflamed lungs has an increased opsonizing capacity

**Figure 4:**
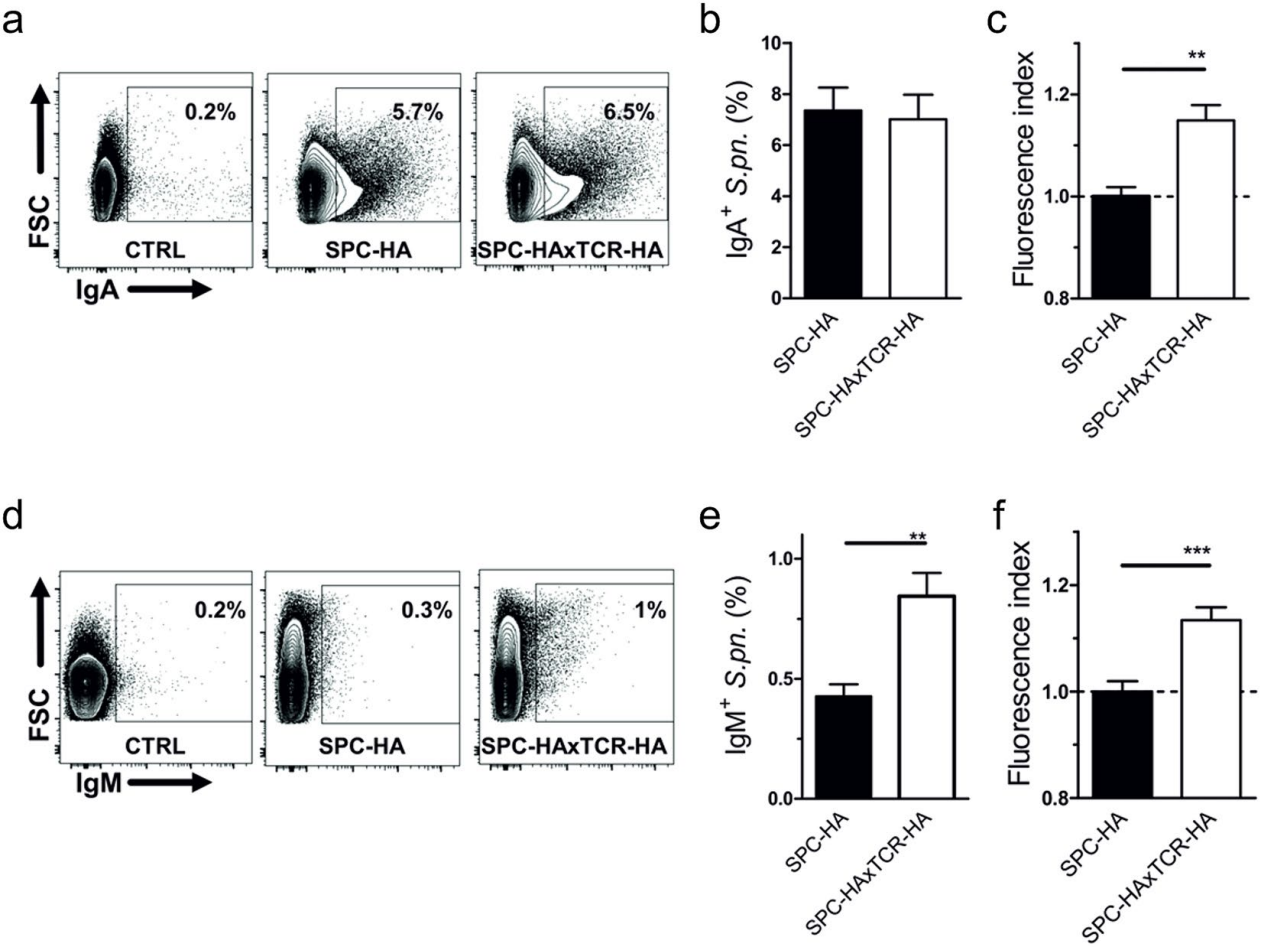
Increased pneumococcal binding capacities by lung mucosal fluid in inflamed lungs. Pneumococci were co-incubated with bronchoalveolar lavage fluid (BALF) supernatants from SPC-HA and SPC-HAxTCR-HA mice. Bacteria were stained with anti-mouse IgA or anti-IgM antibodies and analyzed by flow cytometry (FACS). (**a**) Representative FACS plots of IgA+ pneumococci incubated with BALF from SPC-HA or SPC-HAxTCR-mice; control samples (CTRL) were stained with anti-IgA without prior incubation with BALF. (**b**) Percentages of IgA+ pneumococci and relative fluorescence intensities (**c**) of IgA+ pneumococci. (**d**) Representative FACS plots of IgM+ pneumococci incubated with BALF from SPC-HA or SPC-HAxTCR-mice; control samples (CTRL) were stained with anti-IgM without prior incubation with BALF. (**e**) Percentages of IgM+ pneumococci and relative fluorescence intensities (**f**) of IgM+ pneumococci. Relative fluorescence intensities are calculated by the ratio of the MFI of each individual sample over the mean MFI of the SPC-HA control group. Data are pooled from 2 independent experiments with similar results. *p < 0.05 **p < 0.01, ***p < 0.001.

To test a possible influence of elevated SIg levels on antimicrobial resistance we opsonized *Streptococcus pneumoniae* with BALF supernatants and identified antibody-carrying bacteria by surface staining against IgA (Fig. 4a) or IgM (Fig. 4d), respectively. While similar fractions of IgA-positive pneumococci following incubation with both SPC-HAxTCR-HA as well as SPC-HA BALF (Fig. 4b) were observed, significantly higher IgA deposition on pneumococci that were incubated with BALF from SPC-HAxTCR-HA mice was detected (Fig. 4c). Moreover, opsonization with SPC-HAxTCR-HA BALF yielded increased portions of IgM-positive pneumococci as well as enhanced IgM deposition when compared to SPC-HA BALF (Fig. 4e and f). Of note, *in vitro* performed pneumococcal killing assays in which bacteria were incubated for up to 90 min in BALF from SPC-HA mice, SPC-HAxTCR-HA mice or PBS did not demonstrate marked anti-bacterial capacity of either BAL fluids compared to the PBS control (Supplementary Fig. S6). Thus, increased pIgR-mediated pulmonary IgA and IgM transport may considerably improve the mucosal barrier during respiratory pneumococcal infection, independent of soluble APs initially present in the BALF.

### Chronic lung inflammation does not enhance alveolar phagocyte recruitment, bacterial binding capacity nor complement activation in early *Streptococcus pneumoniae* infection

**Figure 5:**
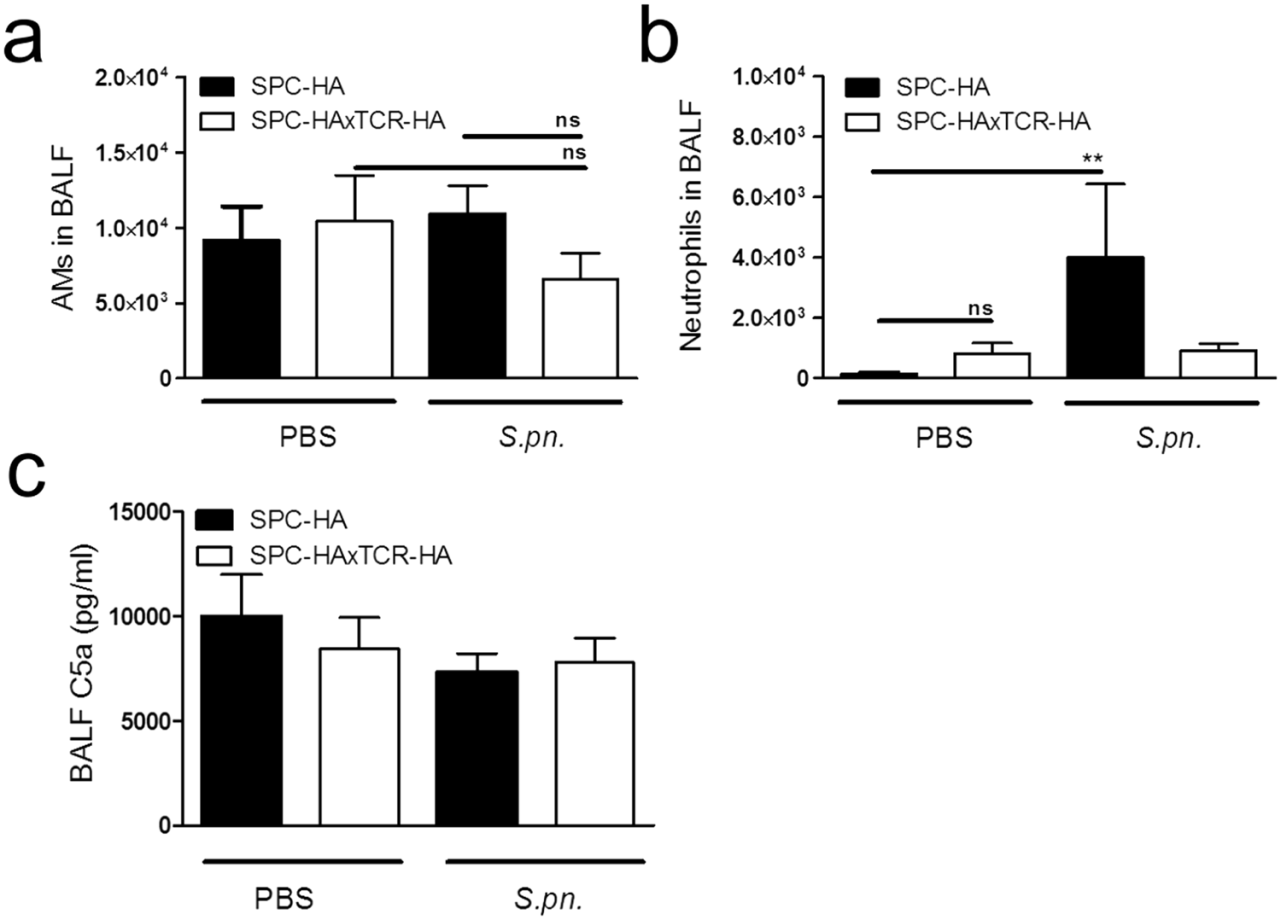
Improved bacterial clearance is not associated with increased AM numbers nor with increased intra-pulmonary neutrophil recruitment in SPC-HAxTCR-HA mice. Mice were oropharyngeally inoculated with ~1.5 × 10^4^ CFU *S*. *pneumoniae* TIGR4 or were mock-treated (PBS). At 24 h p.i. mice were sacrificed and phagocytic leukocyte subsets in the BALF (**a**,**b**) were quantified by flow cytometry. (**c**) BALF supernatants were anylyzed by ELISA for levels of complement component C5a. Data were pooled from at least 2 independent experiments (n = 5–8/group). **p < 0.01.

To assess whether inflammation-primed alterations of the respiratory micro-milieu in SPC-HAxTCR-HA mice would affect early innate immune responses in the alveolar space following bacterial encounter *in vivo*, we next analyzed the influx of phagocytic cells into the alveolar space of BALF of SPC-HA and SPC-HAxTCR-HA mice 24 h after *Streptococcus pneumoniae* infection. We did neither observe differences in the number of AMs and neutrophils in the airways of both mouse groups prior infection (Fig. 5a and b) nor did the number of AMs change within 24 h after pneumococcal challenge in SPC-HA and SPC-HAxTCR-HA mice (Fig. 5a). Interestingly, upon infection SPC-HA mice showed a significant increase in alveolar neutrophil counts which was absent in SPC-HAxTCR-HA mice (Fig. 5b). In addition to unaltered AM numbers, comparison of immediate *in vivo* association of fluorescently labelled pneumococci to AMs did not reveal any differences in the capacity of AMs to bind opsonized bacterial cells (Fig. S3).

Since IgA and IgM opsonization of bacteria can initialize complement activation we next analyzed alveolar C5 activation by quantifying C5a in BALF of both mouse strains following *Streptococcus pneumoniae* infection. C5a concentration did not increase within 24 h of pneumococcal infection and was comparable between the two genotypes (Fig. 5c), suggesting that elevated IgA/IgM concentrations in BALF from SPC-HAxTCR-HA mice do not contribute to enhanced immune-complex-driven complement activation within 24 h of *Streptococcus pneumoniae* infection.

### Increased antipneumococcal resistance in mice with chronic lung inflammation

In order to ultimately determine whether the observed inflammation-driven pulmonary adaptions would affect pathogen replication in the respiratory tract and the overall outcome of infection mice were challenged with an invasive strain of *Streptococcus pneumoniae* (TIGR4) that exhibits a high invasive disease potential. Here, death from infection is preceded by pneumococcal tissue invasion, subsequent bacteremia and eventually the development of lethal sepsis^21^. To exclude the possibility that diffused bloodstream-derived pneumococci at high infectious doses would affect the results of the actual bacterial load in the bronchoalveolar spaces a sub-lethal infection dose, which typically does not cause bacteremia within 24 h, was chosen for experiments aiming at quantification of bacterial burden in lung tissue and BALF.

**Figure 6:**
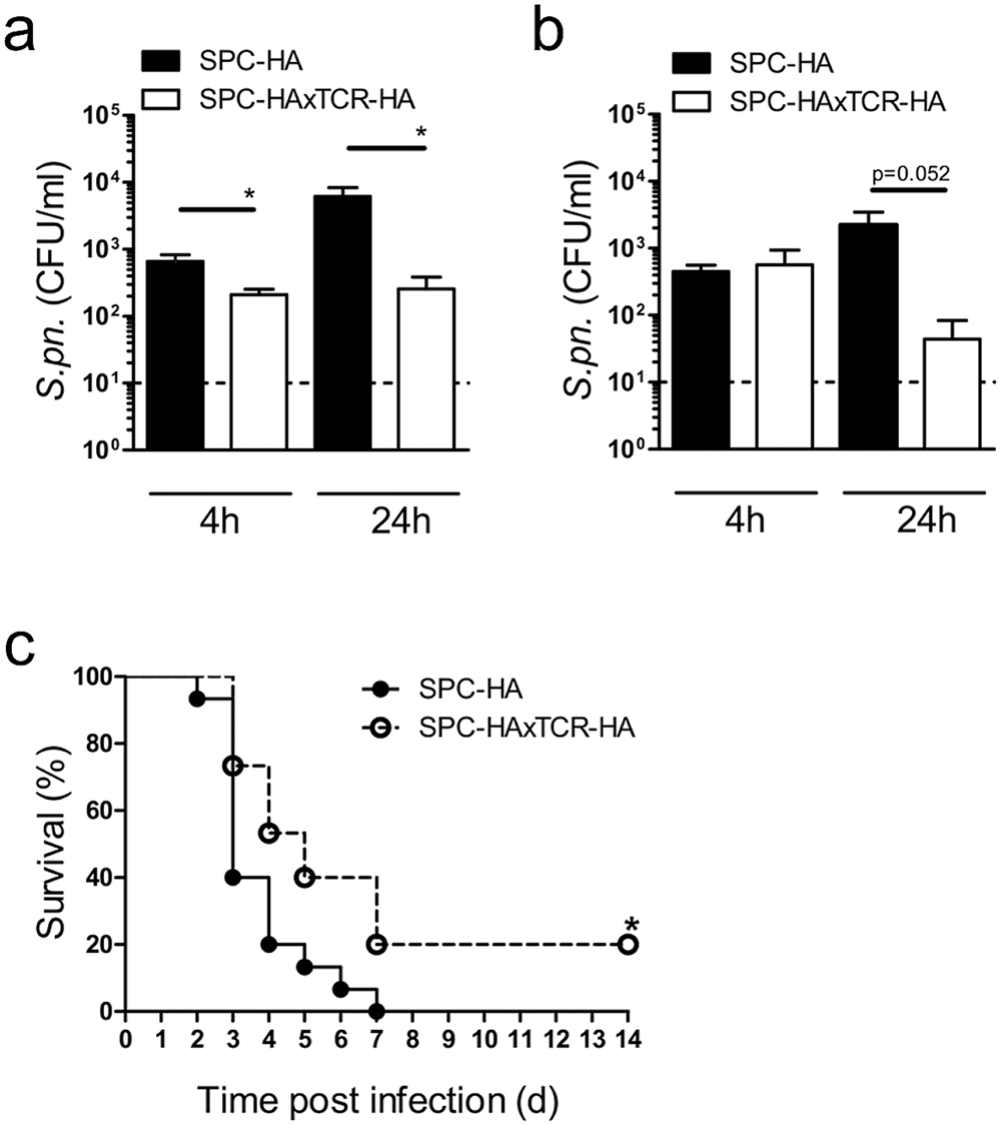
Improved antipneumococcal resistance in pre-diseased SPC-HAxTCR-HA mice. Mice were oropharyngeally inoculated with ~1.5 × 10^4^ CFU *S*. *pneumoniae* TIGR4 and were sacrificed at the indicated time points. The bacterial burden in bronchoalveolar lavage fluid (BALF) (**a**) and lung tissue homogenates (**b**) was determined. BALF and lung homogenates were taken from the same mice. Data were pooled from 2 independent experiments. Dashed lines indicate the detection limit. *p < 0.05. (**c**) Mice were oropharyngeally inoculated with ~1.5 × 10^6^ CFU *S*. *pneumoniae* TIGR4 and survival was followed over a period of 14 days. *p < 0.05 (n = 15/group, 2 independent experiments).

Already at 4 h post infection BALF of SPC-HAxTCR-HA mice contained significantly decreased bacterial numbers compared to SPC-HA control mice (Fig. 6a). Furthermore, the airways’ mean pneumococcal burden did not increase over the first 24 h following infection in SPC-HAxTCR-HA mice whereas there was an almost 9-fold increase of bacterial burden in SPC-HA mice (Fig. 6b). Consistent with reduced CFU in BALF, we found reduced proportions of lungs that were invaded by pneumococci in SPC-HAxTCR-HA mice (Table 2). Strikingly, following challenge with a lethal pneumococcal inoculum we observed 60% mortality within 3 days in SPC-HA mice compared to only 27% in SPC-HAxTCR-HA mice. Seven days post infection, all infected SPC-HA mice had succumbed whereas 20% of the SPC-HAxTCR-HA mice survived the otherwise lethal infection (Fig. 6c).

**Table 2:** Clearance of *S*. *pneumoniae* from the respiratory tract.

Hours after infection	BALF		Lung tissue	
	4	24	4	24
SPC-HA	0% (0/7)	25% (2/8)	13% (1/8)	38% (3/8)
SPC-HAxTCR-HA	0% (0/8)	44% (4/9)	50% (4/8)	78% (7/9)

SPC-HA and SPC-HAxTCR-HA mice were oropharyngeally inoculated with 1.5 × 10^4^ CFU *S*. *pneumoniae*. Depicted are percentages of pneumococci-free samples (cleared sample/total samples). Values correspond with data given in Fig. 6a and b.

Altogether, we correlate local, chronic inflammatory imprinting with marked changes in the transcriptomic and proteomic pulmonary profile. Our analyses consistently prove an enhancement of humoral immunity in inflamed lungs and suggested an involvement of the pulmonary epithelium therein. Finally, we could link increased pIgR-mediated SIg transepithelial transport to augmented pneumococcal binding by IgA and IgM coinciding with augmented host protection during pneumococcal pneumonia. These findings point towards a fundamental role of inflammation-primed airway epithelial cells in humoral antimicrobial immunity by reinforcing the mucosal barrier against airborne pathogens.

## Discussion

Several groups described beneficial effects of inflammatory imprinting in the lung on antimicrobial defense. Protection induced by mucosal administration of LPS^9^, mutant *E*.*coli* enterotoxin^22^ or nontypeable *Haemophilus influenzae* lysate^10, 23, 24^ involved both local and systemic increases in the pro-inflammatory cytokines IL-6 and TNF-α and intrapulmonary leukocyte recruitment. Inflammatory imprinting in SPC-HAxTCR-HA lungs is not triggered by PAMP receptor stimulation but rather includes a complex network of autoreactive and tolerogenic instruments^13–15^. Accordingly and in contrast to the above mentioned studies, local as well as systemic IL-6 and TNF-α levels were not detectable in naïve SPC-HA and SPC-HAxTCR-HA mice (data not shown). Nevertheless, gene expression and proteomic analysis of SPC-HAxTCR-HA lungs and BALF revealed a moderate auto-inflammatory response with only a small overlap between upregulated genes und unique proteins specific for SPC-HAxTCR-HA lungs. Thus, the substantial differences in protein composition between SPC-HA and SPC-HAxTCR-HA BALFomes are likely to reflect a combination of altered production rates by lung-resident cells, altered active transepithelial transport and passive transepithelial diffusion.

Importantly, we show that chronic inflammatory imprinting particularly favors the development of SIg-specific patterns in lung tissue as well as in the airway mucosal lining fluid of SPC-HAxTCR-HA mice involving strong pIgR protein expression. In lung histology pIgR-positive cells exhibited the typical cuboidal shape of type 2 pneumocytes and moreover, our unpublished gene expression data from type 2 pneumocytes purified by flow cytometry revealed that type 2 pneumocytes from SPC-HAxTCR-HA lungs indeed display increased *Pigr* expression compared to control SPC-HA type 2 cells (data not shown). Quantitative real-time RT-PCR from whole lungs shows only small increase (~3-fold) in *Pigr* expression in SPC-HAxTCR-HA vs. SPC-HA mice (Supplementary Fig. S1a). Generally, *Pigr* mRNA transcript is however steadily detectable as measured by standard end-point PCR in both genotypes (Supplementary Fig. S1b). Comparably, pIgR protein expression in SPC-HAxTCR-HA vs. SPC-HA mice is 64-fold elevated, indicating in summary that in the lung the pIgR protein is regulated on translational rather than transcriptional level. Similar observations have been made comparing *Pigr* expression in lungs of old vs. young mice showing 5-fold upregulation of pIgR protein despite an unchanged mRNA level^25^. However, the molecular stimuli leading to the induction of pIgR protein expression throughout the emergence of the SPC-HAxTCR-HA phenotype remain elusive.

An association of elevated epithelial pIgR expression and lung inflammation was also observed by others. It is known that pIgR is induced upon stimulation with various cytokines, hormones and by TLR stimulation^26^. Upon activation self-specific CD4^+^ T cells secrete IFN-γ^27^, a potent inducer of pIgR expression *in vitro* ^28^. In line with this, we found that T cell recognition of pulmonary self-antigen resulted in the production IFN-γ by autoreactive T cells^14^. Furthermore, transcriptomic analyses of autoreactive CD4^+^ T lymphocytes from SPC-HAxTCR-HA lungs previously revealed IL-17 gene induction in these cells^13^ and Il-17 signaling has formerly been shown by others to substantially enhance pIgR expression in airway^29^ and intestinal^30^ mucosal inflammation.

Several studies addressed the role of pIgR in the pathogenesis of bacterial disease. It was shown *in vitro* that the pneumococcal surface protein CbpA interacts with human pIgR^17^. Furthermore, CbpA revealed to be involved in pneumococcal transmigration of the murine blood-brain barrier^31^. Moreover, pneumococci co-localize with murine pIgR^32^. In contrast, Sun *et al*. described that abrogated mucosal SIgA transport in vaccinated pIgR knockout mice caused a loss of protection against nasopharyngeal pneumococcal colonization *in vivo* thus demonstrating the necessity of pIgR in airway antipneumococcal immunity^7^. One key reason for the abovementioned conflicting findings might be the restricted complexity of *in vitro* conditions, which poorly mimic the *in vivo* situation where increased pIgR expression inevitably leads to a reinforcement of mucosal surfaces by SIgs^29, 30^.

By using mice deficient for pIgR or its cargo molecules IgA and IgM, several groups have demonstrated a central role of the SIg axis in controlling airway pneumococcal burden and tissue invasion^4, 7^. Importantly, recurrent respiratory infections are comorbidities associated with selective IgM or IgA deficiencies in humans^33, 34^. We hypothesize that enhanced antipneumococcal resistance in SPC-HAxTCR-HA mice can originate from increased airway mucosal SIgA or SIgM concentrations. Since pIgR-mediated transcytosis of immunoglobulins follows a 1:1 stoichiometry, i.e. requiring one pIgR-protein and one Ig-protein to produce one SIg-complex^35–37^, the higher abundance of the pIgR-protein (Fig. 3) in the inflamed lung very well explains the elevated SIgA concentration in the BAL of SPC-HAxTCR-HA mice. Accordingly, we detected significantly increased IgA and IgM coverage of pneumococcal surfaces as well as increased portions of IgM^+^ pneumococci using SPC-HAxTCR-HA BALF (Fig. 4). As this study was conducted using naïve mice, one likely target epitope recognized by the polyreactive, natural SIg pool is the phosphocholine of the pneumococcal cell wall C-carbohydrate^38^. Altogether these data demonstrate that inflammatory respiratory priming favors the induction of epithelial pIgR expression which ultimately results in increased pulmonary levels of IgA and IgM and enhanced pneumococcal immune exclusion.

With regard to other immunologic mechanisms protective to pneumococcal infection such as complement-dependent bacterial killing and chemotactic phagocyte attraction, we could not find indication for enhanced C5 activation or macrophage and neutrophil recruitment in SPC-HAxTCR-HA mice (Fig. 5). We have previously shown that AMs in SPC-HAxTCR-HA lungs show markedly decreased TNF-α immune responses towards *S*. *pneumoniae* TIGR4. This was furthermore associated with a transcriptional M2 signature of these cells in SPC-HAxTCR-HA lungs^39^. TNF-α is involved in macrophage-dependent cytotoxicity^40^ as well as intrapulmonary neutrophil recruitment^41^ and has been shown to be essential for antipneumococcal host defense in the lung^42^. Although we cannot formally exclude the relevance of other macrophage-dependent mechanisms, e.g. such as opsonin-independent phagocytosis, we hypothesize that the reduced inflammatory response of these cells in SPC-HAxTCR-HA mice in principle is disadvantageous during respiratory pneumococcal infection. This in turn underpins the immunological relevance of other antimicrobial mechanisms e.g. epithelial-driven reinforcement of the airway mucosa in the pre-diseased lung.

We and others have previously described the key role of airway epithelial cells (AECs)^11, 23, 43^ in inducible host defense against pathogens and AECs are potent producers of APs^2^. Our proteomic approach detected pulmonary surfactant-associated protein A1, cathelin-related antimicrobial peptide and lactotransferrin uniquely in the SPC-HAxTCR-HA BALFome, thus confirming a participation of microbicide mechanisms in the pulmonary response towards inflammatory priming. Still, our analyses are also unable to track the cellular sources of APs. Lactotransferrin binds to *S*. *pneumoniae* ^44^, however its antipneumococcal activity has been questioned^45^ and binding to lactotransferrin has been suggested as a pneumococcal iron-supply mechanism^44^. *In vitro* pneumococcal killing assays using diluted BALF from SPC-HAxTCR-HA and SPC-HA mice did not uncover potential differences in AP-mediated bactericidal activity between the groups (Supplementary Fig. S6). Although we still do not completely exclude the *in vivo* contribution of APs in limiting bacterial outgrowth in the airways at 4 and 24 hours post infection (Fig. 6a,b) we suggest that enhanced coverage of pneumococci by SIgA/SIgM in the inflamed airways (Fig. 4) more efficiently prevents the bacteria from attaching to and invading of the epithelial layer. This is well in line with the observation that 24 h following low-dose infection SPC-HAxTCR-HA mice by trend exhibit a decreased bacterial burden in the lung tissue (Fig. 6b) and moreover, clearly more lungs of SPC-HAxTCR-HA mice are entirely free of bacteria 4 h (50% SPC-HAxTCR-HA vs 13% SPC-HA) and 24 h (78% SPC-HAxTCR-HA vs 38% SPC-HA) following infection, respectively (Table 2). In the same line, upon high-dose pneumococcal infection survival is significantly prolonged in SPC-HAxTCR-HA mice (Fig. 6c) suggesting delayed bacterial invasion and sepsis development in mice with preexisting lung inflammation, most likely involving improved SIgA/SIgM-mediated immune exclusion.

It is widely recognized that bacterial pathogens (in particular pneumococci) play a role in stable disease as well as during acute exacerbations of COPD. Moreover, with increasing disease severity the risk of complications due to pulmonary infections also increases^46^. To our knowledge, there is however no true consensus about the mechanisms that decrease antibacterial defense during COPD deterioration. In this context, Ohlmeier and colleagues reported elevated pIgR levels in sputum, alveolar and bronchial epithelium of smokers and individuals with mild-to-moderate COPD^47^. While this study did not address the effect of enhanced pIgR expression on pathogen-specific immunity, data on inflammation-associated pIgR overexpression are well in line with our findings in SPC-HAxTCR-HA mice which as well exhibit mild-to-modarate chronic lung inflammation. In contrast to early-stage COPD, proceeding bronchial epithelial remodeling in COPD patients has been shown to be associated with a progressive loss of bronchial epithelial pIgR expression ultimately resulting in localized SIgA deficiency in airways of advanced COPD lungs^48^ which was mechanistically linked to TGFβ1^49^. Indeed, a direct correlation between epithelial surface SIgA and severity of airflow obstruction as measured by FEV1 as an indicator for COPD severity was demonstrated^48^. Given the crucial importance of pIgR and its cargo molecules IgA and IgM to antibacterial defense^4, 7, 8^ it appears obvious that reduced pIgR expression secondary to altered epithelial cell differentiation should be considered a major cause of impaired mucosal immunity to respiratory pathogens in patients with advanced COPD. Recently it was shown that pIgR^−/−^ mice lacking SIgA in their airways spontaneously develop COPD-like pathology at age. Strikingy, emphysema formation was driven by resident airway microbiota that in absence of the mucosal SIgA barrier could directly attach to and invade the airway epithelium, thereby inducing an inflammatory cascade culminating in progressive tissue remodeling^50^. The intriguing finding that upon SIgA-deficiency airway microbiota directly contribute to the pathogenesis of lung inflammation together with the aforementioned advancing loss of SIgA during disease progression in the airways of COPD patients directly suggest a so far underappreciated role of the pIgR/SIgA axis and related SIg-mediated immune exclusion of not only pathogens but as well commensals in the pathophysiology of CRD. In turn these data as well suggest improved immune exclusion of bacteria under conditions were pIgR is overexpressed thereby resulting in elevated SIgA and SIgM levels in the airways. This is exactly what we observe in chronic lung inflammation in SPC-HAxTCR-HA mice. The evidence of pIgR/Ig mechanisms as effectors of improved respiratory immunity is of interest for future investigations. Despite the findings of unaltered bacterial binding by AMs in our model, patients with severe asthma or COPD are frequently diagnosed with impaired AM capacity^51, 52^ revealing insufficient myeloid-dependent host defense while at the same time underlining the key role of humoral immunity in ensuring broad protection against airborne pathogens. Further studies are needed to explore whether prophylactic pIgR enhancement serves as a therapeutic strategy for CRD patients with recurrent pneumococcal infections.

## Methods

### Mice

TCR-HA, SPC-HA and SPC-HAxTCR-HA mice are on BALB/c genetic background and have been described previously^13, 53^. Mice were bred and housed in the animal facility at the Helmholtz Centre for Infection Research in SPF conditions according to the national and institutional guidelines and were used at 12–24 weeks of age. All experiments were approved by the local government agency (Niedersächsisches Landesamt für Verbraucherschutz und Lebensmittelsicherheit; file number: 33.19-42502-04-14/1715) and have been performed in accordance to these guidelines.

### Pneumococcal infections

For infections an encapsulated serotype 4 strain (TIGR4) was used. Preparation of pneumococcal inoculums, mouse infections and monitoring were performed as previously described^54^. For determination of CFU bronchoalveolar lavage (BAL) was performed by flushing the lungs with 1 ml PBS. To quantify the rate of pneumococcal invasion lungs were perfused with ~10 mL PBS, aseptically removed and homogenized in 1 mL PBS. Serial dilutions of BALF and lung tissue homogenates were plated onto blood agar plates, incubated overnight and CFU counts of individual compartments were calculated.

### Analyses of macrophage bacterial binding

Bacterial inoculums of *S*. *pneumoniae* were stained in 5 µM Cell Proliferation Dye eFluor® 670 (eBioscience) and resuspended in PBS. Mice were oropharyngeally administered with 0.5–1 × 10^7^ CFU in 50 µL, sacrificed 90 min post administration and BAL was performed twice with a total volume of 1.5 mL PBS. Obtained cells were erythrocyte-depleted by treatment with ACK buffer, Fcγ-receptors were blocked with anti-CD16/CD32 (clone: 93); cells were stained with anti-F4/80 (clone: BM8, both BioLegend) and binding was analyzed using a LSR Fortessa flow cytometer (BD Biosciences, San Jose, CA). Alveolar macrophages (AMs) were identified as F4/80+, autofluorescence^high^ and side scatter^high^ cells. Relative binding capacity of each sample was determined by calculating: median fluorescence intensity (MFI) of e670-positive AMs over the mean of all MFIs of e670-positive AMs of the control group (SPC-HA mice).

### Single-cell preparation and flow cytometric analyses

BALF cells and perfused lung tissue were obtained as described above. Lungs were manually minced and enzymatically digested (37 °C, 45 min) in Iscove’s modified Dulbecco’s medium (IMDM) with GlutaMAX-1 (Life Technologies, Germany) supplemented with 0.2 mg/ml collagenase D (Roche Diagnostics, Germany), 1 mg/ml DNase (Sigma-Aldrich, Germany) and 5% fetal bovine serum (FBS Forte; Pan Biotech, Germany). Reaction was stopped by the addition of 5 mM EDTA (working concentration). Cell suspension was filtered (100 µm), pelleted (420 × g, 4 °C), erythrocyte-depleted using ACK buffer and leukocytes were purified using 35% Easycoll Separating Solution (Density: 1.124 g/mL, Biochrom, Germany). BALF cells and lung tissue single-cell suspensions were incubated in a mixture of the Live/Dead fixable blue stain (ThermoFisher, USA) for dead-cell exclusion and anti-CD16/CD32 antibody for Fc receptor blocking. Cell surface staining was then performed using antibodies specific for mouse CD11c (clone: N418, BioLegend, USA), F4/80 (clone BM8,BioLegend, USA), CD11b (clone M1/70, BioLegend, USA), and Ly6G (clone: 1A8; BioLegend, USA). Alveolar macrophages (AMs; CD11c+ CD11b− F4/80+ autofluorescence+) and neutrophils (CD11c− CD11b+ Ly6G+ F4/80−) were identified through gating on the respective populations. Cellular counts in the respective organ/fluid were determined by adding a defined number of counting beads prior staining to each sample and using the individual bead loss factor as a measure to calculate the actual cell counts in the respective sample.

### Proteomic analyses

Proteins were purified by chloroform/methanol precipitation^55^. Protein pellets from each sample were reduced (10 mM DTT), alkylated (20 mM jodoacetamide) and digested with sequencing grade modified trypsin (Promega, Madison, WI) as recommended at 37 °C overnight. Peptide solutions were vacuum-dried, peptides were resolubilized in 0.2% TFA, desalted on self-packed LiChroprep RP-18 (Merck, Darmstadt, Germany) SPE columns, eluted with 0.2% TFA in 60% ACN and vacuum-dried. LC-MS/MS analyses of desalted samples were performed on an Acquity ultraperformance LC system (Waters Corp., Milford, MA) connected to a LTQ Orbitrap XL mass spectrometer (Thermo Finnigan Corp., San Jose, CA). Peptides were flushed onto a C18 precolumn (5-μm Symmetry C18, 180 μm × 20 mm, Waters Corp.) with a flow rate of 15 μl/min and washed at constant flow for 3 min. Peptides were separated on an analytical column (1.7-μm BEH130, 75 μm × 150 mm, Waters Corp.) with UPLC buffer A (0.1% formic acid in water) and UPLC buffer B (0.1% formic acid in acetonitrile) via linear 120-min gradients at a flow rate of 300 nl/min controlled with Acquity UPLC software V1.22. Eluting peptides were ionized using PicoTip emitter needles (New Objective Inc., Woburn, MA), voltages of 1.7 kV and a capillary temperature of 200 °C. Data-dependent acquisition of MS and MS/MS data was under control of XCalibur software V2.1 (Thermo Finnigan). Survey Scans were acquired in the Orbitrap mass analyzer with a resolution of 60.000 at m/z 400. For full scans 5 × 10^5^ ions were accumulated within a maximum injection time of 500ms and detected in the Orbitrap analyzer. Sequential isolation of the five most intense ions with charge states ≥2 was set to a target value of 5 × 10^4^ (signal threshold: 2 × 10^4^, isolation width: 4.0 Da) with a maximum injection time of 500 ms. CID mode was used for detection of produced fragment ions. Database searches were performed using Proteome Discoverer (1.4; Thermofisher) connected to a Mascot Server (2.4.06) in the UniProtKB/Swiss-Prot database (2015_01 taxonomy, Mus musculus with 16,702 sequences). Proteins were accepted as identified when at least one unique, search engine rank 1 and high confident peptide showed an individual score above 30, which indicates identity or extensive homology (p < 0.05) based on the search parameter settings used (enzyme, trypsin; maximum missed cleavages, 1; fixed modification: carbamidomethylation (Cys); variable modifications, phosphorylation (Ser, Thr, Tyr) and oxidation (Met); peptide tolerance, 10 ppm; MS/MS tolerance, 0.4 Da).

### Microarray analyses

RNA from whole lung tissue was isolated and purified using RNeasy Midi and RNase free DNase kit (both Qiagen, Hilden, Germany). Samples were amplified, labeled, fragmented and hybridized to GeneChip® Mouse Gene 1.0 ST arrays (Affymetrix) and treated according to manufacturer’s instructions. Microarray scanning was performed using an Affymetrix GCS 3000 scanner and GCOSv1.1 software. Data analyses were conducted using GeneSpring GX software (Agilent technologies). The data were summarized, log2 transformed and normalized with the Robust Multi-array Analysis (RMA) algorithm. Cluster and ANOVA analyses were performed with Genesis software 1.7.6, applying a z-score transformation. Gene-ontology analyses were performed using Cytoscape software 3.2.0 and ClueGo plugin^56^. Gene Set Enrichment Analysis (GSEA) was performed with GSEA software tool and hallmark gene sets of the Molecular Signature Database^57^.

### ELISA

ELISA kits for mouse IgA and IgM (eBioscience, Frankfurt am Main, Germany), mouse IL-6 and TNF-α (BioLegend, London, United Kingdom), mouse C5a (R&D Systems, Minneapolis, MN) and mouse albumin (Bethyl Laboratories Inc., Montogomery, TX) were used according to manufacturer’s instructions. For SIgA-detection Nunc MaxiSorp plates were coated overnight with monoclonal rat anti-mouse IgA (SouthernBiotech, Birmingham, 2 µg/mL). Plates were washed, blocked and incubated with serial dilutions of BALF samples and incubated with secondary antibody (polyclonal goat anti-mouse pIgR; R&D Systems, 1 µg/mL) followed by polyclonal donkey anti-goat-HRP (Jackson ImmunoResearch, West Grove, PA, 0.16 µg/mL) and TMB substrate. Plates were read at 450 and 570 nm. Due to the lack of a SIgA protein standard, relative quantification was performed. Results are expressed as the OD at 450 nm (wavelength- and blank-corrected) at indicated dilutions.

### qPCR

RNA from whole lung homogenates was transcribed into cDNA using M-MLV RT polymerase (Invitrogen, Carlsbad, CA) according to manufacturer’s instructions. Quantitative PCR (qPCR) was conducted using the LightCycler 480 SYBR Green I Mastermix and LightCycler 480 instrument (both Roche, Mannheim, Germany). Primer sequences are shown in Supplementary Table S8. Transcript levels were normalized to the housekeeping gene *Actb*.

### Immunoblot

Lungs were homogenized in 5 mL ice-cold RIPA lysis buffer (25 mM TrisHCl pH 7.6, 150 mM NaCl, 1% IGEPAL CA-630, 1% sodium deoxycholate, 0.1% SDS). Cell debris was removed by centrifugation (15 min, 5250 × g, 4 °C) and supernatants were stored at −70 °C. Protein concentration was measured with BCA Protein Assay Kit (Thermo Fisher Scientific, Waltham, MA). Equal amounts of protein were separated by SDS-PAGE (8%) and transferred to a PVDF membrane. Target was identified using anti-pIgR antibody, reference protein was identified using anti-GAPDH (Cell Signaling Technology, Inc., Beverly, MA). For detection standard horseradish peroxidase conjugated polyclonal secondary antibodies were used together with ECL western blotting substrate (Life Technologies, Darmstadt, Germany). Protein bands were densitometrically quantified using ImageJ software (version 1.50i).

### Histology

For pIgR localization lungs were frozen in Tissue-Tek O.C.T. (Sakura Finetek GmbH, Staufen, Germany). 6 µM acetone-fixed cryosections were blocked with anti-CD16/CD32, incubated with goat anti-pIgR primary antibody, blocked with 5% donkey serum and incubated with FITC-conjugated donkey anti-goat secondary antibody (Jackson ImmunoResearch, West Grove, PA). Alveoli were identified by DAPI nuclear counterstain. Fixed tissue sections were analyzed by fluorescence microscopy, using the inverted Eclipse Ti-U, NIS-Elements imaging software (Nikon, Tokyo, Japan) and ImageJ software.

### Opsonization

BAL was performed using 1 mL PBS. Cells were separated by centrifugation (420 × g, 10 min, 4 °C) and BAL supernatants were stored at −70 °C. 96-well microplates were incubated with blocking buffer (1% PBS/BSA). Freshly grown pneumococci were washed twice with blocking buffer, seeded into microwells (1–4 × 10^6^ CFU) and incubated (45 min, 4 °C) with BAL supernatants. Bacteria were washed once with PBS and stained using anti-mouse IgA (clone: C10-3) or anti-mouse IgM (clone: DS-1, both BD Pharmingen). Opsonization was quantified by flow cytometry.

### Statistical analyses

Data are expressed as mean ± SEM. Statistical analyses were performed by unpaired, two-tailed Mann-Whitney test. Comparison of >2 groups was done using Kruskal-Wallis test and Dunns Post Test. Survival data was analyzed by Log-rank (Mantel-Cox) test using GraphPad Prism software (La Jolla, CA).

### Data availability

All data generated or analysed during this study are included in this published article (and its Supplementary Information files). Microarray data are deposited at Gene Expression Omnibus (http://www.ncbi.nlm.nih.gov/geo/), accession ID: GSE66531).

## Electronic supplementary material

Supplementary Information
