# Supplementary material for: Chronic lung inflammation primes humoral immunity and augments antipneumococcal resistance
Source: Sci Rep. 2017 Jul 10;7:4972. doi: 10.1038/s41598-017-05212-4 (PMC5504016; doi:10.1038/s41598-017-05212-4)
Supplement: Supplementary file 1 — Supplementary Information [file 41598_2017_5212_MOESM1_ESM.doc]

**Chronic lung inflammation primes humoral immunity and augments antipneumococcal resistance**

Julia D. Boehme^1,2^, Sabine Stegemann-Koniszewski^2^, Andrea Autengruber^2^, Nicole Peters^1^, Josef Wissing^3^, Lothar Jänsch^3^, Andreas Jeron^1,2#^, Dunja Bruder^1,2#*^

^1^Infection Immunology Group, Institute of Medical Microbiology and Hospital Hygiene, Otto-von-Guericke University, Magdeburg, Germany ^2^Immune Regulation Group, Helmholtz Centre for Infection Research, Braunschweig, Germany ^3^Cellular Proteomics Group, Helmholtz Centre for Infection Research, Braunschweig, Germany

# These authors contributed equally to this work.

*** Corresponding author:** Dunja Bruder, Institute of Medical Microbiology, Otto-von-Guericke University, Leipziger Straße 44, 39120 Magdeburg, GERMANY, Tel.: +49(0)391-67-13374 Fax: +49(0)391-67-13384, e-mail: dunja.bruder@med.ovgu.de

**Supplementary information**

**Supplementary figures**


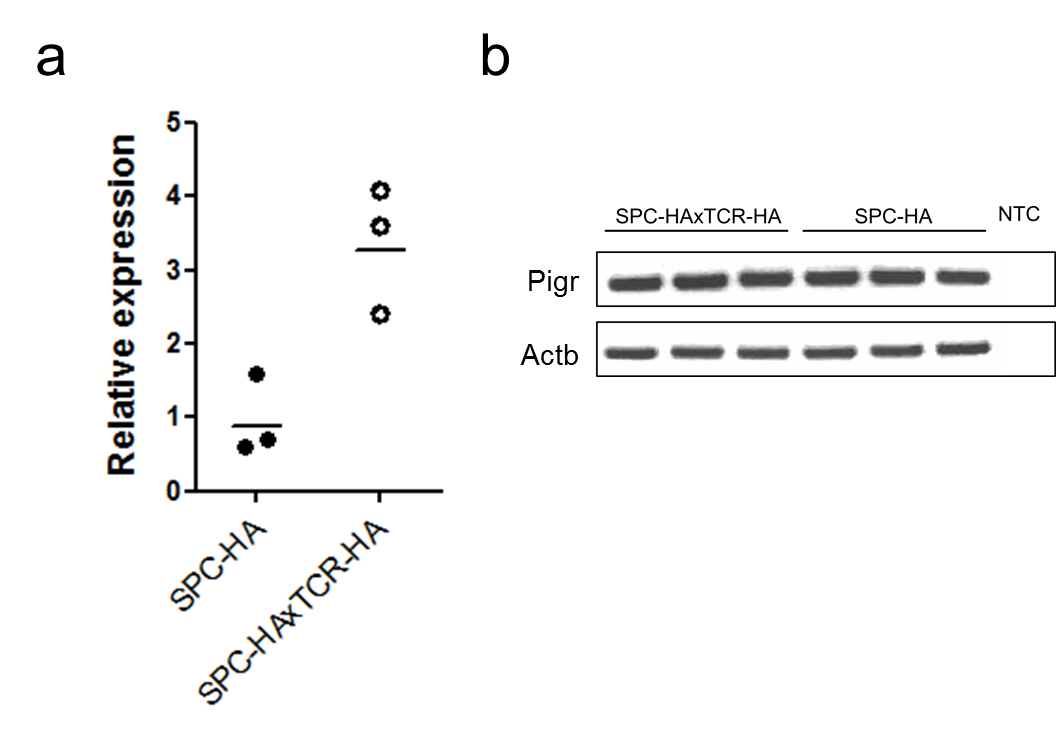


**Figure S1:** **Lung tissue *Pigr* gene expression.** (a) *Pigr* transcript levels were determined by quantitative real-time RT-PCR (qPCR). Results were normalized to beta actin (*Actb)* transcript levels and are expressed as relative expression (compared to the SPC-HA control group). (b) PCR reactions were analyzed on 1% agarose gel. NTC: no template control.


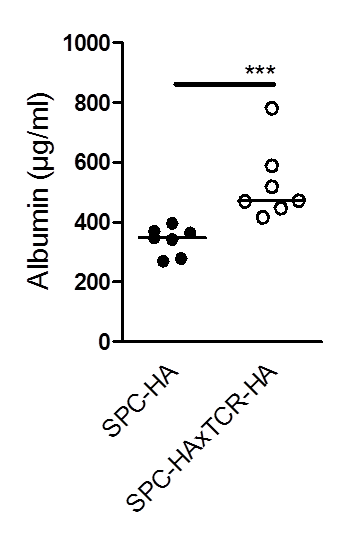


**Figure S2:** **Serum albumin concentration in the airways.** Albumin concentration in bronchoalveolar lavage fluid (BALF) of naïve mice was measured by ELISA ***p<0.001

**
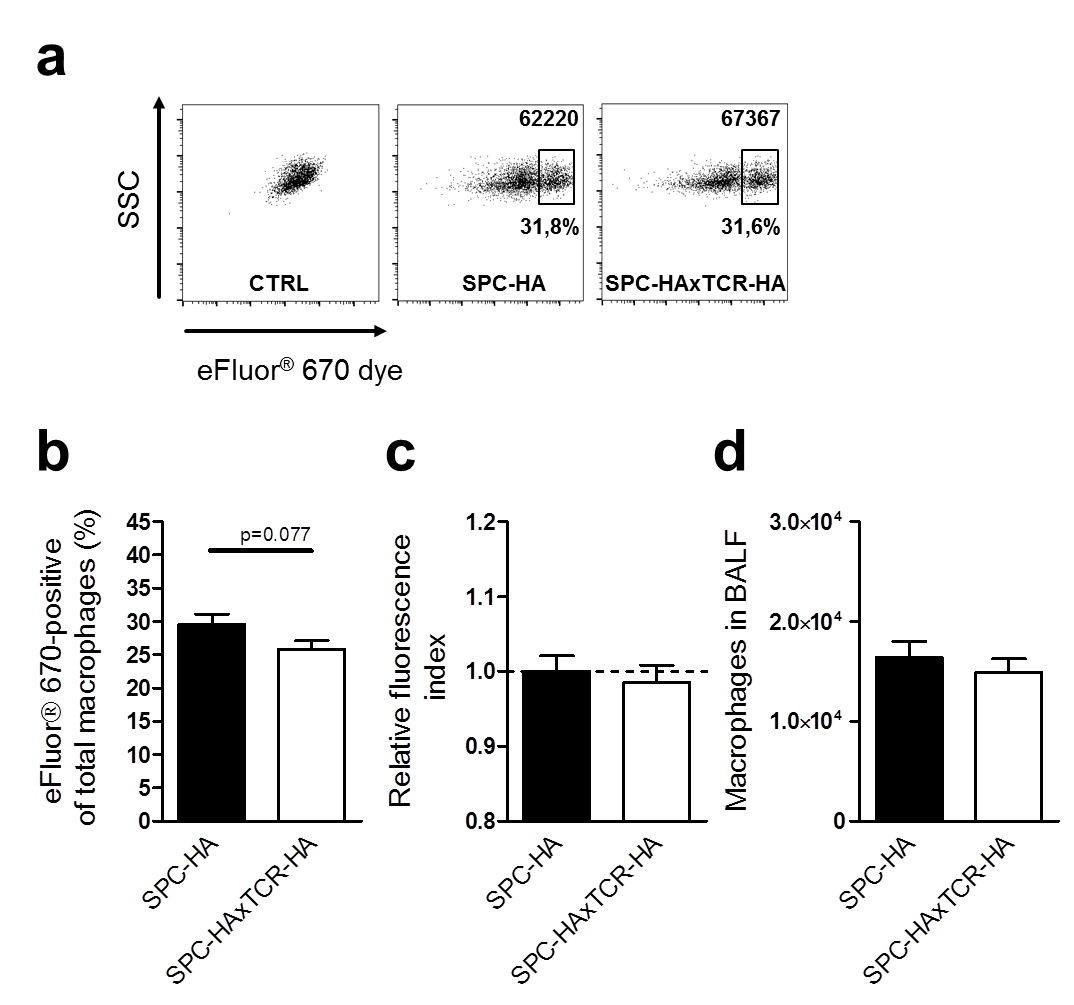
**

**Figure S3:** **Similar bacterial binding capacities of SPC-HA and SPC-HAxTCR-HA alveolar macrophages.** Mice were oropharyngeally inoculated with ~5x10^6^ CFU eFluor®670-stained *S.pneumoniae* TIGR4. Bronchoalveolar lavage cells were isolated 90 min post infection and analyzed by flow cytometry. (a) Representative FACS plots of eFluor®670-positive macrophages; macrophages from non-infected BAL samples were used as controls (CTRL). Pictures depict the portions of pneumococci-positive macrophages out of all recovered macrophages and the median fluorescence intensity (MFI, upper right corner). (b) Portions of eFluor®670+ alveolar macrophages out of all recovered alveolar macrophages. (**c**) The relative fluorescence index of eFluor®670+ macrophages of each mouse is determined by the ratio of the MFI of e670+ macrophages over the mean MFI of the SPC-HA control group samples. (**d**) Absolute macrophage numbers in BALF were determined by cell counting and flow cytometric analyses. Data are pooled from at least 2 independent experiments with similar results.

Figure S4: Increased numbers of T and B lymphocytes in the airways of SPC-HAxTCR-HA mice. Airway leukocytes were obtained from bronchoalveolar lavage fluid (BALF) of naïve SPC-HA ( n = 5) and SPC-HAxTCR-HA mice (n =6). Information on true cell counts of the indicated leukocyte subsets were obtained by flow cytometric analyses and cell counting; AMs (F4/80^+^, autofluorescence^high^, side scatter^high^), T cells (CD3^+^, Nkp46^-^), B cells (CD19^+^), neutrophils (CD11b^+^, Ly6G^+^), NK cells (CD3^-^, CD19^-^, Nkp46^+^). Data represent mean ± SEM. **p<0.01 in Mann-Whitney test.


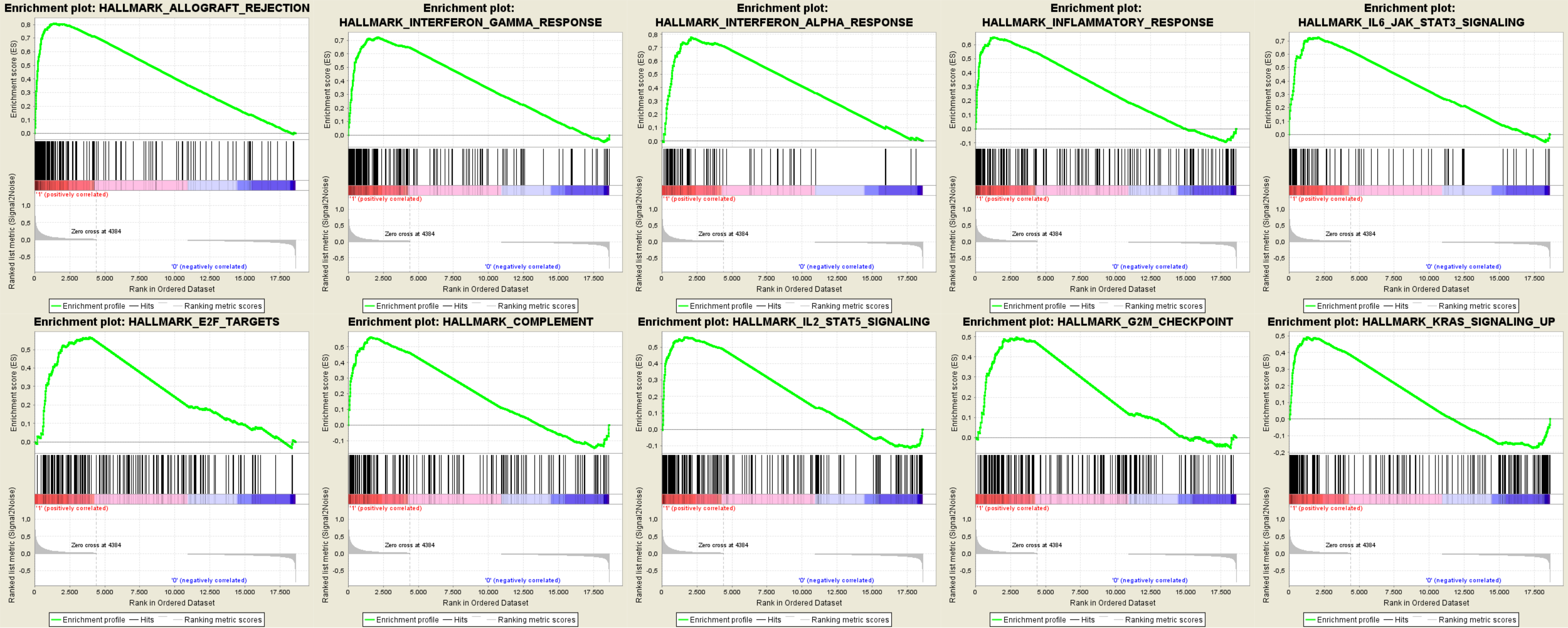


**Figure S5:** **Gene Set Enrichment Analysis comparing SPC-HAxTCR-HA vs. SPC-HA.** Whole lung microarray data from SPC-HAxTCR-HA and SPC-HA mice were analyzed for gene set enrichment for canonical hallmark gene sets from the Molecular Signature Database (MSigDB). Results show gene sets with a FDR < 5% matching supplementary table S4.


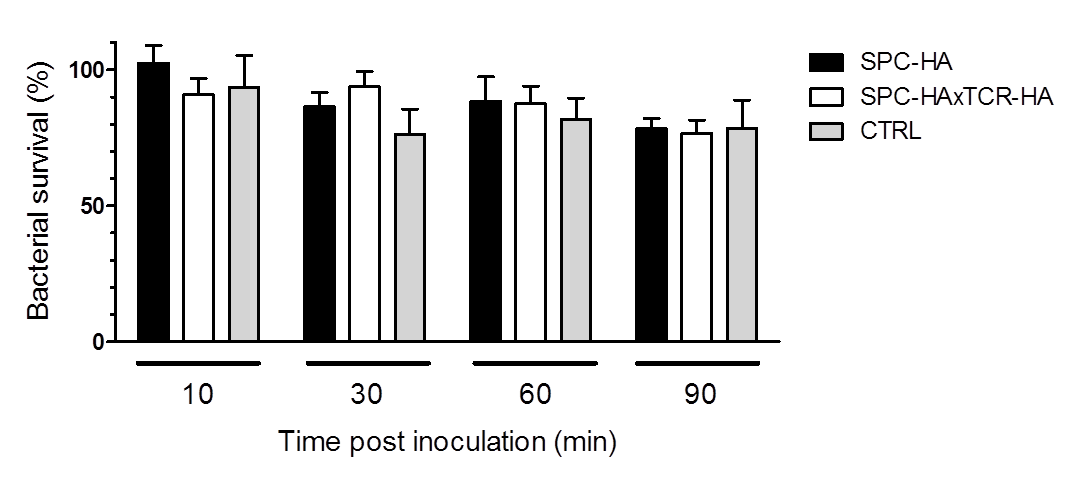


Figure S6: No antimicrobial activity of BALF from SPC-HA or SPC-HAxTCR-HA mice. BALF supernatants from naive SPC-HA or SPC-HAxTCR-HA mice were inoculated with *S. pneumoniae* TIGR4 at 4°C. After indicated time points bacterial survival was quantified by plating serial dilutions of the BALF/S.pn. samples on blood agar plates. Bacterial survival was determined by the ratio of recovered bacteria at the indicated time points in crelation to the initial bacterial concentration. Control PBS/S.pn. samples (n=4) were utilized to monitor bacterial viability over time.

(n=9 BALF samples/group, 2 independent experiments

**Supplementary tables**

**Table S1:** List of top20 up-regulated genes in SPC-HAxTCR-HA lungs.

| **Gene symbol** | **SPC-HA**  **[log2 SI ±SD]** | **SPC-HAxTCR-HA**  **[log2 SI ±SD]** | **FC** | **p-value**  **one-way ANOVA** |
| --- | --- | --- | --- | --- |
| **Clca3** | 6,8 ± 0,2 | 11,7 ± 0,2 | 29,4 | 4.74E-06 |
| **Glycam1** | 6,6 ± 0,2 | 11,1 ± 0,3 | 23,1 | 4.14E-05 |
| **Gm5571** | 7,6 ± 0,5 | 11,9 ± 0,1 | 19,3 | 1.37E-04 |
| **Igk-V1** | 6,1 ± 0,6 | 9,9 ± 0,6 | 14,2 | 1.57E-03 |
| **LOC672291** | 7,3 ± 0,5 | 11,1 ± 0,5 | 14,2 | 7.29E-04 |
| **Igj** | 7,5 ± 0,2 | 11,2 ± 0,2 | 13,6 | 2.94E-05 |
| **Cxcl9** | 7,3 ± 0,1 | 10,9 ± 0,4 | 12 | 6.56E-05 |
| **LOC672291|Igk-J1** | 7,5 ± 0,5 | 11,1 ± 0,3 | 11,8 | 5.11E-04 |
| **Igk-V19-14** | 7,5 ± 0,4 | 11,1 ± 0,8 | 11,6 | 2.10E-03 |
| **Gm4964** | 7,3 ± 0,2 | 10,6 ± 0,1 | 9,6 | 1.39E-05 |
| **Gm1502|Gm8760|Igkv4-71** | 7,4 ± 0,4 | 10,6 ± 0,3 | 9,4 | 3.85E-04 |
| **LOC100046496** | 7,2 ± 0,2 | 10,4 ± 0,8 | 8,8 | 2.51E-03 |
| **Igl-V1|Igl-V2|LOC433053** | 8,5 ± 0,5 | 11,7 ± 0,3 | 8,7 | 7.70E-04 |
| **Ear11** | 6,0 ± 0,1 | 9,1 ± 0,8 | 8,6 | 2.25E-03 |
| **Igkv4-71|Gm8760|Gm1499** | 8,5 ± 0,2 | 11,6 ± 0,1 | 8,6 | 3.16E-05 |
| **Dnase1l3** | 7,0 ± 0,2 | 10,1 ± 0,5 | 8,5 | 6.74E-04 |
| **Gm16848** | 6,7 ± 0,3 | 9,7 ± 0,8 | 8,1 | 2.81E-03 |
| **Igl-V2|Igl-C2** | 7,4 ± 0,1 | 10,4 ± 0,3 | 8 | 8.03E-05 |
| **Gm10880** | 8,0 ± 0,3 | 11,0 ± 0,1 | 7,8 | 6.12E-05 |
| **Gm16970|Igh-VX24** | 8,1 ± 0,2 | 11,1 ± 0,1 | 7,7 | 1.30E-05 |

Depicted are mean normalized log2 transformed signal intensities (SI) ± standard deviation (SD), the calculated fold change (FC) of SPC-HAxTCR-HA vs. SPC-HA and the p-value of one-way ANOVA analysis.

**Table S2:** Table of 285 significantly regulated transcripts SPC-HAxTCR-HA vs. SPC-HA lungs. S = SPC-HA; SxT = SPC-HAxTCR-HA. Columns named “S” and “SxT” contain normalized log_2_ signal intensities for each microarray replicate (n = 3/group). Fold change was calculated based on average SPC-HAxTCR-HA signal intensity vs. average SPC-HA signal intensity. Table is sorted first according to k-means cluster assignment (as shown in Figure 1) and secondarily within each cluster descending according to their fold change (high to low). Positive fold changes are indicated in red, negative fold changes are in green.

| **Transcript Cluster ID** | **Gene Symbol** | **Gene description** | **k-means cluster** | **S 1** | **S 2** | **S 3** | **SxT 1** | **SxT 2** | **SxT 3** | **Fold change (SxT**  **vs. S)** | **one-way ANOVA p-value** |
| --- | --- | --- | --- | --- | --- | --- | --- | --- | --- | --- | --- |
| **10545249** |  |  | Cluster 1 | 6.1 | 5.9 | 5.7 | 8.3 | 9.0 | 11.6 | 13.4 | 2.09E-02 |
| **10545247** | *Igk-V19-14* | immunoglobulin kappa chain variable 19 (V19)-14 | Cluster 1 | 8.0 | 7.3 | 7.3 | 10.4 | 10.9 | 11.9 | 11.6 | 2.10E-03 |
| **10538924** | *LOC100046496* | similar to Ig kappa V-region 24B | Cluster 1 | 7.4 | 7.0 | 7.3 | 9.5 | 11.0 | 10.6 | 8.8 | 2.51E-03 |
| **10417526** | *Dnase1l3* | deoxyribonuclease 1-like 3 | Cluster 1 | 7.2 | 7.0 | 6.7 | 9.6 | 10.0 | 10.6 | 8.5 | 6.74E-04 |
| **10545220** | *Gm16848* | predicted gene, 16848 | Cluster 1 | 6.8 | 6.4 | 6.9 | 9.2 | 9.4 | 10.6 | 8.1 | 2.81E-03 |
| **10562812** | *Spib* | Spi-B transcription factor (Spi-1/PU.1 related) | Cluster 1 | 7.7 | 7.4 | 7.5 | 9.6 | 10.3 | 10.9 | 6.7 | 2.10E-03 |
| **10403018** | *IghmAC38.205.12|AI324046* | Ig mu chain V region AC38 205.12 | expressed sequence AI324046 | Cluster 1 | 8.0 | 8.2 | 7.7 | 10.1 | 10.5 | 11.1 | 6.0 | 1.32E-03 |
| **10551025** | *Cd79a* | CD79A antigen (immunoglobulin-associated alpha) | Cluster 1 | 8.4 | 8.3 | 7.8 | 10.2 | 10.6 | 11.2 | 5.7 | 1.92E-03 |
| **10545180** | *Gm10879* | predicted gene 10879 | Cluster 1 | 7.0 | 6.9 | 6.2 | 8.6 | 9.6 | 9.5 | 5.6 | 3.35E-03 |
| **10361292** | *Cr2* | complement receptor 2 | Cluster 1 | 6.7 | 6.8 | 6.6 | 8.6 | 9.3 | 9.7 | 5.6 | 1.57E-03 |
| **10402981** | *Gm900* | predicted gene 900 | Cluster 1 | 6.7 | 7.0 | 6.7 | 8.6 | 9.7 | 9.6 | 5.5 | 2.38E-03 |
| **10576757** | *Fcer2a* | Fc receptor, IgE, low affinity II, alpha polypeptide | Cluster 1 | 7.4 | 7.2 | 7.3 | 9.1 | 9.5 | 10.2 | 5.0 | 2.14E-03 |
| **10512470** | *Cd72* | CD72 antigen | Cluster 1 | 8.3 | 8.2 | 8.1 | 10.1 | 10.4 | 11.0 | 4.9 | 1.05E-03 |
| **10509577** | *Pla2g2d* | phospholipase A2, group IID | Cluster 1 | 8.8 | 8.7 | 8.6 | 10.6 | 10.9 | 11.4 | 4.8 | 7.05E-04 |
| **10349593** | *Faim3* | Fas apoptotic inhibitory molecule 3 | Cluster 1 | 8.1 | 7.9 | 8.0 | 9.7 | 10.3 | 10.8 | 4.8 | 2.18E-03 |
| **10450880** | *H2-M2* | histocompatibility 2, M region locus 2 | Cluster 1 | 7.0 | 7.0 | 6.9 | 8.7 | 9.1 | 9.5 | 4.4 | 7.94E-04 |
| **10502335** | *Bank1* | B-cell scaffold protein with ankyrin repeats 1 | Cluster 1 | 7.8 | 7.6 | 7.6 | 9.3 | 9.9 | 10.1 | 4.4 | 1.09E-03 |
| **10550509** | *Pglyrp1* | peptidoglycan recognition protein 1 | Cluster 1 | 8.6 | 8.5 | 8.6 | 10.3 | 10.7 | 11.1 | 4.4 | 7.94E-04 |
| **10545242** | *Igk-V19-20|Igk-V28* | immunoglobulin kappa chain variable 19 (V19)-20 | immunoglobulin kappa chain variable 28 (V28) | Cluster 1 | 6.3 | 6.3 | 6.3 | 7.7 | 7.7 | 9.9 | 4.3 | 4.37E-02 |
| **10392142** | *Cd79b* | CD79B antigen | Cluster 1 | 9.1 | 9.0 | 8.9 | 10.7 | 11.1 | 11.4 | 4.3 | 6.07E-04 |
| **10403046** | *AI324046* |  | Cluster 1 | 5.7 | 5.6 | 5.9 | 6.9 | 8.6 | 7.9 | 4.3 | 1.46E-02 |
| **10403009** | *Ighg|LOC100045391* | Immunoglobulin heavy chain (gamma polypeptide) | ig heavy chain V region M315-like | Cluster 1 | 6.5 | 6.9 | 6.4 | 8.1 | 8.6 | 9.3 | 4.2 | 5.55E-03 |
| **10593015** | *Cd3g* | CD3 antigen, gamma polypeptide | Cluster 1 | 8.1 | 8.2 | 8.0 | 9.8 | 10.2 | 10.5 | 4.1 | 6.07E-04 |
| **10563597** | *Saa3* | serum amyloid A 3 | Cluster 1 | 7.1 | 6.9 | 7.2 | 8.5 | 8.9 | 9.8 | 4.1 | 7.13E-03 |
| **10522788** | *Stap1* | signal transducing adaptor family member 1 | Cluster 1 | 7.4 | 7.1 | 7.3 | 8.8 | 9.4 | 9.7 | 4.1 | 1.88E-03 |
| **10567863** | *Cd19* | CD19 antigen | Cluster 1 | 7.4 | 7.3 | 7.2 | 8.9 | 9.4 | 9.7 | 4.0 | 1.07E-03 |
| **10402864** | *Ighg…* | Immunoglobulin heavy chain (gamma polypeptide)… | Cluster 1 | 7.7 | 7.6 | 7.5 | 9.3 | 9.4 | 10.0 | 3.9 | 9.61E-04 |
| **10547894** | *Cd4* | CD4 antigen | Cluster 1 | 7.8 | 7.9 | 7.8 | 9.5 | 9.6 | 10.2 | 3.8 | 9.43E-04 |
| **10545200** |  |  | Cluster 1 | 6.4 | 6.3 | 6.0 | 7.7 | 8.1 | 8.6 | 3.8 | 2.69E-03 |
| **10403057** |  |  | Cluster 1 | 6.5 | 6.0 | 6.1 | 7.6 | 8.1 | 8.5 | 3.7 | 3.47E-03 |
| **10406928** | *Cd180* | CD180 antigen | Cluster 1 | 8.5 | 8.2 | 8.4 | 9.9 | 10.2 | 10.5 | 3.6 | 7.04E-04 |
| **10512372** | *Ccl19|LOC100043921|Gm13309|Gm2442* | chemokine (C-C motif) ligand 19 | c-C motif chemokine 19-like | predicted gene 13309 | predicted gene 2442 | Cluster 1 | 8.3 | 8.2 | 8.2 | 9.6 | 9.9 | 10.7 | 3.6 | 5.14E-03 |
| **10466200** | *Ms4a7* | membrane-spanning 4-domains, subfamily A, member 7 | Cluster 1 | 6.7 | 6.7 | 6.7 | 7.9 | 8.8 | 8.9 | 3.5 | 4.49E-03 |
| **10512322** | *Ccl19|LOC100043921|Gm13309|Gm2442* | chemokine (C-C motif) ligand 19 | c-C motif chemokine 19-like | predicted gene 13309 | predicted gene 2442 | Cluster 1 | 8.4 | 8.3 | 8.3 | 9.7 | 9.9 | 10.7 | 3.5 | 4.54E-03 |
| **10504159** | *Ccl19|LOC100043921|Gm13309|Gm2442* | chemokine (C-C motif) ligand 19 | c-C motif chemokine 19-like | predicted gene 13309 | predicted gene 2442 | Cluster 1 | 8.4 | 8.3 | 8.3 | 9.7 | 9.9 | 10.7 | 3.5 | 4.54E-03 |
| **10585276** | *Pou2af1* | POU domain, class 2, associating factor 1 | Cluster 1 | 7.9 | 8.2 | 7.7 | 9.3 | 9.8 | 10.0 | 3.5 | 2.24E-03 |
| **10504188** | *Ccl19|LOC100043921|Gm13309|Gm2442* | chemokine (C-C motif) ligand 19 | c-C motif chemokine 19-like | predicted gene 13309 | predicted gene 2442 | Cluster 1 | 8.3 | 8.3 | 8.3 | 9.6 | 9.9 | 10.6 | 3.4 | 4.26E-03 |
| **10403821** | *Tcrg-V3|Tcrg-V2* | T-cell receptor gamma, variable 3 | T-cell receptor gamma, variable 2 | Cluster 1 | 8.0 | 7.6 | 7.8 | 8.9 | 10.0 | 9.7 | 3.3 | 7.59E-03 |
| **10486061** | *Rasgrp1* | RAS guanyl releasing protein 1 | Cluster 1 | 8.2 | 8.2 | 8.1 | 9.6 | 9.9 | 10.1 | 3.3 | 3.37E-04 |
| **10404606** | *Ly86* | lymphocyte antigen 86 | Cluster 1 | 9.5 | 9.4 | 9.5 | 10.9 | 11.3 | 11.4 | 3.3 | 3.77E-04 |
| **10601416** | *P2ry10* | purinergic receptor P2Y, G-protein coupled 10 | Cluster 1 | 6.9 | 6.6 | 6.6 | 8.0 | 8.5 | 8.7 | 3.2 | 1.81E-03 |
| **10597996** | *Xcr1* | chemokine (C motif) receptor 1 | Cluster 1 | 7.9 | 8.0 | 7.9 | 9.3 | 9.6 | 10.0 | 3.2 | 1.16E-03 |
| **10351691** | *Slamf6* | SLAM family member 6 | Cluster 1 | 7.7 | 7.6 | 7.6 | 8.9 | 9.4 | 9.6 | 3.1 | 1.38E-03 |
| **10545245** |  |  | Cluster 1 | 6.0 | 5.9 | 5.8 | 7.6 | 6.5 | 8.5 | 3.1 | 4.83E-02 |
| **10403023** | *Gm7016* | predicted gene 7016 | Cluster 1 | 5.4 | 5.5 | 5.6 | 6.8 | 6.8 | 7.7 | 3.1 | 6.35E-03 |
| **10504132** | *Ccl19|LOC100043921|Gm13309|Gm2442* | chemokine (C-C motif) ligand 19 | c-C motif chemokine 19-like | predicted gene 13309 | predicted gene 2442 | Cluster 1 | 8.4 | 8.3 | 8.4 | 9.6 | 9.8 | 10.6 | 3.1 | 6.03E-03 |
| **10517513** | *C1qc* | complement component 1, q subcomponent, C chain | Cluster 1 | 8.7 | 8.8 | 8.6 | 10.1 | 10.2 | 10.7 | 3.1 | 1.10E-03 |
| **10563178** | *Cd37* | CD37 antigen | Cluster 1 | 8.8 | 8.7 | 8.7 | 10.1 | 10.2 | 10.7 | 3.0 | 1.06E-03 |
| **10429560** | *Ly6i* | lymphocyte antigen 6 complex, locus I | Cluster 1 | 7.4 | 7.4 | 7.4 | 8.5 | 9.0 | 9.4 | 3.0 | 3.84E-03 |
| **10346799** | *Icos* | inducible T-cell co-stimulator | Cluster 1 | 7.5 | 7.7 | 7.4 | 8.6 | 9.1 | 9.6 | 3.0 | 6.56E-03 |
| **10590623** | *Cxcr6* | chemokine (C-X-C motif) receptor 6 | Cluster 1 | 7.1 | 7.0 | 7.2 | 8.1 | 8.8 | 9.1 | 3.0 | 6.56E-03 |
| **10492971** | *Fcrl1* | Fc receptor-like 1 | Cluster 1 | 6.9 | 6.9 | 6.9 | 8.0 | 8.6 | 8.8 | 2.9 | 2.86E-03 |
| **10461594** | *Ms4a4c* | membrane-spanning 4-domains, subfamily A, member 4C | Cluster 1 | 7.5 | 7.4 | 7.5 | 8.8 | 8.8 | 9.4 | 2.9 | 1.64E-03 |
| **10390640** | *Ikzf3* | IKAROS family zinc finger 3 | Cluster 1 | 7.6 | 7.6 | 7.7 | 8.7 | 9.2 | 9.5 | 2.9 | 3.13E-03 |
| **10403825** | *Tcrg-C* | T-cell receptor gamma, constant region | Cluster 1 | 6.7 | 6.7 | 6.5 | 7.8 | 8.3 | 8.4 | 2.9 | 1.48E-03 |
| **10557177** | *Prkcb* | protein kinase C, beta | Cluster 1 | 8.3 | 8.4 | 8.2 | 9.4 | 9.9 | 10.0 | 2.8 | 1.65E-03 |
| **10351873** | *Pyhin1* | pyrin and HIN domain family, member 1 | Cluster 1 | 6.9 | 6.7 | 6.6 | 7.9 | 8.3 | 8.5 | 2.8 | 1.60E-03 |
| **10430818** | *Tnfrsf13c* | tumor necrosis factor receptor superfamily, member 13c | Cluster 1 | 8.9 | 9.0 | 8.7 | 10.0 | 10.0 | 10.9 | 2.8 | 1.02E-02 |
| **10466210** | *Ms4a6d* | membrane-spanning 4-domains, subfamily A, member 6D | Cluster 1 | 9.0 | 8.7 | 8.9 | 10.0 | 10.5 | 10.5 | 2.8 | 1.47E-03 |
| **10444236** | *H2-DMb2|H2-DMb1* | histocompatibility 2, class II, locus Mb2 | histocompatibility 2, class II, locus Mb1 | Cluster 1 | 10.0 | 9.8 | 9.9 | 11.0 | 11.4 | 11.6 | 2.8 | 1.51E-03 |
| **10347888** | *Ccl20* | chemokine (C-C motif) ligand 20 | Cluster 1 | 7.5 | 7.8 | 7.6 | 8.2 | 9.4 | 9.7 | 2.8 | 3.48E-02 |
| **10467508** | *Blnk* | B-cell linker | Cluster 1 | 7.9 | 7.7 | 7.8 | 9.0 | 9.2 | 9.6 | 2.8 | 1.39E-03 |
| **10444284** | *H2-Ob* | histocompatibility 2, O region beta locus | Cluster 1 | 8.2 | 8.1 | 8.2 | 9.2 | 9.7 | 10.0 | 2.8 | 3.40E-03 |
| **10517508** | *C1qb* | complement component 1, q subcomponent, beta polypeptide | Cluster 1 | 9.9 | 9.9 | 9.8 | 11.1 | 11.3 | 11.6 | 2.8 | 5.99E-04 |
| **10450325** | *Cfb* | complement factor B | Cluster 1 | 8.8 | 8.6 | 8.7 | 10.0 | 9.9 | 10.5 | 2.7 | 1.80E-03 |
| **10403038** | *LOC382693|Gm16710* | similar to immunoglobulin heavy chain | predicted gene, 16710 | Cluster 1 | 6.1 | 6.2 | 6.1 | 7.0 | 7.4 | 8.2 | 2.7 | 1.68E-02 |
| **10547657** | *C3ar1* | complement component 3a receptor 1 | Cluster 1 | 8.2 | 8.3 | 8.2 | 9.4 | 9.8 | 9.8 | 2.7 | 4.78E-04 |
| **10530145** | *Tlr1* | toll-like receptor 1 | Cluster 1 | 6.8 | 6.9 | 7.0 | 8.0 | 8.4 | 8.6 | 2.7 | 1.51E-03 |
| **10349648** | *Ctse* | cathepsin E | Cluster 1 | 8.9 | 8.8 | 8.8 | 10.1 | 10.2 | 10.6 | 2.7 | 7.19E-04 |
| **10601424** | *Gpr174* | G protein-coupled receptor 174 | Cluster 1 | 6.0 | 6.1 | 6.0 | 7.1 | 7.7 | 7.6 | 2.7 | 1.61E-03 |
| **10517517** | *C1qa* | complement component 1, q subcomponent, alpha polypeptide | Cluster 1 | 8.6 | 8.5 | 8.3 | 9.5 | 9.8 | 10.3 | 2.7 | 4.95E-03 |
| **10389214** | *Ccl9* | chemokine (C-C motif) ligand 9 | Cluster 1 | 8.7 | 8.9 | 8.9 | 9.9 | 10.4 | 10.5 | 2.7 | 1.90E-03 |
| **10591739** | *Acp5* | acid phosphatase 5, tartrate resistant | Cluster 1 | 8.6 | 8.7 | 8.7 | 9.9 | 10.1 | 10.3 | 2.7 | 2.83E-04 |
| **10525365** | *Hvcn1|Tctn1* | hydrogen voltage-gated channel 1 | tectonic family member 1 | Cluster 1 | 8.9 | 8.7 | 8.7 | 9.9 | 10.1 | 10.5 | 2.7 | 1.76E-03 |
| **10455970** | *BC023105* |  | Cluster 1 | 7.0 | 6.7 | 6.3 | 7.9 | 7.8 | 8.5 | 2.6 | 9.34E-03 |
| **10471912** | *Kynu* | kynureninase (L-kynurenine hydrolase) | Cluster 1 | 7.3 | 7.2 | 7.3 | 8.3 | 8.7 | 8.9 | 2.6 | 1.60E-03 |
| **10408557** | *Serpinb1a* | serine (or cysteine) peptidase inhibitor, clade B, member 1a | Cluster 1 | 7.9 | 7.8 | 7.9 | 8.6 | 9.5 | 9.6 | 2.6 | 1.29E-02 |
| **10531737** | *Hpse* | heparanase | Cluster 1 | 8.3 | 8.4 | 8.1 | 9.3 | 9.6 | 10.1 | 2.6 | 4.95E-03 |
| **10432675** | *I730030J21Rik* | RIKEN cDNA I730030J21 gene | Cluster 1 | 6.7 | 6.7 | 6.8 | 7.6 | 8.1 | 8.6 | 2.6 | 9.29E-03 |
| **10407940** | *Tcrg-V2|Tcrg-V1* | T-cell receptor gamma, variable 2 | T-cell receptor gamma, variable 1 | Cluster 1 | 7.7 | 7.5 | 7.6 | 8.2 | 9.5 | 9.2 | 2.6 | 2.63E-02 |
| **10592888** | *Cxcr5* | chemochine (C-X-C motif) receptor 5 | Cluster 1 | 6.0 | 5.8 | 5.9 | 6.8 | 7.2 | 7.8 | 2.6 | 9.93E-03 |
| **10590631** | *Ccr2* | chemokine (C-C motif) receptor 2 | Cluster 1 | 7.5 | 7.6 | 7.5 | 8.5 | 9.1 | 9.0 | 2.6 | 2.11E-03 |
| **10407982** | *A530099J19Rik* | RIKEN cDNA A530099J19 gene | Cluster 1 | 8.2 | 8.0 | 8.0 | 9.1 | 9.6 | 9.6 | 2.5 | 1.60E-03 |
| **10414811** | *Gm13969* | predicted gene 13969 | Cluster 1 | 5.9 | 6.0 | 5.9 | 6.9 | 7.2 | 7.7 | 2.5 | 4.81E-03 |
| **10455961** | *Iigp1* | interferon inducible GTPase 1 | Cluster 1 | 8.4 | 8.4 | 8.1 | 9.4 | 9.6 | 10.0 | 2.5 | 2.52E-03 |
| **10414937** | *Gm13969* | predicted gene 13969 | Cluster 1 | 5.9 | 6.0 | 5.9 | 6.9 | 7.2 | 7.7 | 2.5 | 4.81E-03 |
| **10593024** | *Cd3e* | CD3 antigen, epsilon polypeptide | Cluster 1 | 9.1 | 9.0 | 8.8 | 10.0 | 10.0 | 10.9 | 2.5 | 1.30E-02 |
| **10399854** | *Slc26a4* | solute carrier family 26, member 4 | Cluster 1 | 6.8 | 7.1 | 6.7 | 8.0 | 8.0 | 8.5 | 2.5 | 3.19E-03 |
| **10430372** | *Rac2* | RAS-related C3 botulinum substrate 2 | Cluster 1 | 9.5 | 9.5 | 9.5 | 10.6 | 10.6 | 11.3 | 2.5 | 4.64E-03 |
| **10603860** | *Cfp* | complement factor properdin | Cluster 1 | 9.0 | 9.0 | 9.0 | 10.1 | 10.2 | 10.6 | 2.5 | 1.05E-03 |
| **10385533** | *Tgtp1|Tgtp2|Gm12185* | T-cell specific GTPase 1 | T-cell specific GTPase 2 | predicted gene 12185 | Cluster 1 | 9.7 | 9.7 | 9.4 | 10.7 | 10.8 | 11.2 | 2.5 | 2.06E-03 |
| **10567825** | *Lat* | linker for activation of T cells | Cluster 1 | 7.7 | 7.8 | 7.7 | 8.7 | 9.1 | 9.4 | 2.5 | 2.91E-03 |
| **10444752** | *Ltb* | lymphotoxin B | Cluster 1 | 7.7 | 7.9 | 8.0 | 8.8 | 9.2 | 9.6 | 2.5 | 5.72E-03 |
| **10439312** | *Cd86* | CD86 antigen | Cluster 1 | 7.1 | 7.2 | 7.0 | 8.0 | 8.5 | 8.7 | 2.4 | 3.84E-03 |
| **10516620** | *Lck* | lymphocyte protein tyrosine kinase | Cluster 1 | 8.1 | 8.3 | 8.1 | 9.1 | 9.4 | 9.8 | 2.4 | 4.04E-03 |
| **10519497** | *Steap4* | STEAP family member 4 | Cluster 1 | 8.7 | 8.8 | 8.7 | 9.7 | 9.9 | 10.4 | 2.4 | 3.86E-03 |
| **10351658** | *Cd48* | CD48 antigen | Cluster 1 | 7.5 | 7.5 | 7.4 | 8.4 | 8.9 | 8.8 | 2.4 | 1.40E-03 |
| **10444306** | *H2-Eb2* | histocompatibility 2, class II antigen E beta2 | Cluster 1 | 6.7 | 6.8 | 6.6 | 7.7 | 8.0 | 8.1 | 2.4 | 7.59E-04 |
| **10461614** | *Ms4a6c* | membrane-spanning 4-domains, subfamily A, member 6C | Cluster 1 | 9.0 | 9.1 | 9.0 | 10.0 | 10.5 | 10.4 | 2.4 | 1.26E-03 |
| **10385518** | *Tgtp1|Tgtp2|Gm12185* | T-cell specific GTPase 1 | T-cell specific GTPase 2 | predicted gene 12185 | Cluster 1 | 9.7 | 9.6 | 9.5 | 10.7 | 10.8 | 11.2 | 2.4 | 1.35E-03 |
| **10547621** | *Apobec1* | apolipoprotein B mRNA editing enzyme, catalytic polypeptide 1 | Cluster 1 | 8.6 | 8.6 | 8.5 | 9.4 | 10.0 | 10.1 | 2.4 | 4.60E-03 |
| **10494271** | *Ctss* | cathepsin S | Cluster 1 | 10.0 | 9.9 | 9.9 | 10.8 | 11.2 | 11.4 | 2.4 | 2.60E-03 |
| **10531415** | *Cxcl10* | chemokine (C-X-C motif) ligand 10 | Cluster 1 | 7.3 | 7.2 | 7.3 | 8.1 | 8.2 | 9.2 | 2.4 | 2.50E-02 |
| **10404389** | *Irf4* | interferon regulatory factor 4 | Cluster 1 | 6.9 | 7.0 | 7.0 | 7.9 | 8.2 | 8.5 | 2.4 | 2.20E-03 |
| **10590635** | *Ccr5|Ccr2* | chemokine (C-C motif) receptor 5 | chemokine (C-C motif) receptor 2 | Cluster 1 | 6.6 | 6.7 | 6.8 | 7.6 | 7.9 | 8.2 | 2.4 | 2.77E-03 |
| **10404840** | *Cd83* | CD83 antigen | Cluster 1 | 8.2 | 8.0 | 7.9 | 9.0 | 9.3 | 9.5 | 2.4 | 1.92E-03 |
| **10597960** | *Slc6a20a* | solute carrier family 6 (neurotransmitter transporter), member 20A | Cluster 1 | 8.3 | 8.1 | 8.3 | 9.2 | 9.5 | 9.8 | 2.4 | 2.41E-03 |
| **10598013** | *Ccr5|Ccr2* | chemokine (C-C motif) receptor 5 | chemokine (C-C motif) receptor 2 | Cluster 1 | 6.6 | 6.7 | 6.8 | 7.6 | 7.9 | 8.2 | 2.3 | 2.77E-03 |
| **10461622** | *Ms4a6b* | membrane-spanning 4-domains, subfamily A, member 6B | Cluster 1 | 8.2 | 8.4 | 8.1 | 9.2 | 9.6 | 9.7 | 2.3 | 1.99E-03 |
| **10439744** | *Cd96* | CD96 antigen | Cluster 1 | 6.9 | 7.0 | 6.8 | 7.8 | 8.1 | 8.5 | 2.3 | 4.26E-03 |
| **10387699** | *Acap1* | ArfGAP with coiled-coil, ankyrin repeat and PH domains 1 | Cluster 1 | 7.8 | 8.0 | 7.8 | 8.8 | 9.1 | 9.4 | 2.3 | 2.66E-03 |
| **10557342** | *Il21r* | interleukin 21 receptor | Cluster 1 | 7.4 | 7.4 | 7.3 | 8.3 | 8.4 | 9.0 | 2.3 | 5.59E-03 |
| **10599487** | *Sash3* | SAM and SH3 domain containing 3 | Cluster 1 | 8.7 | 8.7 | 8.6 | 9.7 | 9.8 | 10.1 | 2.3 | 6.52E-04 |
| **10562132** | *Cd22* | CD22 antigen | Cluster 1 | 8.1 | 8.0 | 8.0 | 9.0 | 9.2 | 9.5 | 2.3 | 1.29E-03 |
| **10501063** | *Cd53* | CD53 antigen | Cluster 1 | 10.4 | 10.5 | 10.4 | 11.4 | 11.8 | 11.8 | 2.3 | 8.53E-04 |
| **10430344** | *Il2rb* | interleukin 2 receptor, beta chain | Cluster 1 | 8.1 | 8.0 | 8.1 | 9.0 | 9.1 | 9.7 | 2.3 | 5.59E-03 |
| **10548817** | *Plbd1* | phospholipase B domain containing 1 | Cluster 1 | 9.9 | 9.8 | 9.8 | 10.8 | 11.0 | 11.2 | 2.3 | 6.30E-04 |
| **10568024** | *Coro1a* | coronin, actin binding protein 1A | Cluster 1 | 8.6 | 8.6 | 8.6 | 9.6 | 9.5 | 10.4 | 2.3 | 1.23E-02 |
| **10435982** | *Btla* | B and T lymphocyte associated | Cluster 1 | 7.9 | 7.6 | 7.5 | 8.4 | 9.0 | 9.1 | 2.3 | 9.47E-03 |
| **10360158** | *Ly9* | lymphocyte antigen 9 | Cluster 1 | 7.9 | 8.0 | 7.9 | 8.7 | 9.2 | 9.4 | 2.3 | 5.21E-03 |
| **10389143** | *Slfn8* | schlafen 8 | Cluster 1 | 7.0 | 6.8 | 6.8 | 7.9 | 7.9 | 8.3 | 2.3 | 1.44E-03 |
| **10444298** | *H2-Eb1* | histocompatibility 2, class II antigen E beta | Cluster 1 | 10.6 | 10.5 | 10.5 | 11.5 | 11.6 | 12.0 | 2.3 | 1.72E-03 |
| **10548333** | *Cd69* | CD69 antigen | Cluster 1 | 7.0 | 7.0 | 6.7 | 7.6 | 8.3 | 8.3 | 2.3 | 1.01E-02 |
| **10433507** | *Ciita* | class II transactivator | Cluster 1 | 8.1 | 8.1 | 8.1 | 9.0 | 9.3 | 9.5 | 2.3 | 1.31E-03 |
| **10398907** | *Pld4* | phospholipase D family, member 4 | Cluster 1 | 9.3 | 9.2 | 9.2 | 10.1 | 10.3 | 10.7 | 2.2 | 3.22E-03 |
| **10450374** | *D17H6S56E-5* | DNA segment, Chr 17, human D6S56E 5 | Cluster 1 | 9.4 | 9.6 | 9.4 | 10.3 | 10.8 | 10.8 | 2.2 | 2.89E-03 |
| **10378286** | *Itgae* | integrin alpha E, epithelial-associated | Cluster 1 | 7.8 | 7.8 | 7.8 | 8.6 | 9.0 | 9.3 | 2.2 | 4.52E-03 |
| **10379524** | *Ccl11* | chemokine (C-C motif) ligand 11 | Cluster 1 | 8.2 | 8.3 | 8.2 | 9.2 | 9.4 | 9.6 | 2.2 | 6.30E-04 |
| **10496539** | *Gbp5* | guanylate binding protein 5 | Cluster 1 | 7.1 | 7.1 | 7.1 | 7.8 | 8.2 | 8.8 | 2.2 | 1.59E-02 |
| **10360018** | *Fcrla* | Fc receptor-like A | Cluster 1 | 7.9 | 7.7 | 7.8 | 8.7 | 9.0 | 9.2 | 2.2 | 1.72E-03 |
| **10374333** | *Ikzf1* | IKAROS family zinc finger 1 | Cluster 1 | 8.5 | 8.4 | 8.3 | 9.3 | 9.5 | 9.8 | 2.2 | 1.92E-03 |
| **10439527** | *Tigit* | T cell immunoreceptor with Ig and ITIM domains | Cluster 1 | 8.3 | 8.4 | 8.4 | 9.1 | 9.5 | 9.9 | 2.2 | 8.30E-03 |
| **10542164** | *Clec12a* | C-type lectin domain family 12, member a | Cluster 1 | 7.9 | 8.0 | 8.1 | 8.7 | 9.3 | 9.3 | 2.2 | 6.15E-03 |
| **10360173** | *Slamf7* | SLAM family member 7 | Cluster 1 | 7.8 | 7.7 | 7.7 | 8.4 | 8.9 | 9.2 | 2.2 | 9.54E-03 |
| **10605034** | *Xlr4c|Xlr4b|Xlr4a* | X-linked lymphocyte-regulated 4C | X-linked lymphocyte-regulated 4B | X-linked lymphocyte-regulated 4A | Cluster 1 | 6.3 | 6.4 | 6.2 | 7.0 | 7.6 | 7.6 | 2.2 | 6.15E-03 |
| **10422760** | *Fyb* | FYN binding protein | Cluster 1 | 7.9 | 7.7 | 7.8 | 8.6 | 9.2 | 9.0 | 2.2 | 3.64E-03 |
| **10597420** | *Ccr4* | chemokine (C-C motif) receptor 4 | Cluster 1 | 6.9 | 7.2 | 7.0 | 7.7 | 8.2 | 8.6 | 2.2 | 1.46E-02 |
| **10445412** | *Nfkbie* | nuclear factor of kappa light polypeptide gene enhancer in B-cells inhibitor, epsilon | Cluster 1 | 7.7 | 7.8 | 8.0 | 8.8 | 8.8 | 9.4 | 2.2 | 5.94E-03 |
| **10446253** | *Vav1* | vav 1 oncogene | Cluster 1 | 7.8 | 8.0 | 7.9 | 8.7 | 9.2 | 9.3 | 2.2 | 3.88E-03 |
| **10409240** | *Sema4d* | sema domain, immunoglobulin domain (Ig), transmembrane domain (TM) and short cytoplasmic domain, (semaphorin) 4D | Cluster 1 | 8.5 | 8.4 | 8.4 | 9.3 | 9.6 | 9.7 | 2.2 | 9.12E-04 |
| **10552743** | *Il4i1|Nup62-il4i1* | interleukin 4 induced 1 | Nup62-Il4i1 protein | Cluster 1 | 7.0 | 6.9 | 7.3 | 7.8 | 8.2 | 8.5 | 2.2 | 9.54E-03 |
| **10551666** | *Map4k1* | mitogen-activated protein kinase kinase kinase kinase 1 | Cluster 1 | 8.4 | 8.3 | 8.4 | 9.2 | 9.4 | 9.8 | 2.2 | 3.59E-03 |
| **10358224** | *Ptprc* | protein tyrosine phosphatase, receptor type, C | Cluster 1 | 9.9 | 10.0 | 9.9 | 10.8 | 11.1 | 11.3 | 2.2 | 1.61E-03 |
| **10360382** | *Ifi204|Mnda* | interferon activated gene 204 | myeloid cell nuclear differentiation antigen | Cluster 1 | 8.7 | 8.3 | 8.3 | 9.3 | 9.5 | 9.8 | 2.2 | 5.06E-03 |
| **10494978** | *Ptpn22* | protein tyrosine phosphatase, non-receptor type 22 (lymphoid) | Cluster 1 | 8.0 | 8.0 | 7.8 | 8.8 | 9.1 | 9.2 | 2.1 | 1.32E-03 |
| **10358408** | *Rgs1* | regulator of G-protein signaling 1 | Cluster 1 | 6.5 | 6.5 | 6.4 | 7.2 | 7.9 | 7.7 | 2.1 | 5.78E-03 |
| **10606694** | *Btk* | Bruton agammaglobulinemia tyrosine kinase | Cluster 1 | 7.3 | 7.2 | 7.2 | 8.0 | 8.5 | 8.5 | 2.1 | 2.94E-03 |
| **10512669** | *Pax5* | paired box gene 5 | Cluster 1 | 7.9 | 7.8 | 7.6 | 8.6 | 8.9 | 9.1 | 2.1 | 2.94E-03 |
| **10582985** | *Casp1* | caspase 1 | Cluster 1 | 7.5 | 7.2 | 7.1 | 8.1 | 8.4 | 8.6 | 2.1 | 4.30E-03 |
| **10349571** | *Fcamr* | Fc receptor, IgA, IgM, high affinity | Cluster 1 | 7.3 | 7.6 | 7.5 | 8.1 | 8.6 | 8.9 | 2.1 | 1.29E-02 |
| **10360370** | *BC094916* |  | Cluster 1 | 6.3 | 6.4 | 6.5 | 7.3 | 7.0 | 8.1 | 2.1 | 3.29E-02 |
| **10461765** | *Lpxn* | leupaxin | Cluster 1 | 7.7 | 7.7 | 7.6 | 8.4 | 8.9 | 8.9 | 2.1 | 3.29E-03 |
| **10593050** | *Il10ra* | interleukin 10 receptor, alpha | Cluster 1 | 8.1 | 8.1 | 8.0 | 8.8 | 9.1 | 9.5 | 2.1 | 6.56E-03 |
| **10427628** | *Il7r* | interleukin 7 receptor | Cluster 1 | 9.4 | 9.5 | 9.3 | 10.2 | 10.5 | 10.7 | 2.1 | 2.41E-03 |
| **10354374** | *Slc40a1* | solute carrier family 40 (iron-regulated transporter), member 1 | Cluster 1 | 7.7 | 7.5 | 7.5 | 8.2 | 8.7 | 8.9 | 2.1 | 9.12E-03 |
| **10375145** | *Lcp2* | lymphocyte cytosolic protein 2 | Cluster 1 | 9.2 | 9.0 | 9.1 | 9.9 | 10.1 | 10.4 | 2.1 | 2.72E-03 |
| **10444291** | *H2-Ab1* | histocompatibility 2, class II antigen A, beta 1 | Cluster 1 | 10.4 | 10.3 | 10.4 | 11.3 | 11.4 | 11.7 | 2.1 | 9.12E-04 |
| **10427336** | *Nckap1l* | NCK associated protein 1 like | Cluster 1 | 9.4 | 9.5 | 9.5 | 10.3 | 10.6 | 10.7 | 2.1 | 1.03E-03 |
| **10381708** | *Fmnl1* | formin-like 1 | Cluster 1 | 8.6 | 8.6 | 8.5 | 9.4 | 9.6 | 9.8 | 2.1 | 1.01E-03 |
| **10468898** | *Lax1* | lymphocyte transmembrane adaptor 1 | Cluster 1 | 7.4 | 7.6 | 7.3 | 8.2 | 8.4 | 8.7 | 2.1 | 4.17E-03 |
| **10449893** | *Rasal3|A530088E08Rik* | RAS protein activator like 3 | RIKEN cDNA A530088E08 gene | Cluster 1 | 7.9 | 7.9 | 8.0 | 8.8 | 8.8 | 9.3 | 2.1 | 3.70E-03 |
| **10390763** | *Ccr7* | chemokine (C-C motif) receptor 7 | Cluster 1 | 8.3 | 8.6 | 8.4 | 9.2 | 9.3 | 9.9 | 2.0 | 1.18E-02 |
| **10346783** | *Cd28* | CD28 antigen | Cluster 1 | 7.3 | 7.3 | 7.1 | 8.0 | 8.2 | 8.7 | 2.0 | 8.16E-03 |
| **10425053** | *Ncf4* | neutrophil cytosolic factor 4 | Cluster 1 | 7.7 | 8.0 | 7.9 | 8.6 | 8.7 | 9.3 | 2.0 | 1.32E-02 |
| **10413615** | *Itih4* | inter alpha-trypsin inhibitor, heavy chain 4 | Cluster 1 | 10.0 | 9.8 | 9.9 | 10.8 | 10.8 | 11.2 | 2.0 | 2.07E-03 |
| **10397645** | *Gpr65* | G-protein coupled receptor 65 | Cluster 1 | 7.2 | 7.4 | 7.1 | 7.9 | 8.5 | 8.4 | 2.0 | 7.34E-03 |
| **10574098** | *Nlrc5* | NLR family, CARD domain containing 5 | Cluster 1 | 8.6 | 8.7 | 8.8 | 9.5 | 9.7 | 10.0 | 2.0 | 2.72E-03 |
| **10575799** | *Plcg2* | phospholipase C, gamma 2 | Cluster 1 | 8.5 | 8.2 | 8.4 | 9.1 | 9.4 | 9.6 | 2.0 | 4.17E-03 |
| **10605143** | *Arhgap4* | Rho GTPase activating protein 4 | Cluster 1 | 8.1 | 8.1 | 7.9 | 8.9 | 9.1 | 9.3 | 2.0 | 1.32E-03 |
| **10522182** | *Rhoh* | ras homolog gene family, member H | Cluster 1 | 7.7 | 7.8 | 7.8 | 8.5 | 8.8 | 9.1 | 2.0 | 4.24E-03 |
| **10425410** | *Grap2* | GRB2-related adaptor protein 2 | Cluster 1 | 8.5 | 8.5 | 8.5 | 9.2 | 9.5 | 9.7 | 2.0 | 2.65E-03 |
| **10376324** | *Gm12250* | predicted gene 12250 | Cluster 1 | 8.6 | 8.6 | 8.7 | 9.5 | 9.5 | 9.9 | 2.0 | 1.90E-03 |
| **10557895** | *Itgax* | integrin alpha X | Cluster 1 | 9.2 | 9.0 | 9.1 | 9.8 | 10.2 | 10.4 | 2.0 | 5.10E-03 |
| **10487011** | *Gatm* | glycine amidinotransferase (L-arginine:glycine amidinotransferase) | Cluster 1 | 10.0 | 9.9 | 9.9 | 10.7 | 11.0 | 11.1 | 2.0 | 1.31E-03 |
| **10481627** | *Lcn2* | lipocalin 2 | Cluster 1 | 12.0 | 11.9 | 11.9 | 12.8 | 12.8 | 13.2 | 2.0 | 1.90E-03 |
| **10435565** | *Hcls1* | hematopoietic cell specific Lyn substrate 1 | Cluster 1 | 9.1 | 9.1 | 9.1 | 9.9 | 10.0 | 10.4 | 2.0 | 2.81E-03 |
| **10438098** | *Sdf2l1* | stromal cell-derived factor 2-like 1 | Cluster 1 | 9.1 | 9.3 | 9.1 | 10.0 | 10.0 | 10.4 | 2.0 | 2.92E-03 |
| **10361215** | *Traf3ip3* | TRAF3 interacting protein 3 | Cluster 1 | 6.9 | 6.9 | 6.9 | 7.6 | 7.9 | 8.2 | 2.0 | 4.47E-03 |
| **10460371** | *Ptprcap|Rps6kb2* | protein tyrosine phosphatase, receptor type, C polypeptide-associated protein | ribosomal protein S6 kinase, polypeptide 2 | Cluster 1 | 8.1 | 8.4 | 8.4 | 9.0 | 9.2 | 9.6 | 2.0 | 8.86E-03 |
| **10578264** | *Msr1* | macrophage scavenger receptor 1 | Cluster 1 | 7.2 | 7.2 | 7.2 | 7.8 | 8.4 | 8.4 | 2.0 | 7.49E-03 |
| **10566583** | *Gm8995* | predicted gene 8995 | Cluster 1 | 9.1 | 9.2 | 9.0 | 10.0 | 10.1 | 10.3 | 2.0 | 6.07E-04 |
| **10502622** | *Clca3* | chloride channel calcium activated 3 | Cluster 2 | 7.0 | 6.6 | 6.8 | 11.8 | 11.5 | 11.7 | 29.4 | 4.74E-06 |
| **10433172** | *Glycam1* | glycosylation dependent cell adhesion molecule 1 | Cluster 2 | 6.5 | 6.9 | 6.4 | 11.2 | 10.8 | 11.4 | 23.1 | 4.14E-05 |
| **10538882** | *Gm5571* | predicted gene 5571 | Cluster 2 | 8.2 | 7.3 | 7.3 | 11.9 | 11.9 | 12.0 | 19.3 | 1.37E-04 |
| **10538880** | *Igk-V1* | immunoglobulin kappa chain variable 1 (V1) | Cluster 2 | 6.8 | 5.9 | 5.5 | 10.1 | 9.3 | 10.4 | 14.2 | 1.57E-03 |
| **10545173** | *LOC672291* | similar to Ig kappa chain V-V region MOPC 173 | Cluster 2 | 7.7 | 6.8 | 7.4 | 11.2 | 11.7 | 10.6 | 14.2 | 7.29E-04 |
| **10531126** | *Igj* | immunoglobulin joining chain | Cluster 2 | 7.7 | 7.4 | 7.3 | 11.0 | 11.4 | 11.4 | 13.6 | 2.94E-05 |
| **10531407** | *Cxcl9* | chemokine (C-X-C motif) ligand 9 | Cluster 2 | 7.3 | 7.2 | 7.4 | 10.5 | 11.1 | 11.1 | 12.0 | 6.56E-05 |
| **10545175** | *LOC672291|Igk-J1* | similar to Ig kappa chain V-V region MOPC 173 | immunoglobulin kappa chain, joining region, 1 | Cluster 2 | 8.0 | 7.0 | 7.6 | 11.2 | 11.3 | 10.7 | 11.8 | 5.11E-04 |
| **10538921** |  |  | Cluster 2 | 8.4 | 7.9 | 7.8 | 11.5 | 11.1 | 11.6 | 10.3 | 1.51E-04 |
| **10538868** |  |  | Cluster 2 | 8.8 | 8.9 | 8.8 | 12.1 | 12.0 | 12.4 | 10.1 | 1.17E-05 |
| **10538871** | *Gm4964* | predicted gene 4964 | Cluster 2 | 7.5 | 7.3 | 7.1 | 10.5 | 10.6 | 10.7 | 9.6 | 1.39E-05 |
| **10545187** | *Gm1502|Gm8760|Igkv4-71* | predicted gene 1502 | predicted gene 8760 | immunoglobulin kappa chain variable 4-71 | Cluster 2 | 7.8 | 7.0 | 7.4 | 10.3 | 10.8 | 10.9 | 9.4 | 3.85E-04 |
| **10438405** | *Igl-V1|Igl-V2|LOC433053* | immunoglobulin lambda chain, variable 1 | immunoglobulin lambda chain, variable 2 | similar to Ig lambda-1 chain C region | Cluster 2 | 9.1 | 8.3 | 8.2 | 11.3 | 11.8 | 11.9 | 8.7 | 7.70E-04 |
| **10419568** | *Ear11* | eosinophil-associated, ribonuclease A family, member 11 | Cluster 2 | 6.1 | 5.9 | 6.1 | 9.8 | 8.3 | 9.3 | 8.6 | 2.25E-03 |
| **10545198** | *Igkv4-71|Gm8760|Gm1499* | immunoglobulin kappa chain variable 4-71 | predicted gene 8760 | predicted gene 1499 | Cluster 2 | 8.7 | 8.3 | 8.4 | 11.4 | 11.7 | 11.6 | 8.6 | 3.16E-05 |
| **10545190** |  |  | Cluster 2 | 5.6 | 5.2 | 5.4 | 8.2 | 8.6 | 8.6 | 8.5 | 6.42E-05 |
| **10438415** | *Igl-V2|Igl-C2* | immunoglobulin lambda chain, variable 2 | immunoglobulin lambda chain, constant region 2 | Cluster 2 | 7.6 | 7.3 | 7.3 | 10.1 | 10.6 | 10.5 | 8.0 | 8.03E-05 |
| **10545177** |  |  | Cluster 2 | 8.1 | 8.1 | 7.6 | 11.2 | 10.5 | 11.1 | 7.9 | 4.00E-04 |
| **10545184** | *Gm10880* | predicted gene 10880 | Cluster 2 | 8.3 | 7.8 | 7.9 | 10.8 | 11.0 | 11.0 | 7.8 | 6.12E-05 |
| **10402991** | *Gm16970|Igh-VX24* | predicted gene, 16970 | immunoglobulin heavy chain (X24 family) | Cluster 2 | 8.2 | 8.2 | 7.9 | 11.0 | 11.1 | 11.2 | 7.7 | 1.30E-05 |
| **10466172** | *Ms4a1* | membrane-spanning 4-domains, subfamily A, member 1 | Cluster 2 | 7.8 | 7.6 | 7.5 | 10.1 | 10.7 | 10.9 | 7.7 | 3.31E-04 |
| **10545205** | *Gm1418* | predicted gene 1418 | Cluster 2 | 7.5 | 6.6 | 6.9 | 9.7 | 10.0 | 9.8 | 7.1 | 5.29E-04 |
| **10403034** | *LOC100046275* | ig heavy chain V-II region SESS-like | Cluster 2 | 7.2 | 7.0 | 7.4 | 9.7 | 9.9 | 10.3 | 6.8 | 1.95E-04 |
| **10545239** |  |  | Cluster 2 | 6.6 | 6.2 | 6.8 | 9.1 | 9.3 | 9.5 | 6.7 | 1.95E-04 |
| **10545202** | *Gm1077* | predicted gene 1077 | Cluster 2 | 7.4 | 6.9 | 7.1 | 9.8 | 10.0 | 9.8 | 6.7 | 6.86E-05 |
| **10436095** | *Retnla* | resistin like alpha | Cluster 2 | 8.6 | 8.7 | 8.2 | 11.6 | 10.9 | 11.3 | 6.6 | 4.02E-04 |
| **10545196** | *Gm1419|Igkv4-71|Gm1524|Igk-C|Gm10880* | predicted gene 1419 | immunoglobulin kappa chain variable 4-71 | predicted gene 1524 | immunoglobulin kappa chain, constant region | predicted gene 10880 | Cluster 2 | 8.7 | 8.3 | 8.3 | 10.8 | 11.0 | 11.4 | 6.3 | 2.85E-04 |
| **10379535** | *Ccl8* | chemokine (C-C motif) ligand 8 | Cluster 2 | 6.7 | 6.7 | 6.5 | 9.1 | 9.1 | 9.6 | 6.1 | 1.26E-04 |
| **10403015** | *AI324046|IghmAC38.205.12|LOC634541|LOC634206* | expressed sequence AI324046 | Ig mu chain V region AC38 205.12 | ig heavy chain V region 108A-like | Cluster 2 | 7.5 | 7.3 | 7.1 | 9.6 | 9.8 | 10.3 | 6.0 | 3.99E-04 |
| **10545212** | *Gm5574* | immunoglobulin kappa chain variable 12-47 | Cluster 2 | 6.3 | 6.3 | 6.1 | 8.9 | 8.4 | 9.1 | 6.0 | 3.01E-04 |
| **10403063** | *LOC100046275* | ig heavy chain V-II region SESS-like | Cluster 2 | 7.0 | 7.1 | 7.2 | 9.5 | 9.6 | 10.0 | 5.9 | 9.10E-05 |
| **10545194** | *Rprl1|Gm8760|Igkv4-71* | ribonuclease P RNA-like 1 | predicted gene 8760 | immunoglobulin kappa chain variable 4-71 | Cluster 2 | 7.1 | 6.7 | 6.6 | 9.3 | 9.5 | 9.4 | 5.8 | 9.10E-05 |
| **10403021** |  |  | Cluster 2 | 7.0 | 6.9 | 6.8 | 9.2 | 9.4 | 9.8 | 5.6 | 1.58E-04 |
| **10545208** | *Gm189* | predicted gene 189 | Cluster 2 | 7.9 | 6.9 | 7.6 | 9.8 | 9.7 | 10.3 | 5.5 | 2.13E-03 |
| **10545215** | *Igk-V28|Igkv12-46* | immunoglobulin kappa chain variable 28 (V28) | immunoglobulin kappa chain variable 12-46 | Cluster 2 | 6.4 | 7.2 | 6.3 | 8.9 | 8.8 | 9.4 | 5.4 | 2.12E-03 |
| **10403006** | *Gm7112* | predicted gene 7112 | Cluster 2 | 5.9 | 5.9 | 6.0 | 9.1 | 7.5 | 8.5 | 5.4 | 6.51E-03 |
| **10403054** | *LOC435333* | similar to monoclonal antibody heavy chain | Cluster 2 | 8.0 | 7.8 | 7.9 | 10.0 | 10.2 | 10.6 | 5.3 | 2.18E-04 |
| **10545569** | *Reg3g* | regenerating islet-derived 3 gamma | Cluster 2 | 8.5 | 7.9 | 7.5 | 10.6 | 10.1 | 10.5 | 5.3 | 1.77E-03 |
| **10583056** | *Mmp12* | matrix metallopeptidase 12 | Cluster 2 | 8.0 | 8.1 | 8.2 | 10.1 | 10.7 | 10.7 | 5.2 | 3.23E-04 |
| **10403048** | *Ighv1-72|LOC631518* | immunoglobulin heavy variable V1-72 | ig heavy chain V region VH558 B4-like | Cluster 2 | 9.1 | 9.0 | 9.0 | 11.2 | 11.4 | 11.5 | 5.2 | 1.58E-05 |
| **10538903** | *Igk-V28|Gm7202|Igk-V21-4|Igkv6-25|Gm16939* | immunoglobulin kappa chain variable 28 (V28) | predicted gene 7202 | immunoglobulin kappa chain variable 21 (V21)-4 | immunoglobulin kappa chain variable 6-25 | predicted gene, 16939 | Cluster 2 | 7.7 | 7.4 | 7.2 | 9.7 | 9.4 | 10.2 | 5.0 | 1.06E-03 |
| **10403060** | *Ighv1-72|LOC382693|Gm16710* | immunoglobulin heavy variable V1-72 | similar to immunoglobulin heavy chain | predicted gene, 16710 | Cluster 2 | 7.7 | 7.6 | 7.6 | 9.8 | 9.8 | 10.1 | 4.9 | 2.77E-05 |
| **10485357** |  |  | Cluster 2 | 11.3 | 11.1 | 11.0 | 13.2 | 13.7 | 13.1 | 4.6 | 4.31E-04 |
| **10403079** | *LOC435333* | similar to monoclonal antibody heavy chain | Cluster 2 | 7.9 | 7.8 | 7.9 | 9.7 | 10.4 | 10.0 | 4.6 | 4.58E-04 |
| **10523359** | *Cxcl13* | chemokine (C-X-C motif) ligand 13 | Cluster 2 | 10.1 | 9.9 | 9.6 | 11.7 | 12.1 | 12.3 | 4.5 | 6.90E-04 |
| **10545235** |  |  | Cluster 2 | 6.1 | 6.1 | 5.6 | 8.1 | 8.0 | 8.1 | 4.4 | 2.32E-04 |
| **10403028** | *LOC382693|Gm16710* | similar to immunoglobulin heavy chain | predicted gene, 16710 | Cluster 2 | 6.1 | 6.0 | 6.2 | 8.1 | 8.1 | 8.5 | 4.2 | 1.25E-04 |
| **10517165** | *Cd52* | CD52 antigen | Cluster 2 | 8.9 | 8.8 | 8.8 | 10.7 | 10.9 | 11.2 | 4.2 | 1.47E-04 |
| **10501020** | *Chi3l3* | chitinase 3-like 3 | Cluster 2 | 8.7 | 8.6 | 8.8 | 10.7 | 10.5 | 10.8 | 4.0 | 4.86E-05 |
| **10379727** | *Gm11428* | predicted gene 11428 | Cluster 2 | 9.1 | 9.0 | 9.0 | 10.7 | 11.2 | 11.2 | 3.9 | 2.98E-04 |
| **10367532** | *5830405N20Rik* | RIKEN cDNA 5830405N20 gene | Cluster 2 | 7.3 | 6.9 | 7.3 | 8.8 | 9.2 | 9.3 | 3.8 | 6.76E-04 |
| **10349580** | *Pigr* | polymeric immunoglobulin receptor | Cluster 2 | 9.3 | 9.5 | 9.2 | 11.0 | 11.0 | 11.4 | 3.4 | 3.54E-04 |
| **10347915** | *Gm7609|Csprs|Gm7592* | predicted pseudogene 7609 | component of Sp100-rs | predicted gene 7592 | Cluster 2 | 6.8 | 7.1 | 6.8 | 8.7 | 8.8 | 8.6 | 3.4 | 9.89E-05 |
| **10545217** |  |  | Cluster 2 | 5.4 | 5.6 | 5.5 | 8.1 | 6.5 | 7.2 | 3.4 | 1.93E-02 |
| **10347925** | *Gm7609|Csprs|Gm7592* | predicted pseudogene 7609 | component of Sp100-rs | predicted gene 7592 | Cluster 2 | 6.5 | 6.6 | 6.4 | 8.1 | 8.3 | 8.1 | 3.1 | 4.62E-05 |
| **10574213** | *Ccl22* | chemokine (C-C motif) ligand 22 | Cluster 2 | 7.4 | 7.3 | 7.2 | 8.9 | 8.7 | 9.1 | 3.0 | 2.44E-04 |
| **10574226** | *Ccl17* | chemokine (C-C motif) ligand 17 | Cluster 2 | 9.3 | 9.4 | 9.3 | 11.0 | 10.9 | 10.9 | 3.0 | 4.50E-06 |
| **10582879** | *Csprs|Gm7609|Gm7592* | component of Sp100-rs | predicted pseudogene 7609 | predicted gene 7592 | Cluster 2 | 6.4 | 6.7 | 6.4 | 7.9 | 8.3 | 8.0 | 2.9 | 5.58E-04 |
| **10545210** | *Gm1524* | predicted gene 1524 | Cluster 2 | 5.9 | 6.1 | 6.0 | 7.5 | 7.8 | 7.3 | 2.9 | 6.06E-04 |
| **10469278** | *Il2ra* | interleukin 2 receptor, alpha chain | Cluster 2 | 7.9 | 8.0 | 8.0 | 9.3 | 9.5 | 9.6 | 2.8 | 9.12E-05 |
| **10356274** | *Csprs|Gm7609|Gm7592* | component of Sp100-rs | predicted pseudogene 7609 | predicted gene 7592 | Cluster 2 | 6.3 | 6.6 | 6.2 | 7.7 | 8.1 | 7.7 | 2.8 | 1.22E-03 |
| **10395365** | *Agr2* | anterior gradient 2 (Xenopus laevis) | Cluster 2 | 8.1 | 7.9 | 7.6 | 9.6 | 9.2 | 9.3 | 2.8 | 1.35E-03 |
| **10544588** | *Gimap3* | GTPase, IMAP family member 3 | Cluster 2 | 7.6 | 7.7 | 7.3 | 8.8 | 8.8 | 9.2 | 2.7 | 1.46E-03 |
| **10360406** | *Ifi205* | interferon activated gene 205 | Cluster 2 | 8.9 | 8.7 | 8.7 | 10.1 | 10.4 | 10.1 | 2.7 | 2.83E-04 |
| **10460146** |  |  | Cluster 2 | 7.6 | 8.0 | 7.9 | 9.1 | 9.3 | 9.3 | 2.6 | 5.23E-04 |
| **10576034** | *Irf8* | interferon regulatory factor 8 | Cluster 2 | 9.6 | 9.6 | 9.6 | 10.8 | 11.0 | 11.1 | 2.6 | 1.01E-04 |
| **10548892** | *Arhgdib* | Rho, GDP dissociation inhibitor (GDI) beta | Cluster 2 | 10.2 | 10.1 | 10.0 | 11.3 | 11.4 | 11.7 | 2.5 | 5.11E-04 |
| **10455954** | *Gm4951* | predicted gene 4951 | Cluster 2 | 8.2 | 8.1 | 8.2 | 9.3 | 9.5 | 9.6 | 2.4 | 1.60E-04 |
| **10360028** | *Fcgr2b* | Fc receptor, IgG, low affinity IIb | Cluster 2 | 8.8 | 8.7 | 8.8 | 9.8 | 10.1 | 10.1 | 2.4 | 3.05E-04 |
| **10480238** | *St8sia6* | ST8 alpha-N-acetyl-neuraminide alpha-2,8-sialyltransferase 6 | Cluster 2 | 7.7 | 7.6 | 7.3 | 8.6 | 8.8 | 8.9 | 2.3 | 1.16E-03 |
| **10477495** | *U46068* |  | Cluster 2 | 9.8 | 9.2 | 8.9 | 10.8 | 10.2 | 10.5 | 2.3 | 1.92E-02 |
| **10438769** | *Cldn1* | claudin 1 | Cluster 2 | 8.8 | 8.6 | 8.8 | 9.8 | 10.0 | 10.1 | 2.3 | 3.67E-04 |
| **10418848** | *Wdfy4* | WD repeat and FYVE domain containing 4 | Cluster 2 | 8.5 | 8.3 | 8.4 | 9.4 | 9.6 | 9.7 | 2.3 | 3.79E-04 |
| **10548375** | *Clec7a* | C-type lectin domain family 7, member a | Cluster 2 | 9.7 | 9.6 | 9.6 | 10.6 | 10.9 | 10.9 | 2.3 | 3.79E-04 |
| **10385118** | *Dock2* | dedicator of cyto-kinesis 2 | Cluster 2 | 8.9 | 8.8 | 8.8 | 9.8 | 10.1 | 10.1 | 2.3 | 3.79E-04 |
| **10385428** | *Itk* | IL2-inducible T-cell kinase | Cluster 2 | 7.8 | 7.9 | 7.7 | 8.9 | 8.9 | 9.1 | 2.2 | 1.89E-04 |
| **10567366** | *Gp2* | glycoprotein 2 (zymogen granule membrane) | Cluster 2 | 5.9 | 6.2 | 6.1 | 7.3 | 7.1 | 7.4 | 2.2 | 6.52E-04 |
| **10351197** | *Sell* | selectin, lymphocyte | Cluster 2 | 9.5 | 9.4 | 9.2 | 10.4 | 10.5 | 10.6 | 2.2 | 4.24E-04 |
| **10508719** | *Snora16a* | small nucleolar RNA, H/ACA box 16A | Cluster 2 | 9.8 | 9.8 | 9.7 | 10.8 | 11.0 | 10.7 | 2.1 | 3.48E-04 |
| **10538936** |  |  | Cluster 2 | 6.3 | 6.5 | 6.5 | 7.9 | 6.9 | 7.8 | 2.1 | 2.76E-02 |
| **10425726** | *Sept3* | septin 3 | Cluster 2 | 7.8 | 7.8 | 7.7 | 8.7 | 9.0 | 8.8 | 2.0 | 3.48E-04 |
| **10545231** |  |  | Cluster 3 | 7.2 | 6.6 | 7.0 | 11.3 | 12.9 | 9.4 | 18.9 | 1.42E-02 |
| **10545237** |  |  | Cluster 3 | 5.7 | 5.0 | 5.2 | 6.7 | 10.4 | 8.4 | 9.1 | 4.25E-02 |
| **10545233** | *Gm10883* | predicted gene 10883 | Cluster 3 | 5.6 | 5.2 | 5.3 | 7.2 | 9.4 | 7.3 | 6.1 | 2.33E-02 |
| **10582882** |  |  | Cluster 3 | 8.7 | 8.7 | 8.7 | 11.0 | 11.9 | 10.5 | 5.3 | 4.03E-03 |
| **10582888** |  |  | Cluster 3 | 9.0 | 9.0 | 8.6 | 10.9 | 12.0 | 10.7 | 5.1 | 5.39E-03 |
| **10603228** |  |  | Cluster 3 | 6.6 | 6.4 | 6.6 | 8.9 | 9.5 | 7.9 | 4.7 | 9.05E-03 |
| **10582890** |  |  | Cluster 3 | 8.0 | 7.8 | 7.9 | 9.8 | 11.0 | 9.5 | 4.7 | 8.88E-03 |
| **10599092** |  |  | Cluster 3 | 6.6 | 6.6 | 6.6 | 8.8 | 9.6 | 7.8 | 4.5 | 1.49E-02 |
| **10582899** |  |  | Cluster 3 | 8.0 | 7.6 | 7.9 | 9.8 | 10.5 | 9.4 | 4.2 | 3.83E-03 |
| **10598077** |  |  | Cluster 3 | 6.4 | 5.7 | 6.0 | 7.6 | 8.7 | 7.1 | 3.3 | 2.64E-02 |
| **10598229** |  |  | Cluster 3 | 6.3 | 6.5 | 6.5 | 8.3 | 8.8 | 7.3 | 3.3 | 1.89E-02 |
| **10598062** |  |  | Cluster 3 | 8.8 | 7.7 | 8.4 | 10.0 | 10.9 | 9.2 | 3.3 | 4.18E-02 |
| **10598187** |  |  | Cluster 3 | 6.1 | 6.2 | 6.1 | 7.9 | 8.5 | 7.1 | 3.2 | 1.39E-02 |
| **10582916** |  |  | Cluster 3 | 9.1 | 9.3 | 9.0 | 10.4 | 11.6 | 10.0 | 2.9 | 3.49E-02 |
| **10582896** |  |  | Cluster 3 | 9.6 | 9.6 | 9.5 | 10.8 | 12.0 | 10.5 | 2.8 | 2.89E-02 |
| **10403043** | *Ighv1-72* | immunoglobulin heavy variable V1-72 | Cluster 3 | 8.7 | 8.8 | 8.8 | 9.8 | 10.9 | 9.7 | 2.5 | 2.40E-02 |
| **10347218** |  |  | Cluster 3 | 8.9 | 8.2 | 8.6 | 9.7 | 10.6 | 9.5 | 2.5 | 2.57E-02 |
| **10516906** | *Snora73b* | small nucleolar RNA, H/ACA box 73b | Cluster 3 | 9.3 | 9.5 | 9.5 | 10.6 | 11.3 | 10.2 | 2.3 | 1.82E-02 |
| **10516908** | *Snora73a* | small nucleolar RNA, H/ACA box 73a | Cluster 3 | 9.3 | 9.5 | 9.5 | 10.6 | 11.2 | 10.0 | 2.3 | 2.97E-02 |
| **10582884** |  |  | Cluster 3 | 5.6 | 5.5 | 5.5 | 6.3 | 7.3 | 6.2 | 2.1 | 3.90E-02 |
| **10402512** | *Scarna13* | small Cajal body-specific RNA 1 | Cluster 3 | 9.0 | 8.8 | 9.1 | 10.0 | 10.2 | 9.8 | 2.0 | 2.07E-03 |
| **10347222** |  |  | Cluster 3 | 9.8 | 9.6 | 9.4 | 10.4 | 11.3 | 10.2 | 2.0 | 4.45E-02 |
| **10471929** | *Arhgap15* | Rho GTPase activating protein 15 | Cluster 3 | 7.9 | 7.8 | 7.8 | 8.6 | 9.1 | 8.8 | 2.0 | 2.57E-03 |
| **10399360** | *Rhob* | ras homolog gene family, member B | Cluster 4 | 11.3 | 11.2 | 11.1 | 10.3 | 10.1 | 10.3 | -2.0 | 3.94E-04 |
| **10443690** | *Glp1r* | glucagon-like peptide 1 receptor | Cluster 4 | 11.5 | 11.4 | 11.4 | 10.6 | 10.3 | 10.3 | -2.0 | 6.07E-04 |
| **10353450** | *Gm4956* | predicted gene 4956 | Cluster 4 | 9.4 | 9.5 | 9.6 | 8.6 | 8.5 | 8.2 | -2.0 | 1.32E-03 |
| **10425283** | *Maff* | v-maf musculoaponeurotic fibrosarcoma oncogene family, protein F (avian) | Cluster 4 | 9.2 | 9.0 | 9.0 | 8.1 | 8.0 | 8.0 | -2.0 | 1.57E-04 |
| **10450038** | *Angptl4* | angiopoietin-like 4 | Cluster 4 | 9.6 | 9.9 | 9.8 | 9.1 | 8.5 | 8.7 | -2.0 | 7.13E-03 |
| **10352918** | *Mir29c|A330023F24Rik* | microRNA 29c | RIKEN cDNA A330023F24 gene | Cluster 4 | 7.7 | 7.6 | 7.7 | 6.6 | 6.8 | 6.5 | -2.1 | 3.94E-04 |
| **10358515** | *Hmcn1* | hemicentin 1 | Cluster 4 | 9.9 | 10.1 | 10.1 | 9.2 | 9.1 | 8.6 | -2.1 | 5.66E-03 |
| **10407122** |  |  | Cluster 4 | 9.0 | 8.9 | 9.2 | 7.8 | 8.4 | 7.6 | -2.1 | 1.27E-02 |
| **10510574** | *Errfi1* | ERBB receptor feedback inhibitor 1 | Cluster 4 | 12.7 | 12.6 | 12.7 | 11.8 | 11.5 | 11.5 | -2.1 | 5.37E-04 |
| **10426110** | *Pim3* | proviral integration site 3 | Cluster 4 | 10.6 | 10.6 | 10.7 | 9.7 | 9.3 | 9.6 | -2.1 | 9.12E-04 |
| **10462132** | *E030010A14Rik|Pgm5* | RIKEN cDNA E030010A14 gene | phosphoglucomutase 5 | Cluster 4 | 10.3 | 9.9 | 9.9 | 9.1 | 8.8 | 8.9 | -2.2 | 2.34E-03 |
| **10479975** |  |  | Cluster 4 | 9.0 | 8.9 | 8.9 | 7.9 | 8.1 | 7.5 | -2.2 | 3.59E-03 |
| **10396419** |  |  | Cluster 4 | 9.0 | 8.9 | 8.9 | 7.5 | 8.2 | 7.7 | -2.2 | 5.78E-03 |
| **10526410** | *Hspb1|Gm9817* | heat shock protein 1 | predicted gene 9817 | Cluster 4 | 11.9 | 11.8 | 11.7 | 10.7 | 10.4 | 10.8 | -2.2 | 9.40E-04 |
| **10408928** | *Hspb1|Gm9817* | heat shock protein 1 | predicted gene 9817 | Cluster 4 | 12.0 | 11.9 | 11.9 | 10.8 | 10.6 | 11.0 | -2.2 | 7.05E-04 |
| **10449741** | *Sik1* | salt inducible kinase 1 | Cluster 4 | 11.9 | 11.8 | 11.7 | 10.8 | 10.5 | 10.7 | -2.2 | 4.24E-04 |
| **10578950** |  |  | Cluster 4 | 7.4 | 7.0 | 7.2 | 6.1 | 6.1 | 6.0 | -2.2 | 7.05E-04 |
| **10412699** | *Gm10404* | predicted gene 10404 | Cluster 4 | 8.1 | 8.3 | 8.1 | 7.1 | 7.5 | 6.5 | -2.3 | 1.91E-02 |
| **10369290** | *Ddit4* | DNA-damage-inducible transcript 4 | Cluster 4 | 10.3 | 10.1 | 10.2 | 8.8 | 9.0 | 9.1 | -2.3 | 3.05E-04 |
| **10400095** | *Ifrd1|Gm7008* | interferon-related developmental regulator 1 | predicted gene 7008 | Cluster 4 | 10.2 | 10.6 | 10.6 | 9.3 | 9.2 | 9.2 | -2.3 | 8.53E-04 |
| **10578136** |  |  | Cluster 4 | 7.6 | 6.4 | 7.3 | 5.7 | 6.3 | 5.6 | -2.4 | 4.30E-02 |
| **10482772** | *Nr4a2* | nuclear receptor subfamily 4, group A, member 2 | Cluster 4 | 10.4 | 10.0 | 10.1 | 9.0 | 8.8 | 8.8 | -2.4 | 6.97E-04 |
| **10459288** | *Adrb2* | adrenergic receptor, beta 2 | Cluster 4 | 11.7 | 11.8 | 11.8 | 10.6 | 10.7 | 10.3 | -2.4 | 5.87E-04 |
| **10377439** | *Per1* | period homolog 1 (Drosophila) | Cluster 4 | 11.4 | 11.2 | 11.2 | 10.4 | 9.8 | 9.9 | -2.4 | 3.33E-03 |
| **10531610** | *Rasgef1b* | RasGEF domain family, member 1B | Cluster 4 | 10.3 | 10.2 | 10.3 | 9.1 | 8.9 | 9.0 | -2.4 | 4.52E-05 |
| **10575873** | *Osgin1* | oxidative stress induced growth inhibitor 1 | Cluster 4 | 10.0 | 9.9 | 10.2 | 8.8 | 8.6 | 8.9 | -2.5 | 5.29E-04 |
| **10357875** | *Btg2* | B-cell translocation gene 2, anti-proliferative | Cluster 4 | 12.2 | 12.3 | 12.1 | 10.9 | 10.9 | 10.8 | -2.5 | 3.69E-05 |
| **10449284** | *Dusp1* | dual specificity phosphatase 1 | Cluster 4 | 13.5 | 13.4 | 13.2 | 11.9 | 12.1 | 12.0 | -2.5 | 2.04E-04 |
| **10509965** | *Epha2* | Eph receptor A2 | Cluster 4 | 10.5 | 10.4 | 10.4 | 9.2 | 8.9 | 9.0 | -2.5 | 1.20E-04 |
| **10580183** | *Ier2* | immediate early response 2 | Cluster 4 | 9.1 | 9.1 | 8.9 | 7.6 | 7.5 | 7.9 | -2.6 | 5.74E-04 |
| **10495675** | *F3* | coagulation factor III | Cluster 4 | 11.1 | 11.2 | 11.3 | 10.1 | 9.7 | 9.7 | -2.6 | 7.12E-04 |
| **10514466** | *Jun* |  | Cluster 4 | 13.0 | 13.0 | 12.9 | 11.6 | 11.5 | 11.6 | -2.6 | 7.66E-06 |
| **10474972** | *Chac1* | ChaC, cation transport regulator-like 1 (E. coli) | Cluster 4 | 9.4 | 9.8 | 10.3 | 8.5 | 8.4 | 8.5 | -2.6 | 6.48E-03 |
| **10504838** | *Nr4a3* | nuclear receptor subfamily 4, group A, member 3 | Cluster 4 | 9.9 | 9.5 | 9.7 | 8.2 | 8.2 | 8.5 | -2.7 | 7.87E-04 |
| **10597758** | *Csrnp1* | cysteine-serine-rich nuclear protein 1 | Cluster 4 | 10.1 | 9.9 | 9.9 | 8.4 | 8.7 | 8.5 | -2.7 | 2.04E-04 |
| **10543017** | *Pdk4* | pyruvate dehydrogenase kinase, isoenzyme 4 | Cluster 4 | 10.9 | 11.0 | 10.8 | 10.0 | 9.5 | 9.0 | -2.7 | 8.93E-03 |
| **10561453** | *Zfp36* | zinc finger protein 36 | Cluster 4 | 11.6 | 11.6 | 11.1 | 9.8 | 10.0 | 10.2 | -2.7 | 2.11E-03 |
| **10433885** | *Cebpd* | CCAAT/enhancer binding protein (C/EBP), delta | Cluster 4 | 9.6 | 9.5 | 9.0 | 7.7 | 8.0 | 8.0 | -2.8 | 2.24E-03 |
| **10541071** | *8430408G22Rik* | RIKEN cDNA 8430408G22 gene | Cluster 4 | 10.1 | 10.2 | 9.8 | 8.5 | 8.5 | 8.5 | -2.9 | 2.17E-04 |
| **10363735** | *Egr2* | early growth response 2 | Cluster 4 | 10.4 | 10.2 | 10.0 | 8.5 | 8.5 | 8.9 | -2.9 | 8.88E-04 |
| **10434925** | *Hes1* | hairy and enhancer of split 1 (Drosophila) | Cluster 4 | 11.7 | 11.5 | 11.5 | 10.0 | 9.7 | 10.0 | -3.2 | 1.57E-04 |
| **10366346** | *Phlda1* | pleckstrin homology-like domain, family A, member 1 | Cluster 4 | 11.1 | 10.9 | 11.1 | 9.4 | 9.2 | 9.4 | -3.3 | 5.56E-05 |
| **10450367** | *Hspa1a|Hspa1b* | heat shock protein 1A | heat shock protein 1B | Cluster 4 | 13.1 | 13.2 | 13.2 | 11.2 | 11.0 | 11.5 | -3.8 | 2.04E-04 |
| **10444589** | *Hspa1a|Hspa1b* | heat shock protein 1A | heat shock protein 1B | Cluster 4 | 10.1 | 10.0 | 10.0 | 8.2 | 8.2 | 7.9 | -3.8 | 5.20E-05 |
| **10560481** | *Fosb* | FBJ osteosarcoma oncogene B | Cluster 4 | 9.9 | 10.0 | 9.8 | 8.1 | 7.7 | 8.1 | -3.8 | 1.84E-04 |
| **10573198** | *Dnajb1* | DnaJ (Hsp40) homolog, subfamily B, member 1 | Cluster 4 | 11.8 | 11.9 | 11.8 | 9.8 | 9.5 | 9.9 | -4.3 | 7.29E-05 |
| **10450369** | *Hspa1a* | heat shock protein 1A | Cluster 4 | 12.9 | 12.9 | 12.9 | 10.8 | 10.5 | 10.9 | -4.5 | 5.57E-05 |
| **10350516** | *Ptgs2* | prostaglandin-endoperoxide synthase 2 | Cluster 4 | 11.9 | 12.0 | 12.0 | 9.5 | 9.2 | 9.6 | -5.8 | 3.47E-05 |
| **10427035** | *Nr4a1* | nuclear receptor subfamily 4, group A, member 1 | Cluster 4 | 12.1 | 12.0 | 12.0 | 9.0 | 8.9 | 9.6 | -7.5 | 2.04E-04 |
| **10397346** | *Fos* | FBJ osteosarcoma oncogene | Cluster 4 | 11.1 | 11.0 | 10.5 | 7.8 | 7.9 | 8.1 | -7.5 | 1.40E-04 |
| **10454782** | *Egr1* | early growth response 1 | Cluster 4 | 11.8 | 11.6 | 11.5 | 8.5 | 8.4 | 9.0 | -7.8 | 1.28E-04 |
| **10361091** | *Atf3* | activating transcription factor 3 | Cluster 4 | 12.3 | 12.4 | 12.3 | 8.9 | 8.8 | 9.3 | -10.4 | 2.86E-05 |

**Table S3: KEGG pathway enrichment analyses of genes found to be up-regulated (fold change ≥2) in lung tissue from SPC-HAxTCR-HA mice.**

| **KEGG ID** | **GO term** | **% associated genes** | **Term p-value corrected with Bonferroni** |
| --- | --- | --- | --- |
| **4662** | **B cell receptor signaling pathway** | 19,2 | 1,20E-11 |
| **4672** | **Intestinal immune network for IgA production** | 19 | 1,70E-06 |
| **4640** | **Hematopoietic cell lineage** | 14 | 3,10E-08 |
| **4660** | **T cell receptor signaling pathway** | 13,3 | 2,10E-09 |
| **4062** | **Chemokine signaling pathway** | 10,2 | 2,10E-11 |
| **4664** | **Fc epsilon RI signaling pathway** | 10 | 1,00E-03 |
| **4666** | **Fc gamma R-mediated phagocytosis** | 9,1 | 5,60E-04 |
| **4514** | **Cell adhesion molecules (CAMs)** | 8,8 | 5,90E-07 |
| **4612** | **Antigen processing and presentation** | 8,6 | 2,60E-03 |
| **4060** | **Cytokine-cytokine receptor interaction** | 8,3 | 8,00E-11 |
| **4064** | **NF-kappa B signaling pathway** | 7,9 | 1,50E-03 |
| **4610** | **Complement and coagulation cascades** | 7,8 | 1,50E-02 |
| **4650** | **Natural killer cell mediated cytotoxicity** | 6,7 | 4,90E-03 |
| **4670** | **Leukocyte transendothelial migration** | 6,6 | 5,50E-03 |
| **4145** | **Phagosome** | 5,7 | 2,80E-03 |

Depicted are identified terms with p<0.05 (disease-associated terms are not shown).

**Table S4: Significantly enriched gene sets in GSEA.**

| **Name of "hallmark"**  **gene set** | **Size** | **Enrichment**  **score** | **Norm. enrichment score** | **FDR q-value** | **Rank at max.** |
| --- | --- | --- | --- | --- | --- |
| **Allograft rejection** | 174 | 0.8072 | 2.6381 | 0.0% | 1495 |
| **Interferon gamma response** | 180 | 0.7218 | 2.2617 | 0.0% | 2089 |
| **Interferon alpha response** | 84 | 0.7771 | 2.1943 | 0.0% | 2089 |
| **Inflammtory response** | 188 | 0.6510 | 2.0429 | 0.0% | 1349 |
| **IL6 Jak Stat3 signaling** | 81 | 0.7256 | 1.9384 | 0.0% | 2065 |
| **E2F targets** | 182 | 0.5664 | 1.7683 | 0.2% | 3918 |
| **Complement** | 172 | 0.5614 | 1.7588 | 0.3% | 1570 |
| **IL2 Stat5 signaling** | 188 | 0.5600 | 1.7201 | 0.3% | 1666 |
| **G2M checkpoint** | 176 | 0.4980 | 1.5647 | 2.0% | 2916 |
| **Kras signaling up** | 185 | 0.4922 | 1.4831 | 3.8% | 1329 |

Microarray data from SPC-HAxTCR-HA vs. SPC-HA mice (n = 3/group) were analyzed by GSEA tool using 50 curated canonical hallmark gene sets. Results show gene sets with a FDR < 5%.

**Table S5: List of proteins (Ʃ372) identified in BALF from SPC-HA and SPC-HAxTCR-HA mice.** Coverage: percentage of protein covered by matched peptide spectra; area: mean area of the 3 peptides (matched with the respective protein) identified with the highest area of the corresponding peaks in the extracted ion chromatograms; # PSM: number of peptide-spectrum matches; count of peptide spectra (identified by MS/MS) that matched for the respective protein

| **Accession ID** | **Protein** | **Gene symbol** | **Entrez  Gene ID** | **Coverage  SPC-HA** | **Coverage  SPC-HAx**  **TCR-HA** | **Area  SPC-HA** | **Area  SPC-HAx**  **TCR-HA** | **# PSM  SPC-HA** | **# PSM  SPC-HAx**  **TCR-HA** |
| --- | --- | --- | --- | --- | --- | --- | --- | --- | --- |
| **Q64433** | 10 kDa heat shock protein, mitochondrial | *Hspe1* | 15528 | 41.18 | 31.37 | 2.31E+07 | 1.02E+07 | 11 | 10 |
| **Q9CQV8** | 14-3-3 protein beta/alpha | *Ywhab* | 54401 | 34.15 | 22.76 | 6.80E+07 | 4.47E+07 | 49 | 37 |
| **P62259** | 14-3-3 protein epsilon | *Ywhae* | 22627 | 45.1 | 45.1 | 9.49E+07 | 5.27E+07 | 103 | 98 |
| **P68510** | 14-3-3 protein eta | *Ywhah* | 22629 | 35.77 | 32.52 | 7.10E+07 | 4.76E+07 | 52 | 43 |
| **P61982** | 14-3-3 protein gamma | *Ywhag* | 22628 | 34.41 | 27.53 | 7.47E+07 | 4.90E+07 | 57 | 48 |
| **O70456** | 14-3-3 protein sigma | *Sfn* | 55948 | 18.95 | 18.95 | 6.54E+07 | 4.18E+07 | 39 | 33 |
| **P68254** | 14-3-3 protein theta | *Ywhaq* | 22630 | 31.84 | 28.57 | 7.13E+07 | 4.44E+07 | 58 | 44 |
| **P63101** | 14-3-3 protein zeta/delta | *Ywhaz* | 22631 | 49.8 | 49.8 | 1.08E+08 | 7.00E+07 | 97 | 85 |
| **Q99L13** | 3-hydroxyisobutyrate dehydrogenase, mitochondrial | *Hibadh* | 58875 | 8.66 | 4.78 | 4.09E+06 | 4.12E+06 | 3 | 1 |
| **Q8BWT1** | 3-ketoacyl-CoA thiolase, mitochondrial | *Acaa2* | 52538 | 31.49 | 18.39 | 3.72E+07 | 1.19E+07 | 39 | 11 |
| **P62858** | 40S ribosomal protein S28 | *Rps28* | 54127 | 30.43 | 17.39 | 1.20E+07 | 1.04E+07 | 6 | 5 |
| **P63038** | 60 kDa heat shock protein, mitochondrial | *Hspd1* | 15510 | 17.45 | 12.39 | 8.32E+06 | 6.56E+06 | 20 | 9 |
| **P14869** | 60S acidic ribosomal protein P0 | *Rplp0* | 11837 | 16.72 | 20.82 | 8.37E+06 | 6.40E+06 | 9 | 7 |
| **P99027** | 60S acidic ribosomal protein P2 | *Rplp2* | 67186 | 50.43 | 50.43 | 4.34E+07 | 4.42E+07 | 18 | 23 |
| **P53026** | 60S ribosomal protein L10a | *Rpl10a* | 19896 | 9.22 | 9.22 | 7.59E+06 | 5.32E+06 | 6 | 5 |
| **P35979** | 60S ribosomal protein L12 | *Rpl12* | 269261 | 5.45 | 9.7 | 6.91E+06 | 7.02E+06 | 1 | 2 |
| **Q9DCD0** | 6-phosphogluconate dehydrogenase, decarboxylating | *Pgd* | 110208 | 13.87 | 10.35 | 1.38E+07 | 9.39E+06 | 23 | 12 |
| **Q9R1Z7** | 6-pyruvoyl tetrahydrobiopterin synthase | *Pts* | 19286 | 8.33 | 8.33 | 4.26E+06 | 2.81E+06 | 1 | 1 |
| **P20029** | 78 kDa glucose-regulated protein | *Hspa5* | 14828 | 29.92 | 26.41 | 6.95E+07 | 5.01E+07 | 91 | 92 |
| **Q8QZT1** | Acetyl-CoA acetyltransferase, mitochondrial | *Acat1* | 110446 | 8.02 | 2.83 | 5.07E+06 | 3.17E+06 | 5 | 2 |
| **Q99KI0** | Aconitate hydratase, mitochondrial | *Aco2* | 11429 | 5.77 | 2.82 | 6.73E+06 | 4.64E+06 | 6 | 4 |
| **P60710** | Actin, cytoplasmic 1 | *Actb* | 11461 | 23.2 | 23.2 | 6.64E+07 | 5.35E+07 | 57 | 56 |
| **Q9JM76** | Actin-related protein 2/3 complex subunit 3 | *Arpc3* | 56378 | 7.3 | 7.3 | 3.31E+06 | 1.99E+06 | 3 | 3 |
| **P59999** | Actin-related protein 2/3 complex subunit 4 | *Arpc4* | 68089 | 6.55 | 13.1 | 8.00E+06 | 5.98E+06 | 2 | 3 |
| **Q9CPW4** | Actin-related protein 2/3 complex subunit 5 | *Arpc5* | 67771 | 7.95 | 7.95 | 5.71E+06 | 6.18E+06 | 2 | 2 |
| **P11031** | Activated RNA polymerase II transcriptional coactivator p15 | *Sub1* | 20024 | 8.66 | 8.66 | 5.63E+06 | 1.23E+07 | 2 | 5 |
| **O55137** | Acyl-coenzyme A thioesterase 1 | *Acot1* | 26897 | 13.84 | 10.74 | 3.02E+07 | 1.32E+07 | 23 | 12 |
| **Q9QYR9** | Acyl-coenzyme A thioesterase 2, mitochondrial | *Acot2* | 171210 | 16.11 | 9.49 | 2.37E+07 | 1.09E+07 | 27 | 10 |
| **P50247** | Adenosylhomocysteinase | *Ahcy* | 269378 | 13.19 | 5.56 | 9.75E+06 | 5.86E+06 | 10 | 6 |
| **Q9WTP6** | Adenylate kinase 2, mitochondrial | *Ak2* | 11637 | 5.44 | 5.44 | 8.15E+06 | 5.65E+06 | 6 | 6 |
| **P40124** | Adenylyl cyclase-associated protein 1 | *Cap1* | 12331 | 3.8 | 3.8 | 1.19E+07 | 1.11E+07 | 3 | 5 |
| **P61205** | ADP-ribosylation factor 3 | *Arf3* | 11842 | 19.89 | 16.02 | 4.51E+06 | 3.64E+06 | 5 | 5 |
| **Q8QZR5** | Alanine aminotransferase 1 | *Gpt* | 76282 | 11.29 | 3.63 | 1.17E+07 | 3.20E+06 | 10 | 2 |
| **Q9JII6** | Alcohol dehydrogenase [NADP(+)] | *Akr1a1* | 58810 | 10.15 | 4.62 | 7.06E+06 | 7.37E+06 | 5 | 4 |
| **O35945** | Aldehyde dehydrogenase, cytosolic 1 | *Aldh1a7* | 26358 | 6.19 | 5.59 | 5.91E+06 | 4.99E+06 | 7 | 5 |
| **P47738** | Aldehyde dehydrogenase, mitochondrial | *Aldh2* | 11669 | 13.68 | 4.05 | 6.85E+06 | 4.96E+06 | 12 | 3 |
| **G3X982** | Aldehyde oxidase 3 | *Aox3* | 71724 | 7.04 | 1.65 | 1.47E+07 | 5.49E+06 | 22 | 3 |
| **Q8K157** | Aldose 1-epimerase | *Galm* | 319625 | 4.39 | 4.09 | 8.17E+06 | 2.29E+06 | 2 | 1 |
| **P45376** | Aldose reductase | *Akr1b1* | 11677 | 9.49 | 4.43 | 9.22E+06 | 5.46E+06 | 9 | 2 |
| **Q8VCR7** | Alpha/beta hydrolase domain-containing protein 14B | *Abhd14b* | 76491 | 10 | 5.24 | 9.31E+06 | 3.83E+06 | 3 | 1 |
| **Q7TPR4** | Alpha-actinin-1 | *Actn1* | 109711 | 17.49 | 16.03 | 7.05E+07 | 4.18E+07 | 68 | 45 |
| **P57780** | Alpha-actinin-4 | *Actn4* | 60595 | 21.82 | 18.75 | 7.05E+07 | 4.53E+07 | 77 | 56 |
| **P17182** | Alpha-enolase | *Eno1* | 13806 | 12.44 | 9.91 | 2.10E+07 | 1.12E+07 | 15 | 6 |
| **P12023** | Amyloid beta A4 protein | *App* | 11820 | 1.56 | 1.56 | 2.09E+06 | 2.76E+06 | 1 | 3 |
| **P07356** | Annexin A2 | *Anxa2* | 12306 | 9.14 | 3.24 | 3.41E+06 | 3.60E+06 | 4 | 1 |
| **Q9Z0X1** | Apoptosis-inducing factor 1, mitochondrial | *Aifm1* | 26926 | 10.95 | 4.25 | 8.00E+06 | 1.98E+06 | 11 | 3 |
| **P05201** | Aspartate aminotransferase, cytoplasmic | *Got1* | 14718 | 13.8 | 9.69 | 1.16E+07 | 7.30E+06 | 11 | 8 |
| **P05202** | Aspartate aminotransferase, mitochondrial | *Got2* | 14719 | 17.67 | 11.4 | 3.99E+07 | 1.69E+07 | 35 | 15 |
| **Q9Z2W0** | Aspartyl aminopeptidase | *Dnpep* | 13437 | 8.03 | 2.11 | 7.42E+06 | 1.01E+07 | 8 | 4 |
| **Q03265** | ATP synthase subunit alpha, mitochondrial | *Atp5a1* | 11946 | 7.05 | 6.87 | 3.87E+06 | 4.22E+06 | 3 | 5 |
| **P56480** | ATP synthase subunit beta, mitochondrial | *Atp5b* | 11947 | 3.59 | 3.59 | 4.83E+06 | 1.86E+06 | 3 | 1 |
| **Q9D3D9** | ATP synthase subunit delta, mitochondrial | *Atp5d* | 66043 | 8.33 | 8.33 | 8.76E+05 | 6.18E+05 | 1 | 1 |
| **Q91V92** | ATP-citrate synthase | *Acly* | 104112 | 9.9 | 7.33 | 1.17E+07 | 5.93E+06 | 20 | 10 |
| **Q9R069** | Basal cell adhesion molecule | *Bcam* | 57278 | 2.25 | 4.5 | 9.65E+06 | 3.05E+06 | 2 | 3 |
| **P09803** | Cadherin-1 | *Cdh1* | 12550 | 5.54 | 2.49 | 4.09E+06 | 3.80E+06 | 6 | 4 |
| **P62204** | Calmodulin | *Calm1* | 12315 | 14.09 | 14.09 | 2.03E+07 | 1.55E+07 | 10 | 12 |
| **O35887** | Calumenin | *Calu* | 12321 | 7.3 | 7.62 | 4.13E+06 | 3.96E+06 | 7 | 5 |
| **P28651** | Carbonic anhydrase-related protein | *Ca8* | 12319 | 7.22 | 7.22 | 1.70E+07 | 1.08E+07 | 7 | 4 |
| **P08074** | Carbonyl reductase [NADPH] 2 | *Cbr2* | 12409 | 31.97 | 28.69 | 1.73E+08 | 1.15E+08 | 72 | 74 |
| **Q8VCT4** | Carboxylesterase 1D | *Ces1d* | 104158 | 19.65 | 10.97 | 7.80E+07 | 3.11E+07 | 72 | 46 |
| **P24270** | Catalase | *Cat* | 12359 | 6.07 | 4.55 | 5.21E+06 | 2.58E+06 | 9 | 6 |
| **P26231** | Catenin alpha-1 | *Ctnna1* | 12385 | 1.32 | 1.32 | 2.79E+06 | 1.72E+06 | 2 | 1 |
| **P10605** | Cathepsin B | *Ctsb* | 13030 | 10.32 | 7.08 | 9.33E+06 | 5.87E+06 | 10 | 5 |
| **P18242** | Cathepsin D | *Ctsd* | 13033 | 3.66 | 8.54 | 3.07E+06 | 9.49E+06 | 2 | 10 |
| **Q61490** | CD166 antigen | *Alcam* | 11658 | 8.58 | 4.97 | 9.27E+06 | 2.27E+06 | 8 | 3 |
| **Q61362** | Chitinase-3-like protein 1 | *Chi3l1* | 12654 | 2.57 | 12.34 | 1.14E+06 | 1.84E+07 | 2 | 21 |
| **Q9Z1Q5** | Chloride intracellular channel protein 1 | *Clic1* | 114584 | 12.45 | 23.65 | 7.04E+06 | 5.82E+06 | 6 | 7 |
| **Q9QYB1** | Chloride intracellular channel protein 4 | *Clic4* | 29876 | 13.04 | 4.35 | 9.10E+06 | 1.11E+07 | 4 | 4 |
| **P23198** | Chromobox protein homolog 3 | *Cbx3* | 12417 | 13.11 | 13.11 | 3.66E+06 | 3.50E+06 | 3 | 4 |
| **Q06890** | Clusterin | *Clu* | 12759 | 14.06 | 10.71 | 2.31E+07 | 1.13E+07 | 24 | 17 |
| **Q5XJY5** | Coatomer subunit delta | *Arcn1* | 213827 | 6.46 | 4.11 | 3.21E+06 | 1.68E+06 | 6 | 4 |
| **Q9QZE5** | Coatomer subunit gamma-1 | *Copg1* | 54161 | 1.72 | 1.72 | 1.19E+06 | 1.63E+06 | 1 | 1 |
| **P18760** | Cofilin-1 | *Cfl1* | 12631 | 18.67 | 27.11 | 3.17E+07 | 2.38E+07 | 16 | 18 |
| **P45591** | Cofilin-2 | *Cfl2* | 12632 | 18.67 | 6.63 | 1.47E+07 | 3.44E+07 | 10 | 4 |
| **P01027** | Complement C3 | *C3* | 12266 | 2.29 | 4.93 | 7.79E+06 | 1.42E+07 | 7 | 22 |
| **O35658** | Complement component 1 Q subcomponent-binding protein, mitochondrial | *C1qbp* | 12261 | 3.96 | 3.96 | 5.63E+06 | 9.44E+05 | 4 | 1 |
| **O08997** | Copper transport protein ATOX1 | *Atox1* | 11927 | 19.12 | 19.12 | 1.35E+07 | 6.54E+06 | 5 | 2 |
| **Q8CCK0** | Core histone macro-H2A.2 | *H2afy2* | 404634 | 2.42 | 2.42 | 3.49E+06 | 3.13E+06 | 1 | 1 |
| **P30275** | Creatine kinase U-type, mitochondrial | *Ckmt1* | 12716 | 7.42 | 2.39 | 4.43E+06 | 2.85E+06 | 3 | 1 |
| **Q6ZQ38** | Cullin-associated NEDD8-dissociated protein 1 | *Cand1* | 71902 | 0.98 | 1.14 | 2.37E+06 | 1.26E+06 | 1 | 1 |
| **P21460** | Cystatin-C | *Cst3* | 13010 | 19.29 | 19.29 | 9.64E+06 | 8.32E+06 | 9 | 12 |
| **P97315** | Cysteine and glycine-rich protein 1 | *Csrp1* | 13007 | 12.95 | 5.18 | 5.06E+06 | 4.18E+06 | 6 | 4 |
| **Q9DCT8** | Cysteine-rich protein 2 | *Crip2* | 68337 | 6.73 | 6.73 | 6.14E+06 | 2.70E+06 | 2 | 1 |
| **P62897** | Cytochrome c, somatic | *Cycs* | 13063 | 20.95 | 20.95 | 2.54E+07 | 1.33E+07 | 9 | 9 |
| **O88487** | Cytoplasmic dynein 1 intermediate chain 2 | *Dync1i2* | 13427 | 2.29 | 2.29 | 3.58E+06 | 3.67E+06 | 1 | 2 |
| **Q9CPY7** | Cytosol aminopeptidase | *Lap3* | 66988 | 11.18 | 12.52 | 8.18E+06 | 1.16E+07 | 10 | 18 |
| **Q91V12** | Cytosolic acyl coenzyme A thioester hydrolase | *Acot7* | 70025 | 9.19 | 9.19 | 8.36E+06 | 5.97E+06 | 10 | 11 |
| **Q9D1A2** | Cytosolic non-specific dipeptidase | *Cndp2* | 66054 | 24.84 | 18.53 | 2.34E+07 | 1.24E+07 | 30 | 17 |
| **O35215** | D-dopachrome decarboxylase | *Ddt* | 13202 | 16.95 | 16.95 | 2.35E+07 | 1.01E+07 | 13 | 4 |
| **P10518** | Delta-aminolevulinic acid dehydratase | *Alad* | 17025 | 15.76 | 15.76 | 3.32E+07 | 2.06E+07 | 26 | 29 |
| **Q9R0P5** | Destrin | *Dstn* | 56431 | 24.85 | 24.85 | 1.98E+07 | 1.39E+07 | 29 | 19 |
| **O08749** | Dihydrolipoyl dehydrogenase, mitochondrial | *Dld* | 13382 | 2.36 | 4.52 | 4.93E+06 | 1.96E+06 | 3 | 4 |
| **O08553** | Dihydropyrimidinase-related protein 2 | *Dpysl2* | 12934 | 12.41 | 3.67 | 8.19E+06 | 3.53E+06 | 17 | 4 |
| **P97821** | Dipeptidyl peptidase 1 | *Ctsc* | 13032 | 2.6 | 2.6 | 7.07E+06 | 1.70E+07 | 2 | 6 |
| **Q99KK7** | Dipeptidyl peptidase 3 | *Dpp3* | 75221 | 3.79 | 2.44 | 5.48E+06 | 7.23E+06 | 3 | 2 |
| **P28843** | Dipeptidyl peptidase 4 | *Dpp4* | 13482 | 3.16 | 3.16 | 4.94E+06 | 2.42E+06 | 4 | 3 |
| **Q99KJ8** | Dynactin subunit 2 | *Dctn2* | 69654 | 4.73 | 6.97 | 5.40E+06 | 3.75E+06 | 2 | 5 |
| **Q9D8Y0** | EF-hand domain-containing protein D2 | *Efhd2* | 27984 | 15.42 | 14.58 | 3.89E+06 | 4.38E+06 | 6 | 6 |
| **P70372** | ELAV-like protein 1 | *Elavl1* | 15568 | 10.74 | 7.36 | 1.79E+06 | 3.00E+06 | 4 | 4 |
| **P10126** | Elongation factor 1-alpha 1 | *Eef1a1* | 13627 | 5.84 | 7.79 | 1.93E+07 | 1.97E+07 | 10 | 18 |
| **O70251** | Elongation factor 1-beta | *Eef1b* | 55949 | 14.67 | 14.67 | 1.28E+07 | 8.83E+06 | 13 | 14 |
| **P57776** | Elongation factor 1-delta | *Eef1d* | 66656 | 8.54 | 12.81 | 1.34E+07 | 8.28E+06 | 6 | 8 |
| **Q9D8N0** | Elongation factor 1-gamma | *Eef1g* | 67160 | 2.97 | 2.97 | 1.21E+07 | 1.23E+07 | 5 | 2 |
| **P58252** | Elongation factor 2 | *Eef2* | 13629 | 6.88 | 4.43 | 1.56E+07 | 1.26E+07 | 15 | 15 |
| **P57759** | Endoplasmic reticulum resident protein 29 | *Erp29* | 67397 | 4.58 | 4.58 | 6.89E+06 | 3.81E+06 | 2 | 2 |
| **P08113** | Endoplasmin | *Hsp90b1* | 22027 | 14.59 | 16.83 | 3.84E+07 | 4.89E+07 | 31 | 42 |
| **P42125** | Enoyl-CoA delta isomerase 1, mitochondrial | *Eci1* | 13177 | 25.95 | 14.88 | 4.06E+07 | 1.12E+07 | 33 | 16 |
| **Q6ZWX6** | Eukaryotic translation initiation factor 2 subunit 1 | *Eif2s1* | 13665 | 3.81 | 3.81 | 2.72E+06 | 3.41E+06 | 2 | 2 |
| **Q99L45** | Eukaryotic translation initiation factor 2 subunit 2 | *Eif2s2* | 67204 | 8.16 | 3.63 | 3.99E+06 | 4.29E+06 | 3 | 2 |
| **Q9ERK4** | Exportin-2 | *Cse1l* | 110750 | 0.82 | 0.82 | 5.34E+08 | 2.82E+08 | 1 | 5 |
| **P26040** | Ezrin | *Ezr* | 22350 | 17.58 | 10.41 | 3.54E+07 | 1.82E+07 | 38 | 21 |
| **P47754** | F-actin-capping protein subunit alpha-2 | *Capza2* | 12343 | 8.74 | 8.74 | 5.25E+06 | 3.95E+06 | 4 | 2 |
| **P47757** | F-actin-capping protein subunit beta | *Capzb* | 12345 | 3.61 | 3.61 | 5.36E+06 | 3.80E+06 | 2 | 1 |
| **Q3U0V1** | Far upstream element-binding protein 2 | *Khsrp* | 16549 | 1.74 | 3.21 | 3.54E+06 | 2.56E+06 | 2 | 2 |
| **Q920E5** | Farnesyl pyrophosphate synthase | *Fdps* | 110196 | 7.37 | 7.37 | 2.11E+07 | 7.20E+06 | 15 | 4 |
| **P19096** | Fatty acid synthase | *Fasn* | 14104 | 8.55 | 6.75 | 2.44E+07 | 1.92E+07 | 49 | 45 |
| **Q05816** | Fatty acid-binding protein, epidermal | *Fabp5* | 16592 | 13.33 | 13.33 | 2.57E+07 | 8.47E+06 | 9 | 6 |
| **P09528** | Ferritin heavy chain | *Fth1* | 14319 | 12.09 | 12.09 | 3.18E+07 | 1.89E+07 | 11 | 8 |
| **P29391** | Ferritin light chain 1 | *Ftl1* | 14325 | 31.15 | 18.58 | 3.12E+07 | 1.37E+07 | 46 | 25 |
| **Q8BTM8** | Filamin-A | *Flna* | 192176 | 2.95 | 2.34 | 5.59E+06 | 4.39E+06 | 9 | 7 |
| **Q80X90** | Filamin-B | *Flnb* | 286940 | 1.96 | 1.04 | 4.14E+06 | 2.39E+06 | 5 | 2 |
| **P70695** | Fructose-1,6-bisphosphatase isozyme 2 | *Fbp2* | 14120 | 12.68 | 10.03 | 9.55E+06 | 5.97E+06 | 13 | 4 |
| **P05064** | Fructose-bisphosphate aldolase A | *Aldoa* | 11674 | 22.53 | 16.21 | 2.44E+07 | 2.11E+07 | 24 | 26 |
| **P97807** | Fumarate hydratase, mitochondrial | *Fh* | 14194 | 17.75 | 11.64 | 1.42E+07 | 6.04E+06 | 21 | 10 |
| **P35505** | Fumarylacetoacetase | *Fah* | 14085 | 8.11 | 8.11 | 1.44E+07 | 6.65E+06 | 10 | 8 |
| **P23591** | GDP-L-fucose synthase | *Tsta3* | 22122 | 4.98 | 3.74 | 2.81E+06 | 1.65E+06 | 1 | 1 |
| **P13020** | Gelsolin | *Gsn* | 227753 | 4.87 | 4.87 | 8.78E+06 | 3.29E+06 | 8 | 5 |
| **P06745** | Glucose-6-phosphate isomerase | *Gpi* | 14751 | 7.35 | 5.73 | 2.66E+07 | 1.99E+07 | 15 | 12 |
| **O08795** | Glucosidase 2 subunit beta | *Prkcsh* | 19089 | 6.33 | 1.92 | 4.91E+06 | 5.57E+06 | 4 | 2 |
| **P26443** | Glutamate dehydrogenase 1, mitochondrial | *Glud1* | 14661 | 9.68 | 4.84 | 4.68E+06 | 2.62E+06 | 6 | 3 |
| **P97494** | Glutamate--cysteine ligase catalytic subunit | *Gclc* | 14629 | 14.76 | 11.77 | 2.55E+07 | 1.24E+07 | 21 | 13 |
| **Q9QUH0** | Glutaredoxin-1 | *Glrx* | 93692 | 10.28 | 10.28 | 3.56E+07 | 1.18E+07 | 4 | 4 |
| **P11352** | Glutathione peroxidase 1 | *Gpx1* | 14775 | 23.38 | 18.41 | 7.61E+06 | 8.89E+06 | 10 | 12 |
| **P47791** | Glutathione reductase, mitochondrial | *Gsr* | 14782 | 4.6 | 7.8 | 1.81E+07 | 8.67E+06 | 8 | 10 |
| **P24472** | Glutathione S-transferase A4 | *Gsta4* | 14860 | 3.6 | 3.6 | 2.83E+07 | 2.13E+07 | 3 | 3 |
| **P10649** | Glutathione S-transferase Mu 1 | *Gstm1* | 14862 | 28.44 | 24.31 | 7.55E+07 | 2.60E+07 | 60 | 42 |
| **P15626** | Glutathione S-transferase Mu 2 | *Gstm2* | 14863 | 24.31 | 15.14 | 5.71E+07 | 2.41E+07 | 34 | 22 |
| **P19157** | Glutathione S-transferase P 1 | *Gstp1* | 14870 | 7.62 | 7.62 | 3.79E+06 | 4.99E+06 | 4 | 5 |
| **P51855** | Glutathione synthetase | *Gss* | 14854 | 1.9 | 1.9 | 1.50E+06 | 1.01E+06 | 1 | 1 |
| **Q8CI94** | Glycogen phosphorylase, brain form | *Pygb* | 110078 | 1.42 | 1.42 | 3.88E+06 | 1.01E+06 | 3 | 2 |
| **Q9ET01** | Glycogen phosphorylase, liver form | *Pygl* | 110095 | 1.41 | 3.53 | 4.90E+06 | 3.98E+06 | 2 | 3 |
| **Q9CPV4** | Glyoxalase domain-containing protein 4 | *Glod4* | 67201 | 11.74 | 11.74 | 1.29E+07 | 8.79E+06 | 8 | 9 |
| **Q99JX3** | Golgi reassembly-stacking protein 2 | *Gorasp2* | 70231 | 3.55 | 3.55 | 4.53E+06 | 4.48E+06 | 1 | 1 |
| **Q99LP6** | GrpE protein homolog 1, mitochondrial | *Grpel1* | 17713 | 5.07 | 5.07 | 3.33E+06 | 1.69E+06 | 2 | 2 |
| **Q9WTP7** | GTP:AMP phosphotransferase AK3, mitochondrial | *Ak3* | 56248 | 12.33 | 6.61 | 1.14E+07 | 5.22E+06 | 4 | 1 |
| **P01921** | H-2 class II histocompatibility antigen, A-D beta chain | *H2-Ab1* | 14961 | 13.96 | 6.04 | 3.28E+06 | 3.69E+06 | 4 | 2 |
| **P04227** | H-2 class II histocompatibility antigen, A-Q alpha chain (Fragment) | *H2-Aa* | 14960 | 6.79 | 6.79 | 1.22E+07 | 8.84E+06 | 3 | 2 |
| **Q9CYW4** | Haloacid dehalogenase-like hydrolase domain-containing protein 3 | *Hdhd3* | 72748 | 4.38 | 4.38 | 3.24E+06 | 6.75E+05 | 3 | 1 |
| **Q61696** | Heat shock 70 kDa protein 1A | *Hspa1a* | 193740 | 19.5 | 13.88 | 2.05E+07 | 1.97E+07 | 37 | 26 |
| **Q61316** | Heat shock 70 kDa protein 4 | *Hspa4* | 15525 | 4.99 | 2.85 | 4.76E+06 | 3.84E+06 | 5 | 2 |
| **P63017** | Heat shock cognate 71 kDa protein | *Hspa8* | 15481 | 25.54 | 30.19 | 4.72E+07 | 3.57E+07 | 69 | 66 |
| **Q9CQN1** | Heat shock protein 75 kDa, mitochondrial | *Trap1* | 68015 | 3.68 | 3.68 | 1.27E+08 | 6.42E+07 | 10 | 12 |
| **P07901** | Heat shock protein HSP 90-alpha | *Hsp90aa1* | 15519 | 22.78 | 21.56 | 7.14E+07 | 7.45E+07 | 64 | 78 |
| **P11499** | Heat shock protein HSP 90-beta | *Hsp90ab1* | 15516 | 24.03 | 21.55 | 8.20E+07 | 9.09E+07 | 57 | 76 |
| **P17156** | Heat shock-related 70 kDa protein 2 | *Hspa2* | 15512 | 12.64 | 12.64 | 4.63E+07 | 3.15E+07 | 33 | 33 |
| **P51859** | Hepatoma-derived growth factor | *Hdgf* | 15191 | 15.19 | 4.22 | 4.39E+06 | 1.27E+06 | 7 | 2 |
| **Q99020** | Heterogeneous nuclear ribonucleoprotein A/B | *Hnrnpab* | 15384 | 4.91 | 4.91 | 2.86E+07 | 1.66E+07 | 5 | 5 |
| **P49312** | Heterogeneous nuclear ribonucleoprotein A1 | *Hnrnpa1* | 15382 | 3.13 | 3.13 | 8.06E+06 | 4.19E+06 | 5 | 5 |
| **Q8BG05** | Heterogeneous nuclear ribonucleoprotein A3 | *Hnrnpa3* | 229279 | 5.01 | 5.01 | 7.29E+06 | 6.51E+06 | 10 | 8 |
| **Q60668** | Heterogeneous nuclear ribonucleoprotein D0 | *Hnrnpd* | 11991 | 3.94 | 3.94 | 2.44E+07 | 2.06E+07 | 5 | 4 |
| **O35737** | Heterogeneous nuclear ribonucleoprotein H | *Hnrnph1* | 59013 | 3.79 | 3.79 | 4.10E+06 | 1.61E+06 | 2 | 2 |
| **P61979** | Heterogeneous nuclear ribonucleoprotein K | *Hnrnpk* | 15387 | 21.6 | 21.6 | 1.53E+07 | 8.62E+06 | 28 | 18 |
| **Q7TMK9** | Heterogeneous nuclear ribonucleoprotein Q | *Syncrip* | 56403 | 4.49 | 2.09 | 2.36E+06 | 2.42E+06 | 3 | 2 |
| **P17710** | Hexokinase-1 | *Hk1* | 15275 | 2.26 | 1.33 | 4.67E+06 | 4.27E+06 | 4 | 2 |
| **O54879** | High mobility group protein B3 | *Hmgb3* | 15354 | 6.5 | 6.5 | 6.80E+06 | 5.04E+06 | 2 | 2 |
| **P70349** | Histidine triad nucleotide-binding protein 1 | *Hint1* | 15254 | 11.11 | 11.11 | 9.92E+06 | 7.43E+06 | 2 | 2 |
| **P15864** | Histone H1.2 | *Hist1h1c* | 50708 | 15.09 | 15.09 | 1.49E+07 | 3.03E+07 | 12 | 23 |
| **P43274** | Histone H1.4 | *Hist1h1e* | 50709 | 14.61 | 14.61 | 1.39E+07 | 2.61E+07 | 13 | 23 |
| **Q8CGP6** | Histone H2A type 1-H | *Hist1h2ah* | 319168 | 21.88 | 21.88 | 2.19E+08 | 1.56E+08 | 37 | 35 |
| **P0C0S6** | Histone H2A.Z | *H2afz* | 51788 | 14.84 | 14.84 | 1.56E+08 | 1.48E+08 | 14 | 25 |
| **P10853** | Histone H2B type 1-F/J/L | *Hist1h2bf* | 665622 | 19.05 | 7.14 | 2.68E+08 | 2.82E+08 | 17 | 17 |
| **P02301** | Histone H3.3C | *H3f3c* | 625328 | 5.15 | 10.29 | 1.01E+08 | 7.35E+07 | 9 | 29 |
| **P62806** | Histone H4 | *Hist1h4a* | 320332 | 38.83 | 29.13 | 2.56E+08 | 2.52E+08 | 74 | 97 |
| **Q99L47** | Hsc70-interacting protein | *St13* | 70356 | 6.47 | 9.97 | 5.60E+06 | 4.37E+06 | 3 | 6 |
| **P00493** | Hypoxanthine-guanine phosphoribosyltransferase | *Hprt1* | 15452 | 5.96 | 11.93 | 3.50E+06 | 5.50E+06 | 3 | 5 |
| **Q91VM9** | Inorganic pyrophosphatase 2, mitochondrial | *Ppa2* | 74776 | 7.88 | 3.94 | 6.55E+06 | 3.61E+06 | 3 | 1 |
| **O55023** | Inositol monophosphatase 1 | *Impa1* | 55980 | 12.64 | 12.64 | 8.74E+06 | 6.39E+06 | 11 | 8 |
| **O88844** | Isocitrate dehydrogenase [NADP] cytoplasmic | *Idh1* | 15926 | 2.9 | 2.17 | 3.91E+06 | 2.32E+06 | 1 | 1 |
| **P58044** | Isopentenyl-diphosphate Delta-isomerase 1 | *Idi1* | 319554 | 14.1 | 5.29 | 9.01E+06 | 6.93E+06 | 8 | 2 |
| **P02535** | Keratin, type I cytoskeletal 10 | *Krt10* | 16661 | 11.93 | 9.12 | 2.20E+07 | 7.63E+06 | 28 | 15 |
| **Q9Z2K1** | Keratin, type I cytoskeletal 16 | *Krt16* | 16666 | 7.68 | 3.41 | 1.13E+07 | 6.52E+06 | 14 | 6 |
| **Q9QWL7** | Keratin, type I cytoskeletal 17 | *Krt17* | 16667 | 13.16 | 7.39 | 1.89E+07 | 8.12E+06 | 16 | 11 |
| **P05784** | Keratin, type I cytoskeletal 18 | *Krt18* | 16668 | 23.64 | 20.09 | 2.38E+07 | 1.58E+07 | 27 | 21 |
| **P19001** | Keratin, type I cytoskeletal 19 | *Krt19* | 16669 | 28.78 | 28.78 | 2.35E+07 | 1.25E+07 | 32 | 33 |
| **Q6IFX2** | Keratin, type I cytoskeletal 42 | *Krt42* | 68239 | 8.19 | 5.53 | 1.89E+07 | 8.12E+06 | 14 | 10 |
| **P04104** | Keratin, type II cytoskeletal 1 | *Krt1* | 16678 | 6.28 | 3.61 | 3.82E+07 | 1.45E+07 | 16 | 9 |
| **Q3UV17** | Keratin, type II cytoskeletal 2 oral | *Krt76* | 77055 | 4.21 | 1.52 | 8.64E+06 | 3.64E+06 | 4 | 1 |
| **P50446** | Keratin, type II cytoskeletal 6A | *Krt6a* | 16687 | 10.67 | 3.8 | 3.10E+07 | 6.24E+06 | 14 | 7 |
| **Q9DCV7** | Keratin, type II cytoskeletal 7 | *Krt7* | 110310 | 15.32 | 9.41 | 1.31E+07 | 4.56E+06 | 18 | 6 |
| **Q8VED5** | Keratin, type II cytoskeletal 79 | *Krt79* | 223917 | 3.58 | 2.26 | 2.56E+07 | 3.92E+06 | 7 | 3 |
| **P11679** | Keratin, type II cytoskeletal 8 | *Krt8* | 16691 | 33.88 | 34.49 | 3.03E+07 | 1.58E+07 | 70 | 47 |
| **Q9CPU0** | Lactoylglutathione lyase | *Glo1* | 109801 | 10.33 | 10.33 | 1.29E+07 | 6.87E+06 | 13 | 7 |
| **Q61029** | Lamina-associated polypeptide 2, isoforms beta/delta/epsilon/gamma | *Tmpo* | 21917 | 14.16 | 9.96 | 8.70E+06 | 5.65E+06 | 12 | 8 |
| **P14733** | Lamin-B1 | *Lmnb1* | 16906 | 12.59 | 12.41 | 1.38E+07 | 9.94E+06 | 18 | 16 |
| **P21619** | Lamin-B2 | *Lmnb2* | 16907 | 11.24 | 2.01 | 5.12E+06 | 3.86E+06 | 12 | 2 |
| **Q61792** | LIM and SH3 domain protein 1 | *Lasp1* | 16796 | 9.51 | 5.7 | 5.03E+06 | 6.31E+06 | 4 | 3 |
| **P06151** | L-lactate dehydrogenase A chain | *Ldha* | 16828 | 22.89 | 22.89 | 1.14E+08 | 7.81E+07 | 70 | 75 |
| **P16125** | L-lactate dehydrogenase B chain | *Ldhb* | 16832 | 20.96 | 20.96 | 7.61E+07 | 3.79E+07 | 50 | 38 |
| **P51174** | Long-chain specific acyl-CoA dehydrogenase, mitochondrial | *Acadl* | 11363 | 11.4 | 2.79 | 1.56E+07 | 1.07E+07 | 16 | 4 |
| **Q91X52** | L-xylulose reductase | *Dcxr* | 67880 | 9.02 | 4.51 | 2.06E+06 | 2.53E+06 | 2 | 2 |
| **Q99MN1** | Lysine--tRNA ligase | *Kars* | 85305 | 2.18 | 3.03 | 1.37E+07 | 1.77E+06 | 2 | 2 |
| **P11438** | Lysosome-associated membrane glycoprotein 1 | *Lamp1* | 16783 | 5.67 | 2.96 | 2.89E+06 | 4.55E+06 | 7 | 4 |
| **P08905** | Lysozyme C-2 | *Lyz2* | 17105 | 10.14 | 14.86 | 2.29E+07 | 9.53E+07 | 5 | 4 |
| **Q9DAR7** | m7GpppX diphosphatase | *Dcps* | 69305 | 8.88 | 3.25 | 1.03E+07 | 4.62E+06 | 10 | 2 |
| **P24452** | Macrophage-capping protein | *Capg* | 12332 | 7.1 | 7.1 | 5.55E+06 | 4.05E+06 | 3 | 3 |
| **P14152** | Malate dehydrogenase, cytoplasmic | *Mdh1* | 17449 | 16.17 | 16.17 | 3.43E+07 | 1.53E+07 | 24 | 20 |
| **P08249** | Malate dehydrogenase, mitochondrial | *Mdh2* | 17448 | 29.88 | 20.71 | 5.78E+07 | 1.70E+07 | 84 | 34 |
| **Q9CXI5** | Mesencephalic astrocyte-derived neurotrophic factor | *Manf* | 74840 | 8.38 | 8.38 | 1.17E+07 | 8.20E+06 | 4 | 2 |
| **P12032** | Metalloproteinase inhibitor 1 | *Timp1* | 21857 | 4.39 | 4.39 | 1.02E+07 | 2.60E+06 | 3 | 1 |
| **P26041** | Moesin | *Msn* | 17698 | 18.2 | 11.96 | 2.41E+07 | 1.43E+07 | 42 | 30 |
| **Q02496** | Mucin-1 | *Muc1* | 17829 | 2.06 | 2.06 | 5.24E+06 | 4.30E+06 | 1 | 1 |
| **Q60605** | Myosin light polypeptide 6 | *Myl6* | 17904 | 30.46 | 17.22 | 3.96E+07 | 2.70E+07 | 19 | 16 |
| **Q3THE2** | Myosin regulatory light chain 12B | *Myl12b* | 67938 | 23.84 | 12.79 | 1.64E+07 | 1.36E+07 | 14 | 11 |
| **Q6URW6** | Myosin-14 | *Myh14* | 71960 | 6.6 | 5.15 | 8.10E+06 | 4.02E+06 | 18 | 16 |
| **Q8VDD5** | Myosin-9 | *Myh9* | 17886 | 1.38 | 5 | 4.74E+06 | 6.48E+06 | 5 | 19 |
| **P62774** | Myotrophin | *Mtpn* | 14489 | 14.41 | 14.41 | 2.17E+07 | 1.27E+07 | 5 | 5 |
| **P70441** | Na(+)/H(+) exchange regulatory cofactor NHE-RF1 | *Slc9a3r1* | 26941 | 7.04 | 7.04 | 3.45E+06 | 3.62E+06 | 3 | 3 |
| **Q8K4Z3** | NAD(P)H-hydrate epimerase | *Apoa1bp* | 246703 | 10.64 | 6.38 | 1.83E+07 | 5.71E+06 | 12 | 3 |
| **Q9D6J6** | NADH dehydrogenase [ubiquinone] flavoprotein 2, mitochondrial | *Ndufv2* | 72900 | 4.03 | 4.03 | 1.44E+06 | 4.27E+05 | 1 | 1 |
| **Q9DCN2** | NADH-cytochrome b5 reductase 3 | *Cyb5r3* | 109754 | 4.32 | 4.32 | 5.52E+06 | 3.92E+06 | 1 | 2 |
| **P06801** | NADP-dependent malic enzyme | *Me1* | 17436 | 13.46 | 5.77 | 1.91E+07 | 8.55E+06 | 20 | 9 |
| **Q60817** | Nascent polypeptide-associated complex subunit alpha | *Naca* | 17938 | 13.49 | 13.95 | 5.21E+06 | 2.79E+06 | 4 | 6 |
| **Q99KQ4** | Nicotinamide phosphoribosyltransferase | *Nampt* | 59027 | 5.3 | 2.65 | 3.41E+06 | 4.69E+06 | 2 | 1 |
| **Q9CZ44** | NSFL1 cofactor p47 | *Nsfl1c* | 386649 | 13.51 | 7.57 | 4.48E+06 | 3.06E+06 | 7 | 2 |
| **O35685** | Nuclear migration protein nudC | *Nudc* | 18221 | 11.45 | 9.34 | 6.93E+06 | 3.79E+06 | 5 | 6 |
| **P62960** | Nuclease-sensitive element-binding protein 1 | *Ybx1* | 22608 | 12.11 | 17.39 | 1.45E+06 | 4.70E+06 | 3 | 8 |
| **P81117** | Nucleobindin-2 | *Nucb2* | 53322 | 4.76 | 2.62 | 2.59E+06 | 2.60E+06 | 4 | 3 |
| **P09405** | Nucleolin | *Ncl* | 17975 | 5.94 | 5.52 | 4.73E+06 | 7.54E+06 | 6 | 11 |
| **P15532** | Nucleoside diphosphate kinase A | *Nme1* | 18102 | 11.84 | 11.84 | 2.56E+07 | 2.22E+07 | 6 | 8 |
| **P29758** | Ornithine aminotransferase, mitochondrial | *Oat* | 18242 | 10.02 | 2.28 | 1.28E+07 | 1.07E+07 | 12 | 5 |
| **Q9D0J8** | Parathymosin | *Ptms* | 69202 | 21.78 | 10.89 | 2.55E+07 | 3.81E+07 | 27 | 18 |
| **O70400** | PDZ and LIM domain protein 1 | *Pdlim1* | 54132 | 7.34 | 3.06 | 1.41E+07 | 4.97E+06 | 4 | 2 |
| **P17742** | Peptidyl-prolyl cis-trans isomerase A | *Ppia* | 268373 | 32.93 | 32.93 | 2.01E+08 | 5.48E+07 | 67 | 33 |
| **P24369** | Peptidyl-prolyl cis-trans isomerase B | *Ppib* | 19035 | 25.46 | 25.46 | 3.49E+07 | 2.61E+07 | 26 | 22 |
| **P30412** | Peptidyl-prolyl cis-trans isomerase C | *Ppic* | 19038 | 6.13 | 6.13 | 1.09E+07 | 7.75E+06 | 4 | 3 |
| **P26883** | Peptidyl-prolyl cis-trans isomerase FKBP1A | *Fkbp1a* | 14225 | 12.04 | 12.04 | 1.35E+07 | 1.02E+07 | 4 | 5 |
| **P45878** | Peptidyl-prolyl cis-trans isomerase FKBP2 | *Fkbp2* | 14227 | 8.57 | 8.57 | 1.82E+07 | 7.62E+06 | 3 | 2 |
| **P30416** | Peptidyl-prolyl cis-trans isomerase FKBP4 | *Fkbp4* | 14228 | 4.59 | 3.06 | 6.99E+06 | 1.26E+07 | 3 | 5 |
| **P35700** | Peroxiredoxin-1 | *Prdx1* | 18477 | 40.7 | 40.7 | 1.20E+08 | 6.25E+07 | 61 | 48 |
| **Q61171** | Peroxiredoxin-2 | *Prdx2* | 21672 | 23.23 | 22.73 | 2.15E+07 | 1.46E+07 | 19 | 14 |
| **P99029** | Peroxiredoxin-5, mitochondrial | *Prdx5* | 54683 | 30.48 | 22.38 | 3.66E+07 | 2.19E+07 | 22 | 14 |
| **O08709** | Peroxiredoxin-6 | *Prdx6* | 11758 | 49.11 | 39.73 | 2.19E+08 | 7.86E+07 | 100 | 54 |
| **P70296** | Phosphatidylethanolamine-binding protein 1 | *Pebp1* | 23980 | 14.44 | 14.44 | 4.69E+07 | 2.87E+07 | 11 | 11 |
| **Q9CYR6** | Phosphoacetylglucosamine mutase | *Pgm3* | 109785 | 4.98 | 1.85 | 2.44E+06 | 8.67E+05 | 2 | 1 |
| **Q9D0F9** | Phosphoglucomutase-1 | *Pgm1* | 72157 | 19.75 | 17.08 | 2.04E+07 | 1.56E+07 | 32 | 29 |
| **P09411** | Phosphoglycerate kinase 1 | *Pgk1* | 18655 | 12.47 | 14.39 | 3.57E+07 | 2.05E+07 | 19 | 20 |
| **P22777** | Plasminogen activator inhibitor 1 | *Serpine1* | 18787 | 10.2 | 14.68 | 6.02E+06 | 7.39E+06 | 9 | 12 |
| **Q61233** | Plastin-2 | *Lcp1* | 18826 | 25.36 | 25.36 | 5.27E+07 | 4.28E+07 | 68 | 82 |
| **Q99K51** | Plastin-3 | *Pls3* | 102866 | 18.25 | 11.11 | 4.22E+07 | 2.67E+07 | 36 | 24 |
| **Q08857** | Platelet glycoprotein 4 | *Cd36* | 12491 | 2.54 | 2.54 | 3.04E+06 | 1.83E+06 | 2 | 1 |
| **Q9QXS1** | Plectin | *Plec* | 18810 | 1.45 | 0.32 | 3.41E+06 | 2.06E+06 | 10 | 2 |
| **Q8CG72** | Poly(ADP-ribose) glycohydrolase ARH3 | *Adprhl2* | 100206 | 3.24 | 3.24 | 0.00E+00 | 1.02E+07 | 2 | 2 |
| **P60335** | Poly(rC)-binding protein 1 | *Pcbp1* | 23983 | 3.65 | 3.65 | 8.81E+06 | 4.90E+06 | 4 | 3 |
| **Q61990** | Poly(rC)-binding protein 2 | *Pcbp2* | 18521 | 3.59 | 3.59 | 3.66E+06 | 1.55E+06 | 1 | 1 |
| **Q3UEB3** | Poly(U)-binding-splicing factor PUF60 | *Puf60* | 67959 | 1.77 | 1.77 | 8.91E+05 | 1.21E+06 | 2 | 2 |
| **P29341** | Polyadenylate-binding protein 1 | *Pabpc1* | 18458 | 4.72 | 3.3 | 3.67E+06 | 3.97E+06 | 4 | 4 |
| **P0CG49** | Polyubiquitin-B | *Ubb* | 22187 | 32.79 | 32.79 | 4.23E+08 | 3.50E+08 | 25 | 37 |
| **Q03958** | Prefoldin subunit 6 | *Pfdn6* | 14976 | 11.02 | 11.02 | 1.94E+06 | 1.42E+06 | 2 | 1 |
| **P48678** | Prelamin-A/C | *Lmna* | 16905 | 29.02 | 19.55 | 1.97E+07 | 7.64E+06 | 58 | 34 |
| **P62962** | Profilin-1 | *Pfn1* | 18643 | 57.86 | 57.86 | 4.86E+07 | 5.08E+07 | 47 | 50 |
| **Q9WU78** | Programmed cell death 6-interacting protein | *Pdcd6ip* | 18571 | 1.5 | 1.5 | 2.70E+06 | 2.84E+06 | 2 | 1 |
| **P97371** | Proteasome activator complex subunit 1 | *Psme1* | 19186 | 26.51 | 29.32 | 3.74E+07 | 5.13E+07 | 28 | 45 |
| **P97372** | Proteasome activator complex subunit 2 | *Psme2* | 19188 | 13.81 | 16.74 | 5.17E+06 | 1.25E+07 | 7 | 15 |
| **Q9R1P4** | Proteasome subunit alpha type-1 | *Psma1* | 26440 | 4.56 | 4.56 | 1.52E+07 | 2.51E+07 | 5 | 8 |
| **O70435** | Proteasome subunit alpha type-3 | *Psma3* | 19167 | 8.63 | 8.63 | 6.64E+06 | 7.20E+06 | 7 | 7 |
| **Q9R1P0** | Proteasome subunit alpha type-4 | *Psma4* | 26441 | 6.9 | 6.9 | 1.19E+07 | 1.00E+07 | 7 | 9 |
| **Q9Z2U1** | Proteasome subunit alpha type-5 | *Psma5* | 26442 | 8.71 | 8.71 | 1.11E+07 | 1.78E+07 | 8 | 7 |
| **Q9QUM9** | Proteasome subunit alpha type-6 | *Psma6* | 26443 | 5.28 | 5.28 | 1.51E+07 | 1.93E+07 | 4 | 6 |
| **Q9Z2U0** | Proteasome subunit alpha type-7 | *Psma7* | 26444 | 19.76 | 19.76 | 1.84E+07 | 2.28E+07 | 14 | 22 |
| **O09061** | Proteasome subunit beta type-1 | *Psmb1* | 19170 | 9.58 | 5.83 | 9.36E+06 | 5.20E+06 | 5 | 1 |
| **Q9R1P3** | Proteasome subunit beta type-2 | *Psmb2* | 26445 | 4.98 | 4.98 | 2.10E+06 | 4.05E+06 | 5 | 6 |
| **Q9R1P1** | Proteasome subunit beta type-3 | *Psmb3* | 26446 | 11.22 | 3.41 | 9.06E+06 | 9.22E+06 | 7 | 2 |
| **P99026** | Proteasome subunit beta type-4 | *Psmb4* | 19172 | 12.88 | 12.88 | 7.05E+06 | 1.08E+07 | 7 | 12 |
| **Q60692** | Proteasome subunit beta type-6 | *Psmb6* | 19175 | 8.82 | 4.2 | 7.24E+06 | 3.42E+06 | 7 | 1 |
| **P70195** | Proteasome subunit beta type-7 | *Psmb7* | 19177 | 10.47 | 10.47 | 4.24E+06 | 3.24E+06 | 9 | 5 |
| **P28063** | Proteasome subunit beta type-8 | *Psmb8* | 16913 | 9.06 | 13.04 | 1.17E+07 | 1.85E+07 | 7 | 13 |
| **O35522** | Proteasome subunit beta type-9 | *Psmb9* | 16912 | 4.11 | 8.68 | 5.31E+06 | 1.02E+07 | 1 | 7 |
| **P09103** | Protein disulfide-isomerase | *P4hb* | 18453 | 15.72 | 12.38 | 3.65E+07 | 2.84E+07 | 49 | 39 |
| **P27773** | Protein disulfide-isomerase A3 | *Pdia3* | 14827 | 31.09 | 28.71 | 3.60E+07 | 3.12E+07 | 73 | 73 |
| **P08003** | Protein disulfide-isomerase A4 | *Pdia4* | 12304 | 18.5 | 14.58 | 1.15E+07 | 1.11E+07 | 27 | 33 |
| **Q922R8** | Protein disulfide-isomerase A6 | *Pdia6* | 71853 | 28.86 | 28.86 | 5.08E+07 | 3.86E+07 | 53 | 41 |
| **Q99LX0** | Protein DJ-1 | *Park7* | 57320 | 12.17 | 12.7 | 1.19E+07 | 8.41E+06 | 8 | 5 |
| **Q921M7** | Protein FAM49B | *Fam49b* | 223601 | 4.01 | 4.01 | 2.20E+06 | 1.87E+06 | 1 | 2 |
| **Q9WVE8** | Protein kinase C and casein kinase substrate in neurons protein 2 | *Pacsin2* | 23970 | 3.7 | 3.7 | 1.03E+07 | 4.86E+06 | 3 | 2 |
| **P97352** | Protein S100-A13 | *S100a13* | 20196 | 20.41 | 20.41 | 7.03E+06 | 5.54E+06 | 7 | 4 |
| **P97816** | Protein S100-G | *S100g* | 12309 | 27.85 | 27.85 | 9.18E+07 | 2.04E+07 | 20 | 11 |
| **Q9D1M0** | Protein SEC13 homolog | *Sec13* | 110379 | 5.28 | 5.28 | 5.58E+05 | 8.80E+05 | 1 | 2 |
| **Q9EQU5** | Protein SET | *Set* | 56086 | 7.27 | 7.27 | 1.09E+07 | 1.89E+07 | 7 | 10 |
| **P26350** | Prothymosin alpha | *Ptma* | 19231 | 20.72 | 20.72 | 1.65E+06 | 2.29E+06 | 6 | 8 |
| **P61458** | Pterin-4-alpha-carbinolamine dehydratase | *Pcbd1* | 13180 | 15.38 | 15.38 | 2.46E+06 | 2.08E+06 | 1 | 3 |
| **P50405** | Pulmonary surfactant-associated protein B | *Sftpb* | 20388 | 2.39 | 2.39 | 1.30E+08 | 7.46E+07 | 10 | 12 |
| **P21841** | Pulmonary surfactant-associated protein C | *Sftpc* | 20389 | 15.03 | 15.03 | 1.39E+07 | 7.26E+06 | 9 | 5 |
| **P50404** | Pulmonary surfactant-associated protein D | *Sftpd* | 20390 | 3.74 | 6.95 | 3.34E+06 | 3.84E+06 | 1 | 4 |
| **P23492** | Purine nucleoside phosphorylase | *Pnp* | 18950 | 14.88 | 3.46 | 6.22E+06 | 1.15E+07 | 8 | 3 |
| **Q11011** | Puromycin-sensitive aminopeptidase | *Npepps* | 19155 | 2.5 | 1.2 | 3.09E+06 | 2.16E+06 | 2 | 1 |
| **Q64G17** | Putative acidic leucine-rich nuclear phosphoprotein 32 family member C | *Anp32c* | 448829 | 8.94 | 8.94 | 1.49E+07 | 1.51E+07 | 3 | 5 |
| **P52480** | Pyruvate kinase PKM | *Pkm* | 18746 | 24.67 | 24.48 | 4.19E+07 | 2.91E+07 | 56 | 51 |
| **P50396** | Rab GDP dissociation inhibitor alpha | *Gdi1* | 14567 | 8.95 | 8.95 | 1.09E+07 | 6.02E+06 | 12 | 9 |
| **Q61598** | Rab GDP dissociation inhibitor beta | *Gdi2* | 14569 | 7.19 | 9.89 | 1.07E+07 | 8.09E+06 | 10 | 9 |
| **P26043** | Radixin | *Rdx* | 19684 | 13.72 | 2.74 | 2.36E+07 | 1.86E+07 | 23 | 9 |
| **P34022** | Ran-specific GTPase-activating protein | *Ranbp1* | 19385 | 5.42 | 5.42 | 1.03E+07 | 8.74E+06 | 2 | 3 |
| **P97855** | Ras GTPase-activating protein-binding protein 1 | *G3bp1* | 27041 | 3.66 | 3.66 | 5.10E+06 | 4.67E+06 | 2 | 3 |
| **Q9JKF1** | Ras GTPase-activating-like protein IQGAP1 | *Iqgap1* | 29875 | 15.87 | 9.41 | 1.65E+07 | 7.14E+06 | 49 | 20 |
| **Q9DCV4** | Regulator of microtubule dynamics protein 1 | *Rmdn1* | 66302 | 4.26 | 4.26 | 2.24E+06 | 1.37E+06 | 2 | 1 |
| **P24549** | Retinal dehydrogenase 1 | *Aldh1a1* | 11668 | 14.37 | 14.97 | 1.99E+07 | 1.16E+07 | 24 | 16 |
| **Q99PT1** | Rho GDP-dissociation inhibitor 1 | *Arhgdia* | 192662 | 25.49 | 25.49 | 5.86E+07 | 4.12E+07 | 38 | 27 |
| **Q91VI7** | Ribonuclease inhibitor | *Rnh1* | 107702 | 8.77 | 8.77 | 5.52E+06 | 3.93E+06 | 6 | 5 |
| **Q99PL5** | Ribosome-binding protein 1 | *Rrbp1* | 81910 | 4.49 | 3.12 | 3.50E+06 | 4.63E+06 | 9 | 11 |
| **Q9JI75** | Ribosyldihydronicotinamide dehydrogenase [quinone] | *Nqo2* | 18105 | 4.76 | 4.76 | 5.40E+06 | 3.23E+06 | 2 | 1 |
| **Q91VM5** | RNA binding motif protein, X-linked-like-1 | *Rbmxl1* | 19656 | 9.02 | 5.41 | 7.94E+06 | 5.15E+06 | 8 | 6 |
| **Q61545** | RNA-binding protein EWS | *Ewsr1* | 14030 | 3.51 | 2.14 | 4.47E+06 | 4.43E+06 | 4 | 3 |
| **P56959** | RNA-binding protein FUS | *Fus* | 233908 | 4.44 | 2.7 | 6.62E+06 | 7.08E+06 | 6 | 5 |
| **P17563** | Selenium-binding protein 1 | *Selenbp1* | 20341 | 25.42 | 18.22 | 1.76E+08 | 9.77E+07 | 84 | 64 |
| **P58389** | Serine/threonine-protein phosphatase 2A activator | *Ppp2r4* | 110854 | 3.1 | 3.1 | 3.07E+06 | 2.01E+06 | 2 | 1 |
| **Q921I1** | Serotransferrin | *Tf* | 22041 | 1.15 | 1.15 | 2.73E+06 | 2.48E+06 | 1 | 1 |
| **Q60854** | Serpin B6 | *Serpinb6* | 20719 | 12.17 | 3.44 | 5.45E+06 | 3.19E+06 | 15 | 2 |
| **Q9JJU8** | SH3 domain-binding glutamic acid-rich-like protein | *Sh3bgrl* | 56726 | 20.18 | 11.4 | 5.01E+06 | 6.37E+06 | 7 | 5 |
| **Q91VW3** | SH3 domain-binding glutamic acid-rich-like protein 3 | *Sh3bgrl3* | 73723 | 16.13 | 26.88 | 5.76E+06 | 2.94E+06 | 2 | 4 |
| **P62320** | Small nuclear ribonucleoprotein Sm D3 | *Snrpd3* | 67332 | 15.08 | 7.94 | 4.42E+06 | 3.63E+06 | 4 | 1 |
| **P61957** | Small ubiquitin-related modifier 2 | *Sumo2* | 170930 | 12.63 | 12.63 | 1.65E+07 | 1.97E+07 | 5 | 6 |
| **Q9DBP0** | Sodium-dependent phosphate transport protein 2B | *Slc34a2* | 20531 | 2.15 | 2.15 | 1.30E+07 | 9.65E+06 | 4 | 4 |
| **Q64442** | Sorbitol dehydrogenase | *Sord* | 20322 | 14.29 | 11.76 | 1.55E+07 | 4.17E+06 | 14 | 6 |
| **P16546** | Spectrin alpha chain, non-erythrocytic 1 | *Sptan1* | 20740 | 9.02 | 4.73 | 1.04E+07 | 5.48E+06 | 43 | 18 |
| **Q62261** | Spectrin beta chain, non-erythrocytic 1 | *Sptbn1* | 20742 | 9.69 | 4.15 | 8.82E+06 | 4.33E+06 | 31 | 10 |
| **Q9WTX5** | S-phase kinase-associated protein 1 | *Skp1* | 21402 | 15.34 | 7.36 | 6.49E+06 | 5.01E+06 | 3 | 4 |
| **Q9Z1N5** | Spliceosome RNA helicase Ddx39b | *Ddx39b* | 53817 | 1.87 | 3.27 | 9.94E+05 | 4.59E+06 | 2 | 2 |
| **Q8VIJ6** | Splicing factor, proline- and glutamine-rich | *Sfpq* | 71514 | 2.29 | 2.29 | 8.13E+06 | 1.99E+06 | 6 | 2 |
| **Q60864** | Stress-induced-phosphoprotein 1 | *Stip1* | 20867 | 2.39 | 4.05 | 1.09E+07 | 6.25E+06 | 3 | 5 |
| **P28862** | Stromelysin-1 | *Mmp3* | 17392 | 10.9 | 2.1 | 1.38E+07 | 4.06E+06 | 14 | 2 |
| **Q9D0K2** | Succinyl-CoA:3-ketoacid coenzyme A transferase 1, mitochondrial | *Oxct1* | 67041 | 4.42 | 2.31 | 4.70E+06 | 9.74E+05 | 3 | 1 |
| **P08228** | Superoxide dismutase [Cu-Zn] | *Sod1* | 20655 | 7.14 | 7.14 | 4.07E+07 | 4.06E+07 | 7 | 9 |
| **P09671** | Superoxide dismutase [Mn], mitochondrial | *Sod2* | 20656 | 9.91 | 9.91 | 5.39E+07 | 3.16E+07 | 12 | 14 |
| **O35988** | Syndecan-4 | *Sdc4* | 20971 | 7.07 | 7.07 | 6.81E+06 | 4.63E+06 | 2 | 2 |
| **P26039** | Talin-1 | *Tln1* | 21894 | 2.4 | 0.47 | 3.00E+06 | 1.33E+06 | 5 | 1 |
| **P10639** | Thioredoxin | *Txn* | 22166 | 29.52 | 22.86 | 8.29E+07 | 6.81E+07 | 17 | 18 |
| **Q91W90** | Thioredoxin domain-containing protein 5 | *Txndc5* | 105245 | 5.52 | 3.36 | 4.07E+06 | 2.87E+06 | 3 | 3 |
| **Q9JMH6** | Thioredoxin reductase 1, cytoplasmic | *Txnrd1* | 50493 | 6.2 | 5.38 | 7.48E+06 | 5.82E+06 | 9 | 6 |
| **P20108** | Thioredoxin-dependent peroxide reductase, mitochondrial | *Prdx3* | 11757 | 11.67 | 13.23 | 8.57E+06 | 3.41E+06 | 7 | 4 |
| **P52196** | Thiosulfate sulfurtransferase | *Tst* | 22117 | 8.08 | 4.04 | 1.00E+07 | 4.54E+06 | 5 | 2 |
| **P35441** | Thrombospondin-1 | *Thbs1* | 21825 | 1.97 | 0.94 | 3.85E+06 | 5.34E+06 | 3 | 2 |
| **Q93092** | Transaldolase | *Taldo1* | 21351 | 14.24 | 8.01 | 5.70E+06 | 1.95E+06 | 15 | 4 |
| **Q9WVA4** | Transgelin-2 | *Tagln2* | 21346 | 31.16 | 18.59 | 6.70E+07 | 4.54E+07 | 45 | 26 |
| **Q01853** | Transitional endoplasmic reticulum ATPase | *Vcp* | 269523 | 22.21 | 19.23 | 2.91E+07 | 2.18E+07 | 71 | 48 |
| **P40142** | Transketolase | *Tkt* | 21881 | 19.26 | 14.61 | 1.11E+08 | 5.01E+07 | 70 | 55 |
| **Q62348** | Translin | *Tsn* | 22099 | 10.09 | 10.09 | 6.94E+06 | 4.45E+06 | 6 | 2 |
| **Q9JHJ0** | Tropomodulin-3 | *Tmod3* | 50875 | 8.24 | 8.24 | 5.57E+06 | 3.16E+06 | 3 | 3 |
| **P58771** | Tropomyosin alpha-1 chain | *Tpm1* | 22003 | 16.9 | 14.79 | 7.48E+07 | 4.65E+07 | 43 | 39 |
| **P21107** | Tropomyosin alpha-3 chain | *Tpm3* | 59069 | 18.25 | 11.58 | 7.89E+07 | 5.31E+07 | 25 | 25 |
| **Q6IRU2** | Tropomyosin alpha-4 chain | *Tpm4* | 326618 | 22.58 | 20.16 | 2.90E+07 | 1.48E+07 | 24 | 23 |
| **P58774** | Tropomyosin beta chain | *Tpm2* | 22004 | 10.21 | 11.97 | 5.42E+07 | 3.41E+07 | 13 | 16 |
| **P05213** | Tubulin alpha-1B chain | *Tuba1b* | 22143 | 18.4 | 20.62 | 1.37E+07 | 1.06E+07 | 16 | 13 |
| **P68373** | Tubulin alpha-1C chain | *Tuba1c* | 22146 | 13.81 | 20.27 | 1.37E+07 | 1.05E+07 | 15 | 14 |
| **P68372** | Tubulin beta-4B chain | *Tubb4b* | 227613 | 8.76 | 8.76 | 5.31E+06 | 6.09E+06 | 5 | 7 |
| **Q9CRB6** | Tubulin polymerization-promoting protein family member 3 | *Tppp3* | 67971 | 24.43 | 15.34 | 1.89E+07 | 1.16E+07 | 13 | 7 |
| **P48428** | Tubulin-specific chaperone A | *Tbca* | 21371 | 31.48 | 14.81 | 3.36E+06 | 1.65E+06 | 7 | 2 |
| **Q62393** | Tumor protein D52 | *Tpd52* | 21985 | 11.16 | 11.16 | 9.07E+06 | 5.38E+06 | 6 | 6 |
| **Q9CYZ2** | Tumor protein D54 | *Tpd52l2* | 66314 | 11.36 | 4.09 | 6.35E+06 | 3.36E+06 | 5 | 1 |
| **P61087** | Ubiquitin-conjugating enzyme E2 K | *Ube2k* | 53323 | 6 | 13 | 5.26E+06 | 2.43E+06 | 1 | 3 |
| **P68037** | Ubiquitin-conjugating enzyme E2 L3 | *Ube2l3* | 22195 | 5.84 | 5.84 | 1.27E+06 | 1.58E+06 | 1 | 1 |
| **P61089** | Ubiquitin-conjugating enzyme E2 N | *Ube2n* | 93765 | 13.82 | 13.82 | 1.16E+07 | 9.47E+06 | 6 | 5 |
| **Q9D2M8** | Ubiquitin-conjugating enzyme E2 variant 2 | *Ube2v2* | 70620 | 11.72 | 6.9 | 6.60E+06 | 7.05E+06 | 2 | 3 |
| **Q9DBP5** | UMP-CMP kinase | *Cmpk1* | 66588 | 5.61 | 5.61 | 7.26E+06 | 1.50E+07 | 2 | 1 |
| **Q06318** | Uteroglobin | *Scgb1a1* | 22287 | 10.42 | 10.42 | 1.59E+08 | 1.14E+08 | 11 | 16 |
| **P54728** | UV excision repair protein RAD23 homolog B | *Rad23b* | 19359 | 2.4 | 4.57 | 7.30E+06 | 3.96E+06 | 2 | 2 |
| **Q9QY76** | Vesicle-associated membrane protein-associated protein B | *Vapb* | 56491 | 4.94 | 4.94 | 1.69E+07 | 5.99E+06 | 5 | 4 |
| **P20152** | Vimentin | *Vim* | 22352 | 19.1 | 22.75 | 1.30E+07 | 2.05E+07 | 29 | 44 |
| **Q64727** | Vinculin | *Vcl* | 22330 | 20.36 | 14.63 | 2.05E+07 | 1.02E+07 | 59 | 32 |
| **P50516** | V-type proton ATPase catalytic subunit A | *Atp6v1a* | 11964 | 5.35 | 1.46 | 1.76E+06 | 4.11E+05 | 7 | 1 |
| **P62814** | V-type proton ATPase subunit B, brain isoform | *Atp6v1b2* | 11966 | 7.05 | 4.89 | 5.33E+06 | 4.34E+06 | 8 | 4 |
| **P50518** | V-type proton ATPase subunit E 1 | *Atp6v1e1* | 11973 | 9.29 | 9.29 | 4.26E+06 | 2.74E+06 | 4 | 4 |
| **Q9CR51** | V-type proton ATPase subunit G 1 | *Atp6v1g1* | 66290 | 9.32 | 9.32 | 4.68E+06 | 3.59E+06 | 3 | 3 |
| **Q00519** | Xanthine dehydrogenase/oxidase | *Xdh* | 22436 | 6.37 | 5.32 | 9.04E+06 | 6.40E+06 | 12 | 11 |

**Table S6: List of proteins (Ʃ 129) only identified in BALF from SPC-HA mice. Coverage: percentage of protein covered by matched peptide spectra; area: mean area of the 3 peptides (matched with the respective protein) identified with the highest area of the corresponding peaks in the extracted ion chromatograms; # PSM: number of peptide-spectrum matches; count of peptide spectra (identified by MS/MS) that matched for the respective protein**

| **Accession ID** | **Protein** | **Gene symbol** | **Entrez Gene ID** | **Coverage  SPC-HA** | **Area  SPC-HA** | **# PSM  SPC-HA** |
| --- | --- | --- | --- | --- | --- | --- |
| **Q3UHX2** | 28 kDa heat- and acid-stable phosphoprotein | *Pdap1* | 231887 | 7.18 | 4.02E+06 | 2 |
| **Q9Z0S1** | 3'(2'),5'-bisphosphate nucleotidase 1 | *Bpnt1* | 23827 | 2.6 | 3.96E+06 | 1 |
| **Q8QZS1** | 3-hydroxyisobutyryl-CoA hydrolase, mitochondrial | *Hibch* | 227095 | 5.71 | 3.86E+06 | 5 |
| **Q8K010** | 5-oxoprolinase | *Oplah* | 75475 | 2.02 | 2.46E+06 | 2 |
| **Q9CQ60** | 6-phosphogluconolactonase | *Pgls* | 66171 | 10.12 | 4.89E+06 | 2 |
| **P33434** | 72 kDa type IV collagenase | *Mmp2* | 17390 | 2.87 | 3.16E+06 | 2 |
| **Q91XA9** | Acidic mammalian chitinase | *Chia* | 81600 | 3.81 | 4.63E+06 | 3 |
| **Q9Z2N8** | Actin-like protein 6A | *Actl6a* | 56456 | 3.03 | 1.08E+06 | 2 |
| **Q6P5E6** | ADP-ribosylation factor-binding protein GGA2 | *Gga2* | 74105 | 1.16 | 2.08E+06 | 1 |
| **P97449** | Aminopeptidase N | *Anpep* | 16790 | 1.24 | 1.64E+06 | 1 |
| **G5E8K5** | Ankyrin-3 | *Ank3* | 11735 | 0.66 | 5.63E+06 | 1 |
| **O35639** | Annexin A3 | *Anxa3* | 11745 | 4.64 | 4.15E+06 | 1 |
| **O35841** | Apoptosis inhibitor 5 | *Api5* | 11800 | 4.37 | 2.51E+06 | 2 |
| **Q7SIG6** | Arf-GAP with SH3 domain, ANK repeat and PH domain-containing protein 2 | *Asap2* | 211914 | 0.84 | 2.89E+07 | 1 |
| **Q91YI0** | Argininosuccinate lyase | *Asl* | 109900 | 4.31 | 3.31E+06 | 2 |
| **Q9DC29** | ATP-binding cassette sub-family B member 6, mitochondrial | *Abcb6* | 74104 | 0.95 | 1.34E+06 | 1 |
| **Q9JLV1** | BAG family molecular chaperone regulator 3 | *Bag3* | 29810 | 2.25 | 2.63E+06 | 1 |
| **Q99NF1** | Beta,beta-carotene 9',10'-oxygenase | *Bco2* | 170752 | 2.82 | 4.48E+06 | 1 |
| **P28653** | Biglycan | *Bgn* | 12111 | 2.98 | 1.59E+06 | 2 |
| **Q8R016** | Bleomycin hydrolase | *Blmh* | 104184 | 2.64 | 9.83E+06 | 2 |
| **O35855** | Branched-chain-amino-acid aminotransferase, mitochondrial | *Bcat2* | 12036 | 2.04 | 2.17E+06 | 1 |
| **Q9DB16** | Calcium-binding protein 39-like | *Cab39l* | 69008 | 6.23 | 1.52E+06 | 2 |
| **Q9D7J7** | Calpain small subunit 2 | *Capns2* | 69543 | 2.83 | 2.51E+06 | 1 |
| **Q08093** | Calponin-2 | *Cnn2* | 12798 | 3.61 | 3.38E+06 | 2 |
| **P14211** | Calreticulin | *Calr* | 12317 | 6.73 | 4.45E+06 | 4 |
| **P00920** | Carbonic anhydrase 2 | *Ca2* | 12349 | 7.31 | 4.25E+06 | 5 |
| **Q9D7S9** | Charged multivesicular body protein 5 | *Chmp5* | 76959 | 7.31 | 2.42E+06 | 1 |
| **P83917** | Chromobox protein homolog 1 | *Cbx1* | 12412 | 14.59 | 3.17E+06 | 3 |
| **P59242** | Cingulin | *Cgn* | 70737 | 0.84 | 1.06E+06 | 1 |
| **Q68FD5** | Clathrin heavy chain 1 | *Cltc* | 67300 | 1.01 | 4.23E+06 | 2 |
| **O08585** | Clathrin light chain A | *Clta* | 12757 | 3.83 | 6.98E+05 | 1 |
| **P61924** | Coatomer subunit zeta-1 | *Copz1* | 56447 | 6.21 | 3.00E+06 | 2 |
| **P11087** | Collagen alpha-1(I) chain | *Col1a1* | 12842 | 1.24 | 8.83E+05 | 1 |
| **Q62426** | Cystatin-B | *Cstb* | 13014 | 11.22 | 1.07E+07 | 2 |
| **P99028** | Cytochrome b-c1 complex subunit 6, mitochondrial | *Uqcrh* | 66576 | 16.85 | 1.13E+07 | 5 |
| **Q8R0Y6** | Cytosolic 10-formyltetrahydrofolate dehydrogenase | *Aldh1l1* | 107747 | 2.66 | 3.50E+06 | 2 |
| **Q9CQJ6** | Density-regulated protein | *Denr* | 68184 | 8.08 | 2.34E+06 | 2 |
| **P62627** | Dynein light chain roadblock-type 1 | *Dynlrb1* | 67068 | 12.5 | 5.29E+06 | 1 |
| **Q7TNG5** | Echinoderm microtubule-associated protein-like 2 | *Eml2* | 72205 | 3.7 | 6.35E+06 | 3 |
| **Q9DCW4** | Electron transfer flavoprotein subunit beta | *Etfb* | 110826 | 11.37 | 1.88E+06 | 3 |
| **Q99K30** | Epidermal growth factor receptor kinase substrate 8-like protein 2 | *Eps8l2* | 98845 | 3.7 | 2.32E+06 | 3 |
| **Q9WUK2** | Eukaryotic translation initiation factor 4H | *Eif4h* | 22384 | 5.24 | 7.40E+06 | 2 |
| **P63242** | Eukaryotic translation initiation factor 5A-1 | *Eif5a* | 276770 | 5.19 | 2.09E+06 | 1 |
| **P97447** | Four and a half LIM domains protein 1 | *Fhl1* | 14199 | 4.29 | 1.46E+06 | 1 |
| **Q921G8** | Gamma-tubulin complex component 2 | *Tubgcp2* | 74237 | 0.77 | 1.19E+07 | 1 |
| **P16406** | Glutamyl aminopeptidase | *Enpep* | 13809 | 7.2 | 4.08E+06 | 9 |
| **P10648** | Glutathione S-transferase A2 | *Gsta2* | 14858 | 4.5 | 9.78E+06 | 1 |
| **P30115** | Glutathione S-transferase A3 | *Gsta3* | 14859 | 4.07 | 4.76E+06 | 1 |
| **P43081** | Guanylyl cyclase-activating protein 1 | *Guca1a* | 14913 | 3.96 | 2.97E+06 | 1 |
| **O88569** | Heterogeneous nuclear ribonucleoproteins A2/B1 | *Hnrnpa2b1* | 53379 | 7.93 | 4.30E+07 | 9 |
| **Q9Z204** | Heterogeneous nuclear ribonucleoproteins C1/C2 | *Hnrnpc* | 15381 | 3.19 | 8.17E+06 | 3 |
| **P63158** | High mobility group protein B1 | *Hmgb1* | 15289 | 6.05 | 4.62E+06 | 4 |
| **P17095** | High mobility group protein HMG-I/HMG-Y | *Hmga1* | 15361 | 14.02 | 5.32E+05 | 2 |
| **Q60972** | Histone-binding protein RBBP4 | *Rbbp4* | 19646 | 5.65 | 5.70E+06 | 3 |
| **Q61425** | Hydroxyacyl-coenzyme A dehydrogenase, mitochondrial | *Hadh* | 15107 | 5.41 | 1.05E+07 | 5 |
| **P40936** | Indolethylamine N-methyltransferase | *Inmt* | 21743 | 13.26 | 9.18E+06 | 8 |
| **O88792** | Junctional adhesion molecule A | *F11r* | 16456 | 6.33 | 4.45E+06 | 1 |
| **Q3TTY5** | Keratin, type II cytoskeletal 2 epidermal | *Krt2* | 16681 | 4.24 | 8.51E+06 | 10 |
| **Q99KP3** | Lambda-crystallin homolog | *Cryl1* | 68631 | 5.33 | 1.63E+06 | 1 |
| **P02468** | Laminin subunit gamma-1 | *Lamc1* | 226519 | 0.87 | 1.81E+06 | 1 |
| **P24527** | Leukotriene A-4 hydrolase | *Lta4h* | 16993 | 2.13 | 4.10E+06 | 2 |
| **Q9D358** | Low molecular weight phosphotyrosine protein phosphatase | *Acp1* | 11431 | 6.96 | 4.60E+06 | 1 |
| **P17047** | Lysosome-associated membrane glycoprotein 2 | *Lamp2* | 16784 | 1.93 | 1.05E+07 | 2 |
| **Q9EQK5** | Major vault protein | *Mvp* | 78388 | 1.97 | 3.52E+06 | 2 |
| **P25785** | Metalloproteinase inhibitor 2 | *Timp2* | 21858 | 6.36 | 3.39E+06 | 1 |
| **Q9Z2D6** | Methyl-CpG-binding protein 2 | *Mecp2* | 17257 | 10.12 | 2.93E+06 | 4 |
| **Q9EQ20** | Methylmalonate-semialdehyde dehydrogenase [acylating], mitochondrial | *Aldh6a1* | 104776 | 2.06 | 1.89E+06 | 1 |
| **Q9CRB9** | MICOS complex subunit Mic19 | *Chchd3* | 66075 | 4.85 | 2.24E+06 | 1 |
| **Q0P557** | Mitochondria-eating protein | *Spata18* | 73472 | 1.86 | 1.19E+06 | 1 |
| **P62075** | Mitochondrial import inner membrane translocase subunit Tim13 | *Timm13* | 30055 | 14.74 | 3.28E+06 | 1 |
| **Q6PEB6** | MOB-like protein phocein | *Mob4* | 19070 | 9.78 | 1.50E+06 | 1 |
| **Q69ZN7** | Myoferlin | *Myof* | 226101 | 0.54 | 3.15E+06 | 1 |
| **P82343** | N-acylglucosamine 2-epimerase | *Renbp* | 19703 | 3.26 | 2.64E+06 | 1 |
| **Q8BHN3** | Neutral alpha-glucosidase AB | *Ganab* | 14376 | 1.38 | 8.04E+06 | 3 |
| **Q8R1F1** | Niban-like protein 1 | *Fam129b* | 227737 | 2.8 | 5.03E+06 | 3 |
| **P18608** | Non-histone chromosomal protein HMG-14 | *Hmgn1* | 15312 | 31.25 | 2.54E+06 | 1 |
| **P32020** | Non-specific lipid-transfer protein | *Scp2* | 20280 | 3.66 | 3.72E+06 | 3 |
| **Q80XU3** | Nuclear ubiquitous casein and cyclin-dependent kinase substrate 1 | *Nucks1* | 98415 | 8.12 | 6.42E+06 | 3 |
| **Q9WV85** | Nucleoside diphosphate kinase 3 | *Nme3* | 79059 | 10.06 | 4.95E+06 | 3 |
| **Q62446** | Peptidyl-prolyl cis-trans isomerase FKBP3 | *Fkbp3* | 30795 | 4.91 | 4.82E+05 | 1 |
| **Q9CWW6** | Peptidyl-prolyl cis-trans isomerase NIMA-interacting 4 | *Pin4* | 69713 | 9.16 | 7.79E+05 | 2 |
| **Q9R269** | Periplakin | *Ppl* | 19041 | 2.51 | 3.25E+06 | 6 |
| **Q9DBJ1** | Phosphoglycerate mutase 1 | *Pgam1* | 18648 | 5.51 | 5.08E+06 | 1 |
| **Q9CY58** | Plasminogen activator inhibitor 1 RNA-binding protein | *Serbp1* | 66870 | 3.93 | 5.94E+06 | 3 |
| **O54724** | Polymerase I and transcript release factor | *Ptrf* | 19285 | 10.46 | 5.10E+06 | 4 |
| **P17225** | Polypyrimidine tract-binding protein 1 | *Ptbp1* | 19205 | 3.61 | 4.69E+06 | 3 |
| **Q6NSR8** | Probable aminopeptidase NPEPL1 | *Npepl1* | 228961 | 4.58 | 2.07E+06 | 2 |
| **P50580** | Proliferation-associated protein 2G4 | *Pa2g4* | 18813 | 2.79 | 6.60E+05 | 1 |
| **Q9QUR6** | Prolyl endopeptidase | *Prep* | 19072 | 2.39 | 5.02E+06 | 2 |
| **Q99MN9** | Propionyl-CoA carboxylase beta chain, mitochondrial | *Pccb* | 66904 | 2.96 | 9.87E+05 | 2 |
| **P49722** | Proteasome subunit alpha type-2 | *Psma2* | 19166 | 4.7 | 6.95E+05 | 1 |
| **O55234** | Proteasome subunit beta type-5 | *Psmb5* | 19173 | 3.41 | 1.93E+06 | 1 |
| **Q9CQ89** | Protein CutA | *Cuta* | 67675 | 7.91 | 6.95E+06 | 1 |
| **Q99LT0** | Protein dpy-30 homolog | *Dpy30* | 66310 | 9.09 | 3.30E+06 | 1 |
| **Q62433** | Protein NDRG1 | *Ndrg1* | 17988 | 3.55 | 1.07E+06 | 2 |
| **Q9CQE1** | Protein NipSnap homolog 3B | *Nipsnap3b* | 66536 | 3.64 | 5.81E+06 | 2 |
| **P14069** | Protein S100-A6 | *S100a6* | 20200 | 8.99 | 1.71E+06 | 1 |
| **Q91V41** | Ras-related protein Rab-14 | *Rab14* | 68365 | 6.51 | 3.17E+06 | 1 |
| **P62821** | Ras-related protein Rab-1A | *Rab1A* | 19324 | 7.8 | 5.64E+06 | 1 |
| **Q8BHL4** | Retinoic acid-induced protein 3 | *Gprc5a* | 232431 | 7.58 | 6.10E+06 | 4 |
| **Q9CWY8** | Ribonuclease H2 subunit A | *Rnaseh2a* | 69724 | 2.66 | 3.10E+07 | 1 |
| **P52760** | Ribonuclease UK114 | *Hrsp12* | 15473 | 14.81 | 4.18E+06 | 4 |
| **Q9CWZ3** | RNA-binding protein 8A | *Rbm8a* | 60365 | 6.32 | 3.69E+06 | 1 |
| **Q8R4Y8** | Rotatin | *Rttn* | 246102 | 0.45 | 3.75E+07 | 1 |
| **Q99J08** | SEC14-like protein 2 | *Sec14l2* | 67815 | 2.23 | 2.23E+06 | 1 |
| **Q8R0F9** | SEC14-like protein 4 | *Sec14l4* | 103655 | 2.98 | 3.41E+06 | 1 |
| **O55131** | Septin-7 | *Sep 07* | 235072 | 2.29 | 5.97E+05 | 1 |
| **Q76MZ3** | Serine/threonine-protein phosphatase 2A 65 kDa regulatory subunit A alpha isoform | *Ppp2r1a* | 51792 | 2.21 | 9.65E+05 | 1 |
| **P07724** | Serum albumin | *Alb* | 11657 | 1.64 | 5.64E+06 | 1 |
| **P55012** | Solute carrier family 12 member 2 | *Slc12a2* | 20496 | 1 | 1.28E+06 | 1 |
| **Q9CWK8** | Sorting nexin-2 | *Snx2* | 67804 | 2.12 | 2.35E+06 | 1 |
| **P07214** | SPARC | *Sparc* | 20692 | 2.98 | 4.88E+06 | 2 |
| **Q8K4Z5** | Splicing factor 3A subunit 1 | *Sf3a1* | 67465 | 1.64 | 4.29E+06 | 2 |
| **Q921M3** | Splicing factor 3B subunit 3 | *Sf3b3* | 101943 | 1.15 | 4.41E+06 | 1 |
| **Q9DBG9** | Tax1-binding protein 3 | *Tax1bp3* | 76281 | 13.71 | 6.25E+06 | 2 |
| **P80317** | T-complex protein 1 subunit zeta | *Cct6a* | 12466 | 1.88 | 1.45E+06 | 1 |
| **Q9JLT4** | Thioredoxin reductase 2, mitochondrial | *Txnrd2* | 26462 | 2.29 | 3.34E+06 | 1 |
| **P97493** | Thioredoxin, mitochondrial | *Txn2* | 56551 | 6.02 | 4.76E+06 | 1 |
| **Q8CDN6** | Thioredoxin-like protein 1 | *Txnl1* | 53382 | 5.19 | 2.87E+06 | 1 |
| **P42669** | Transcriptional activator protein Pur-alpha | *Pura* | 19290 | 2.8 | 3.66E+06 | 1 |
| **Q9R1Q8** | Transgelin-3 | *Tagln3* | 56370 | 7.04 | 2.78E+06 | 2 |
| **P17751** | Triosephosphate isomerase | *Tpi1* | 21991 | 5.02 | 1.63E+07 | 4 |
| **O35900** | U6 snRNA-associated Sm-like protein LSm2 | *Lsm2* | 27756 | 20 | 2.45E+06 | 1 |
| **Q91ZJ5** | UTP--glucose-1-phosphate uridylyltransferase | *Ugp2* | 216558 | 4.13 | 4.59E+06 | 4 |
| **P40336** | Vacuolar protein sorting-associated protein 26A | *Vps26a* | 30930 | 5.5 | 6.84E+06 | 2 |
| **Q99KC8** | von Willebrand factor A domain-containing protein 5A | *Vwa5a* | 67776 | 1.26 | 1.13E+06 | 1 |
| **Q9D1K2** | V-type proton ATPase subunit F | *Atp6v1f* | 66144 | 10.08 | 5.78E+06 | 3 |
| **O88342** | WD repeat-containing protein 1 | *Wdr1* | 22388 | 1.65 | 5.79E+06 | 1 |
| **Q11136** | Xaa-Pro dipeptidase | *Pepd* | 18624 | 3.85 | 1.44E+06 | 1 |

**Table S7: List of proteins (Ʃ101) only identified in BALF from SPC-HAxTCR-HA mice. Coverage: percentage of protein covered by matched peptide spectra; area: mean area of the 3 peptides (matched with the respective protein) identified with the highest area of the corresponding peaks in the extracted ion chromatograms; # PSM: number of peptide-spectrum matches; count of peptide spectra (identified by MS/MS) that matched for the respective protein**

| **Accession ID** | **Protein** | **Gene symbol** | **Entrez Gene ID** | **Coverage  SPC-HAx**  **TCR-HA** | **Area  SPC-HAx**  **TCR-HA** | **# PSM  SPC-HAx**  **TCR-HA** |
| --- | --- | --- | --- | --- | --- | --- |
| **P61161** | Actin-related protein 2 | *Actr2* | 66713 | 3.05 | 0.00E+00 | 4 |
| **P56376** | Acylphosphatase-1 | *Acyp1* | 66204 | 14.14 | 3.64E+06 | 1 |
| **P28474** | Alcohol dehydrogenase class-3 | *Adh5* | 11532 | 2.14 | 3.28E+06 | 1 |
| **P55264** | Adenosine kinase | *Adk* | 11534 | 2.22 | 8.93E+06 | 1 |
| **P47739** | Aldehyde dehydrogenase, dimeric NADP-preferring | *Aldh3a1* | 11670 | 1.99 | 1.10E+06 | 1 |
| **P10107** | Annexin A1 | *Anxa1* | 16952 | 3.76 | 6.45E+05 | 1 |
| **P22892** | AP-1 complex subunit gamma-1 | *Ap1g1* | 11765 | 1.22 | 9.23E+05 | 1 |
| **Q61599** | Rho GDP-dissociation inhibitor 2 | *Arhgdib* | 11857 | 18.5 | 1.21E+07 | 15 |
| **Q9CVB6** | Actin-related protein 2/3 complex subunit 2 | *Arpc2* | 76709 | 3 | 5.81E+06 | 2 |
| **P01887** | Beta-2-microglobulin | *B2m* | 12010 | 8.4 | 1.06E+07 | 21 |
| **P51437** | Cathelin-related antimicrobial peptide | *Camp* | 12796 | 10.4 | 1.44E+07 | 5 |
| **P35564** | Calnexin | *Canx* | 12330 | 1.52 | 4.50E+06 | 4 |
| **O35350** | Calpain-1 catalytic subunit | *Capn1* | 12333 | 1.68 | 3.08E+06 | 1 |
| **P51125** | Calpastatin | *Cast* | 12380 | 2.28 | 9.12E+05 | 1 |
| **P80314** | T-complex protein 1 subunit beta | *Cct2* | 12461 | 2.24 | 1.83E+06 | 1 |
| **P80318** | T-complex protein 1 subunit gamma | *Cct3* | 12462 | 2.02 | 1.22E+06 | 1 |
| **P80315** | T-complex protein 1 subunit delta | *Cct4* | 12464 | 2.41 | 2.36E+06 | 1 |
| **P42932** | T-complex protein 1 subunit theta | *Cct8* | 12469 | 2.19 | 1.36E+06 | 1 |
| **P10810** | Monocyte differentiation antigen CD14 | *Cd14* | 12475 | 4.1 | 3.96E+06 | 1 |
| **O35744** | Chitinase-like protein 3 | *Chil3* | 12655 | 20.85 | 1.99E+07 | 22 |
| **Q04447** | Creatine kinase B-type | *Ckb* | 12709 | 5.51 | 3.18E+06 | 2 |
| **Q99KN9** | Clathrin interactor 1 | *Clint1* | 216705 | 1.74 | 2.17E+06 | 2 |
| **Q9QXT0** | Protein canopy homolog 2 | *Cnpy2* | 56530 | 5.49 | 1.14E+06 | 1 |
| **O89053** | Coronin-1A | *Coro1a* | 12721 | 3.47 | 2.86E+06 | 1 |
| **Q9CQI6** | Coactosin-like protein | *Cotl1* | 72042 | 10.56 | 6.93E+06 | 6 |
| **Q02248** | Catenin beta-1 | *Ctnnb1* | 12387 | 1.54 | 1.25E+06 | 1 |
| **O70370** | Cathepsin S | *Ctss* | 13040 | 3.82 | 6.39E+06 | 2 |
| **P48024** | Eukaryotic translation initiation factor 1 | *Eif1* | 20918 | 12.39 | 4.19E+06 | 2 |
| **Q60872** | Eukaryotic translation initiation factor 1A | *Eif1a* | 13664 | 20.83 | 3.38E+06 | 1 |
| **Q3UGC7** | Eukaryotic translation initiation factor 3 subunit J-A | *Eif3j1* | 78655 | 4.21 | 2.62E+06 | 2 |
| **P60843** | Eukaryotic initiation factor 4A-I | *Eif4a1* | 13681 | 2.46 | 2.26E+06 | 1 |
| **O55135** | Eukaryotic translation initiation factor 6 | *Eif6* | 16418 | 4.49 | 2.88E+06 | 1 |
| **Q9CQ92** | Mitochondrial fission 1 protein | *Fis1* | 66437 | 8.55 | 1.68E+06 | 1 |
| **P16858** | Glyceraldehyde-3-phosphate dehydrogenase | *Gapdh* | 14433 | 4.5 | 9.14E+06 | 4 |
| **Q01514** | Interferon-induced guanylate-binding protein 1 | *Gbp1* | 14468 | 6.62 | 6.38E+06 | 7 |
| **Q9Z0E6** | Interferon-induced guanylate-binding protein 2 | *Gbp2* | 14469 | 10.53 | 6.09E+06 | 13 |
| **Q9R111** | Guanine deaminase | *Gda* | 14544 | 2.42 | 1.06E+06 | 1 |
| **Q9CQM9** | Glutaredoxin-3 | *Glrx3* | 30926 | 3.56 | 1.48E+06 | 1 |
| **Q60631** | Growth factor receptor-bound protein 2 | *Grb2* | 14784 | 4.61 | 1.76E+06 | 1 |
| **P10922** | Histone H1.0 | *H1f0* | 14958 | 6.7 | 0.00E+00 | 4 |
| **P01902** | H-2 class I histocompatibility antigen, K-D alpha chain | *H2-K1* | 14972 | 6.25 | 6.50E+06 | 3 |
| **P14430** | H-2 class I histocompatibility antigen, Q8 alpha chain | *H2-Q8* | 15019 | 8.9 | 6.06E+06 | 5 |
| **Q61035** | Histidine--tRNA ligase, cytoplasmic | *Hars* | 15115 | 2.55 | 1.37E+06 | 2 |
| **P02088** | Hemoglobin subunit beta-1 | *Hbb-b1* | 15129 | 8.84 | 4.40E+06 | 3 |
| **P43276** | Histone H1.5 | *Hist1h1b* | 56702 | 10.31 | 8.08E+06 | 3 |
| **P13597** | Intercellular adhesion molecule 1 | *Icam1* | 15894 | 4.1 | 2.85E+06 | 2 |
| **P01868** | Ig gamma-1 chain C region secreted form | *Ighg1* | 16017 | 3.7 | 7.15E+06 | 3 |
| **P01872** | Ig mu chain C region | *Ighm* | 16019 | 3.52 | 4.98E+06 | 2 |
| **P01878** | Ig alpha chain C region | *Igh-VJ558* | 16061 | 4.65 | 1.58E+06 | 1 |
| **P01592** | Immunoglobulin J chain | *Igj* | 16069 | 9.43 | 7.73E+06 | 1 |
| **P01837** | Ig kappa chain C region | *Igkc* | 16071 | 13.21 | 2.98E+07 | 11 |
| **Q9QZ85** | Interferon-inducible GTPase 1 | *Iigp1* | 60440 | 15.25 | 1.04E+07 | 8 |
| **Q8CAQ8** | Mitochondrial inner membrane protein | *Immt* | 76614 | 1.45 | 2.56E+06 | 1 |
| **P49442** | Inositol polyphosphate 1-phosphatase | *Inpp1* | 16329 | 3.03 | 9.48E+05 | 2 |
| **O89112** | LanC-like protein 1 | *Lancl1* | 14768 | 2.01 | 1.42E+07 | 1 |
| **Q07797** | Galectin-3-binding protein | *Lgals3bp* | 19039 | 2.25 | 5.54E+06 | 3 |
| **P08071** | Lactotransferrin | *Ltf* | 17002 | 6.65 | 5.50E+06 | 8 |
| **P61327** | Protein mago nashi homolog | *Magoh* | 17149 | 7.53 | 2.59E+06 | 1 |
| **P34960** | Macrophage metalloelastase | *Mmp12* | 17381 | 2.11 | 6.40E+06 | 2 |
| **Q8BPB0** | MOB kinase activator 1B | *Mob1b* | 68473 | 5.09 | 9.16E+05 | 1 |
| **Q922D8** | C-1-tetrahydrofolate synthase, cytoplasmic | *Mthfd1* | 108156 | 1.39 | 2.38E+06 | 1 |
| **Q9JK81** | UPF0160 protein MYG1, mitochondrial | *Myg1* | 60315 | 3.16 | 1.26E+06 | 1 |
| **Q6PDN3** | Myosin light chain kinase, smooth muscle | *Mylk* | 107589 | 0.57 | 4.72E+05 | 1 |
| **P29595** | NEDD8 | *Nedd8* | 18002 | 13.58 | 7.51E+06 | 1 |
| **Q61937** | Nucleophosmin | *Npm1* | 18148 | 3.08 | 1.05E+06 | 2 |
| **Q02819** | Nucleobindin-1 | *Nucb1* | 18220 | 2.18 | 4.09E+05 | 1 |
| **P56812** | Programmed cell death protein 5 | *Pdcd5* | 100042424 | 9.52 | 9.25E+05 | 1 |
| **Q62048** | Astrocytic phosphoprotein PEA-15 | *Pea15* | 18611 | 7.69 | 4.61E+05 | 1 |
| **O70591** | Prefoldin subunit 2 | *Pfdn2* | 18637 | 7.79 | 2.76E+06 | 1 |
| **Q8CHP8** | Phosphoglycolate phosphatase | *Pgp* | 67078 | 4.67 | 1.65E+06 | 2 |
| **O70570** | Polymeric immunoglobulin receptor | *Pigr* | 18703 | 1.95 | 5.33E+06 | 2 |
| **A3KGF7** | 1-phosphatidylinositol 4,5-bisphosphate phosphodiesterase beta-2 | *Plcb2* | 18796 | 0.68 | 3.04E+06 | 2 |
| **Q9Z2M7** | Phosphomannomutase 2 | *Pmm2* | 54128 | 4.96 | 1.11E+06 | 1 |
| **Q9D819** | Inorganic pyrophosphatase | *Ppa1* | 67895 | 5.19 | 9.12E+06 | 5 |
| **Q9CR16** | Peptidyl-prolyl cis-trans isomerase D | *Ppid* | 67738 | 2.43 | 1.43E+06 | 1 |
| **Q61207** | Prosaposin | *Psap* | 19156 | 1.8 | 1.01E+07 | 5 |
| **O35955** | Proteasome subunit beta type-10 | *Psmb10* | 19171 | 7.69 | 4.74E+06 | 3 |
| **Q9EPB4** | Apoptosis-associated speck-like protein containing a CARD | *Pycard* | 66824 | 8.29 | 7.63E+05 | 2 |
| **Q8BND5** | Sulfhydryl oxidase 1 | *Qsox1* | 104009 | 1.47 | 9.88E+05 | 1 |
| **P62492** | Ras-related protein Rab-11A | *Rab11a* | 53869 | 6.02 | 1.60E+06 | 2 |
| **Q9EP95** | Resistin-like alpha | *Retnla* | 57262 | 13.51 | 1.36E+07 | 5 |
| **Q9CZX8** | 40S ribosomal protein S19 | *Rps19* | 20085 | 6.21 | 3.00E+05 | 1 |
| **P62908** | 40S ribosomal protein S3 | *Rps3* | 27050 | 5.35 | 1.96E+06 | 2 |
| **P14206** | 40S ribosomal protein SA | *Rpsa* | 16785 | 5.76 | 4.31E+06 | 1 |
| **Q01730** | Ras suppressor protein 1 | *Rsu1* | 20163 | 3.25 | 1.76E+06 | 1 |
| **P56565** | Protein S100-A1 | *S100a1* | 20193 | 13.83 | 3.12E+06 | 1 |
| **Q60710** | Deoxynucleoside triphosphate triphosphohydrolase SAMHD1 | *Samhd1* | 56045 | 7.66 | 3.61E+06 | 5 |
| **P07759** | Serine protease inhibitor A3K | *Serpina3k* | 20714 | 5.98 | 2.84E+06 | 2 |
| **P97290** | Plasma protease C1 inhibitor | *Serping1* | 12258 | 2.18 | 3.47E+06 | 1 |
| **P35242** | Pulmonary surfactant-associated protein A | *Sftpa1* | 20387 | 11.29 | 1.47E+07 | 9 |
| **Q64105** | Sepiapterin reductase | *Spr* | 20751 | 5.75 | 3.34E+06 | 2 |
| **P13609** | Serglycin | *Srgn* | 19073 | 10.53 | 1.31E+07 | 4 |
| **Q62093** | Serine/arginine-rich splicing factor 2 | *Srsf2* | 20382 | 3.62 | 1.22E+06 | 1 |
| **P37804** | Transgelin | *Tagln* | 21345 | 5.47 | 5.42E+05 | 1 |
| **P10711** | Transcription elongation factor A protein 1 | *Tcea1* | 21399 | 3.32 | 2.07E+06 | 1 |
| **P21981** | Protein-glutamine gamma-glutamyltransferase 2 | *Tgm2* | 21817 | 2.19 | 4.52E+06 | 2 |
| **Q9QZE7** | Translin-associated protein X | *Tsnax* | 53424 | 5.86 | 3.05E+06 | 1 |
| **Q9Z0P5** | Twinfilin-2 | *Twf2* | 23999 | 2.87 | 8.69E+05 | 1 |
| **Q02053** | Ubiquitin-like modifier-activating enzyme 1 | *Uba1* | 22201 | 1.42 | 2.05E+06 | 2 |
| **Q9Z1Z0** | General vesicular transport factor p115 | *Uso1* | 56041 | 1.04 | 6.92E+05 | 1 |
| **Q9Z1Q9** | Valine--tRNA ligase | *Vars* | 22321 | 0.95 | 3.88E+06 | 2 |

**Table S8**: Primer sequences for real-time RT-PCR.

| **Gene symbol** | **Forward (5’→3’ plus strand)** | **Reverse (5’→3’ minus strand)** |
| --- | --- | --- |
| ***Actb*** | CTTCTTTGCAGCTCCTTCGT | TCCTTCTGACCCATTCCCAC |
| ***Pigr*** | GTGCCCGAAACTGGATCACC | TGGAGACCCCTGAAAAGACAGT |
